# The Long-Run Socio-Economic Consequences of a Large Disaster: The 1995 Earthquake in Kobe

**DOI:** 10.1371/journal.pone.0138714

**Published:** 2015-10-01

**Authors:** William duPont IV, Ilan Noy, Yoko Okuyama, Yasuyuki Sawada

**Affiliations:** 1 Economics Department, College of St Benedict|St John’s University, St. Joseph, MN, United States of America; 2 School of Economics and Finance, Victoria University of Wellington, Wellington, New Zealand; 3 Department of Economics, Yale University, New Haven, CT, United States of America; 4 Graduate School of Economics, The University of Tokyo, Tokyo, Japan; 5 Research Institute of Economy, Trade and Industry, Tokyo, Japan; Hamamatsu University School of Medicine, JAPAN

## Abstract

We quantify the ‘permanent’ socio-economic impacts of the Great Hanshin-Awaji (Kobe) earthquake in 1995 by employing a large-scale panel dataset of 1,719 cities, towns, and wards from Japan over three decades. In order to estimate the counterfactual—i.e., the Kobe economy without the earthquake—we use the synthetic control method. Three important empirical patterns emerge: First, the population size and especially the average income level in Kobe have been lower than the counterfactual level without the earthquake for over fifteen years, indicating a permanent negative effect of the earthquake. Such a negative impact can be found especially in the central areas which are closer to the epicenter. Second, the surrounding areas experienced some positive permanent impacts in spite of short-run negative effects of the earthquake. Much of this is associated with movement of people to East Kobe, and consequent movement of jobs to the metropolitan center of Osaka, that is located immediately to the East of Kobe. Third, the furthest areas in the vicinity of Kobe seem to have been insulated from the large direct and indirect impacts of the earthquake.

## Introduction

**Fig 1:**
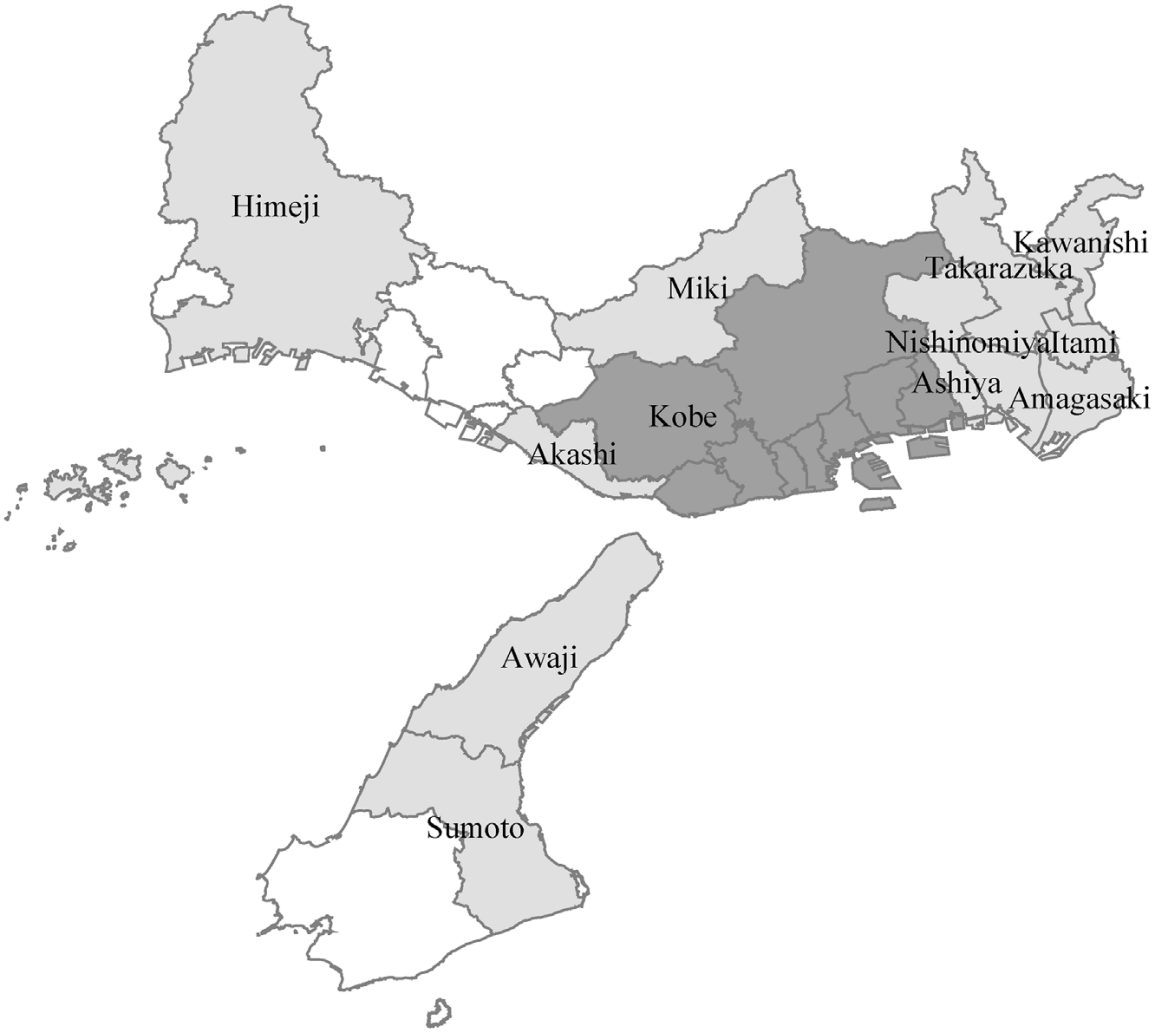
Hyogo Prefecture.

**Fig 2:**
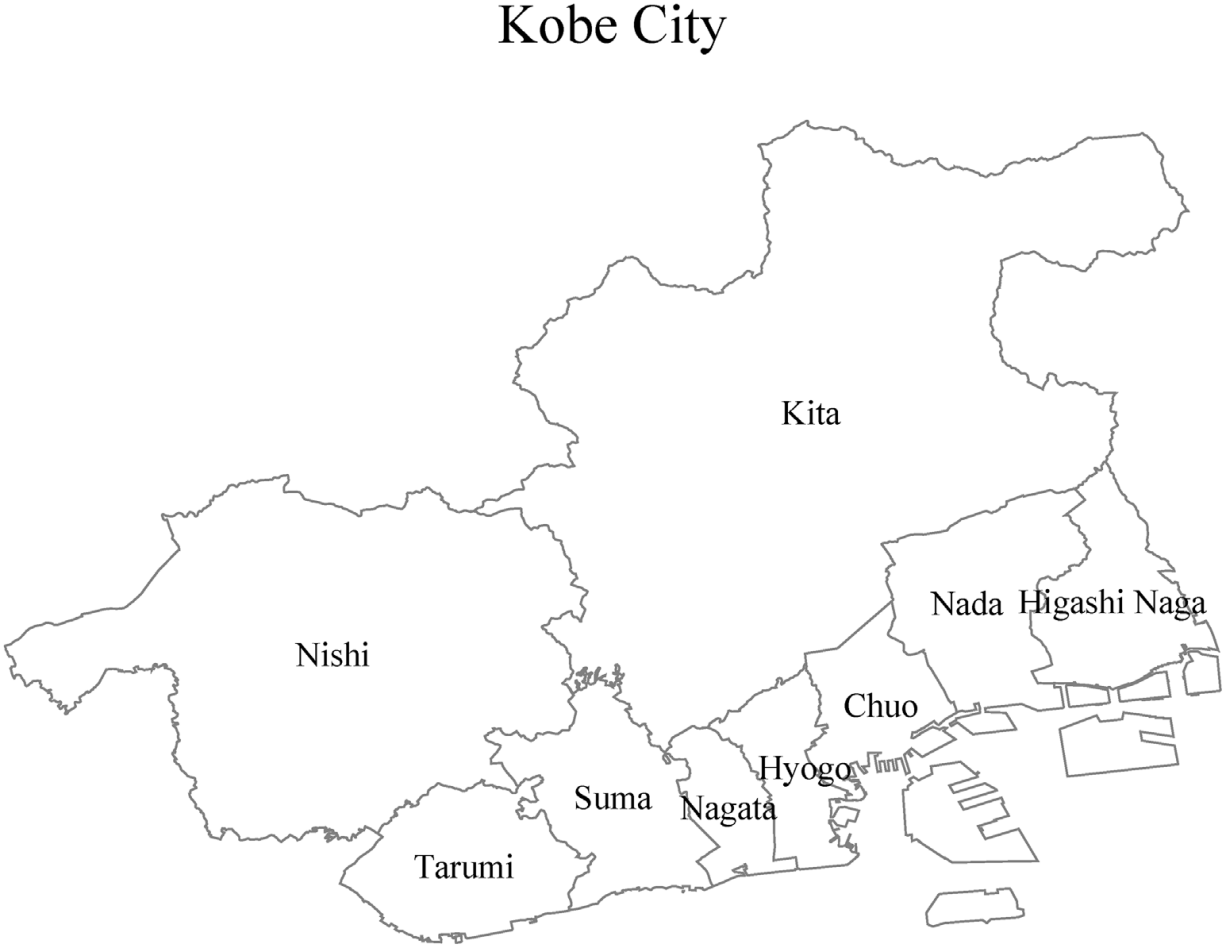
Wards in Kobe City.

The Great Hanshin-Awaji earthquake (hereafter, the Kobe earthquake) struck at 5:46 a.m. on January 17, 1995, on Awaji Island, in Japan’s Hyogo prefecture (Fig 1) offshore from Hyogo’s main metropolitan center, the city of Kobe (Fig 2). The earthquake affected an area that was, at the time, home to 4 million people and contained one of Japan’s main industrial clusters. The earthquake, which had registered 7.3 on the Richter scale, cost 6,432 lives, resulted in 43,792 injured, and damaged 639,686 buildings, of which 104,906 were completely destroyed [1]. The Kobe earthquake was responsible for one of the largest direct economic losses due to a natural hazard in recorded human history. While we understand well the direct impact of the Kobe earthquake, we know much less about its impacts in the long-term. Surveys suggest that the people of Kobe experienced a prolonged and significant adverse impact on their well-being [2–3]. We know a lot less about Kobe’s economic recovery. Did the Kobe earthquake in 1995 indeed cause permanent losses to the economies of Kobe and other surrounding areas? Or can the recorded sense of deteriorating well-being be explained through mechanisms other than a real decline in the economic circumstances of the region?

Some early papers concluded that the devastation wrought by the 1995 Kobe earthquake did not have any long-term impact on the Japanese economy, nor much impact on Kobe itself [4], though others were less sanguine about the disasters impact [5]. We discuss this literature more thoroughly in the next question. Ultimately the answer to this question should be based on a comparison between the actual Kobe economy, and a counter-factual Kobe without the earthquake. The conventional approach has been to compare the development of post-quake Kobe with the trends observed in Japan excluding Kobe [6]. However, such an approach raises questions about the arbitrariness of selection and the degree to which the comparison unit (Japan excluding Kobe) is indeed a credible proxy for Kobe’s counterfactual (the city without an earthquake). This difficulty is compounded by the fact that the earthquake occurred a few years after Japan had entered the “lost decade”—a prolonged recession following the collapse of the housing market circa 1990.

The synthetic control method we adopt here, introduced by [7–8], overcomes these shortcomings by adopting a data-driven control-group selection procedure. At its most basic, the counter-factual observations are constructed as a weighted average of available control units that were not affected. Thus, this synthetic control approximates the most relevant characteristics of the treated unit prior to the treatment. In our case, the counterfactual Kobe without an earthquake was constructed from a weighted average of cities and towns that were not directly affected by the earthquake and were far away from its epicenter.

The 1995 Kobe earthquake was maybe the first large scale disaster to affect a high income industrialized economy; and the decades since have seen several other large disasters in high income countries so that disasters are seen today not only as a risk for lower income developing countries. Hurricane Katrina in 2005, and the earthquakes in Chile in 2010 and Japan and New Zealand in 2011 are all examples of this risk. As such, the last few years have seen renewed interest in understanding what happened to Kobe in the aftermath of its earthquake, and it is to this literature that we are contributing. In the next section, we discuss previous research on Kobe, and its conclusions; in section 3 we provide more detail about our data and methodology we use; and in section 4 we describe our results.

## Research on the 1995 Kobe Earthquake

Recently, two papers, using different methodologies and Japanese prefecture (province/state) level datasets, reached opposing conclusions about the prefecture-level economic long-term impact of the quake [9–10]. The literature, thus, is far from reaching a consensus conclusion about the aggregate impact of the earthquake on economic activity, even at the prefecture level. Neither, of course, can these two papers explain their findings, as the analysis of prefecture level data masks dramatic heterogeneities in impacts within Hyogo prefecture in the amount of direct losses with much of Kobe City and surrounding areas dramatically damaged, but the rest of the prefecture largely unaffected. Equally, we would expect significant heterogeneities in the long-term indirect impacts for these separate geographical areas within the prefecture.

Davis and Weinstein, in a set of influential papers, investigated the long-term impact of large-scale war-related destruction in Japan using detailed bombing data from World War II [11–12]. In these two papers, they found no long-term impact of the bombing campaigns on the level and distribution of economic activity many years later. Yet, research examining the impact of disasters on Japanese localities is generally less sanguine.

Today, there is a large literature looking at the short-term impact of catastrophic events such as natural disasters and terrorism events on various economic (growth, poverty, inflation, employment; e.g. [13]), social (well-being, social capital; e.g [14]), and demographic (population composition, fertility; e.g. [15–16]) variables. The natural disaster literature already includes several regression meta-analyses; e.g. [17–18]. Still, the literature on the long-term impact of economic shocks is relatively sparse, but the weight of the evidence suggests no lasting impact of even catastrophic shocks at the national level [19] but significant impacts at more local/regional levels in terms of even very long-term recovery–[20] analyses the impact of an event 50 years after the fact.

On Kobe, [21] reports re-distribution of population and economic activity across city districts, and thus of aggregate economic activity. [22] also analyzes population dynamics across the geography of the city, and includes a qualitative and richly detailed exploration of the policy decisions and their consequences across the city. Both papers find that the geography of recovery is quite complex with some areas experiencing post-disaster booms (in terms of population) and others struggling.

Additional papers focus on other aspects of recovery in Kobe. [23], for example, finds that investment in social capital has also increased in the quake’s aftermath. [5–6] examine the time-series data for Kobe following the disaster, and measure the impact of the event by comparing the Kobe dynamics to what happened to the national economy (i.e., implicitly assuming the Kobe would have followed nation-wide trends without the disaster). Both papers note a long-term adverse effect on gross regional product of the Kobe region. In [6], however, this effect dissipates by 2005. [5] largely compares Kobe to its pre-quake state, and her methodology does not account for the changes in the Japanese economic potential that occurred in the 1990s and 2000s and that may have been unique to Kobe and other regions with similar economic structure.

The micro-econometric literature includes several papers that examine labor and income data at the individual/household level. They conclude that the Kobe Earthquake had long-lasting adverse effects on individuals, groups, and households in this region [24–28]. [29] examines the impact of the earthquake on industrial creative destruction, business continuity and firm survival. [30–31] both focus on more short term impacts, but describe some of the mechanisms that may have led to the long-term impacts that we describe later. [30] focuses on the spatial distribution of housing reconstruction post 1995, while [31] examines the recovery, or lack-thereof, of the Kobe port.

Besides data-driven regression analysis, several papers have opted to use other methodologies to examine the impact of the Kobe earthquake. Of particular interest in the input-output analysis conducted by [32]. Input-output analysis is static by construction, however, unless the designer of the model allows for some structural mechanism for change, as is done in [32]. The main advantage of input-output analysis is that it permits a lot of detail about the sectoral decomposition of impacts; yet, it lacks a mechanism to critically examine the obtained results and interpret them. As such, it should be seen as a method that complements our own findings, rather than aims to replace them.

Here, we aim to establish a counterfactual with enough geo-spatial detail to provide insights on the heterogeneous ways in which the economy of the region was impacted, and thus assist in attempts to describe the mechanisms that led to these long-term effects. We employ a large panel data of Japanese cities, towns, and wards (districts/counties) observed annually for over three decades (1980–2010).

## Data & Method

We collected data for all Japanese cities, towns, and wards. Between 1980 and 2010 there were 719 mergers between cities and towns. This, together with missing observations, reduced our sample to 1719 cities, towns, and wards. For all these, we obtain information on 67 variables, so that our dataset is constructed from 1,763,153 observations. Due to missing data, however, we were only able to use 53 of the 67 variables (46 of 67 when we were examining wards).

Since we wanted our synthetic counterfactual to be representative of Kobe and other cities from an economic standpoint we selected variables which theory would suggest have influence on the makeup of the local economy. These variables fell into 6 categories; demographic (such as population), environmental (such as habitable land area), economic (such as total taxable income), government (such as government expenditure), labor (such as number of unemployed), and spatial-economic (such as number of retail shops). 34 of the variables that we used were collected by the census bureau in Japan, and thus have a frequency of every 5 years. The remainder of the data was collected by various Japanese organizations and ministries and had varying frequencies of 1, 3 and 5 years. All of this data is available through: http://www.stat.go.jp/english/data/ssds/outline.htm.

One of the advantages of the synthetic control methodology is that additional variables act as descriptors or observations that provide additional information about how the constructed synthetic matches the treated unit. Since these variables act only as observations and do not otherwise interact with one another, it is not important if their timing matches. Therefore, we do not a priori rule out the use of the any of the variables available in the dataset. Our choice is mostly dictated thus by data availability. Thus all 53 variables (46 when we examined wards) are used in vector *Z*.

The synthetic control methodology defines a treated unit, in this case every city and town that was directly affected by the earthquake, and the identification of non-treated units, in this case all Japanese cities and towns that were not directly affected and are not in Hyogo or Osaka prefectures (as these may have been affected indirectly).

We employ the synthetic control methodology to quantify the impact of the Kobe earthquake by constructing a counterfactual as a weighted average of all Japanese cities and towns that have not been directly (or indirectly) affected by the earthquake. Let *Y*_*it*_ be the outcome variable for city (town/ward) *i*, where we set *i* = 1 for the treated city and *i*>1 for the other Japanese cities unaffected directly by the earthquake, at time *t* = (1,…,*T*_0,_…*T*) where *T*_0_ = 1994 –the description of the methodology, below, is a modified description of [8]. $Y_{it}^{I}$ is the outcome variable in the presence of the earthquake and $Y_{it}^{N}$ is the outcome variable had the earthquake not occurred. The model requires the assumption that the event had no effect on the outcome variable before it occurred at time $T_{0}+1\;\left(Y_{it}^{I}=Y_{it}^{N}\forall t\leq T_{0}\right)$. Although this last assumption is unjustified in cases where disaster impact is frequent and therefore expected, Kobe had not experienced a similar event, and was widely perceived as a low-earthquake-risk region.

The observed outcome is defined by $Y_{it}=Y_{it}^{N}+\alpha_{it}D_{it}$ where *α*_*it*_ is the effect of the disaster on the variable of interest, and *D*_*it*_ is the binary indicator denoting the event occurrence (*D*_*it*_ = 1 for *t*>*T*_0_ and *i* = 1; and *D*_*it*_ = 0 otherwise). The aim is to estimate *α*_*it*_ for all *t*>*T*_0_ for the affected cities (*i* = 1). The estimation problem is that for all *t*>*T*_0_ it is not possible to observe $Y_{1t}^{N}$ (the counterfactual).

Following [8], suppose that $Y_{it}^{N}$ can be given by the following factor model: $Y_{it}^{N}=\delta_{t}+\theta_{t}Z_{i}+\lambda_{t}\mu_{i}+\varepsilon_{it}$, where Z_*i*_ is a vector of observed covariates (variables such as regional product per capita and population) and *μ*_*i*_ is a vector of unknown city-specific idiosyncratic effects. Let *W* = (*ω*_2_,…,*ω*_*j+1*_) be a vector of weights allocated to the different (unaffected) city observations such that *ω*_*j*_≥0 for *j* = 2,…,*J*+1 and $\sum_{j=2}^{j+1}\omega_{j}=1$. A synthetic control is a weighted combination of the controls such that it replicates a treated unit as if the treatment had not occurred. Thus the outcome variable for each synthetic control can be written

$$\sum_{j=2}^{j+1}\omega_{j}Y_{jt}=\delta_{t}+\theta_{t}\sum_{j=2}^{j+1}\omega_{j}Z_{j}+\lambda_{t}\sum_{j=2}^{j+1}\omega_{j}\mu_{j}+\sum_{j=2}^{j+1}\omega_{j}\varepsilon_{jt}$$ (1)

Suppose there is a set of estimated weights $\left({\hat{\omega }}_{2},\ldots ,{\hat{\omega }}_{J+1}\right)$ that can accurately replicate the treated unit’s pre-treatment observations in the following manner

$$\sum_{j=2}^{j+1}{\hat{\omega }}_{j}Y_{j1}=Y_{11},\ldots ,\sum_{j=2}^{j+1}{\hat{\omega }}_{j}Y_{jT_{0}}=Y_{1T_{0}}\;and\sum_{j=2}^{j+1}\hat{\omega_{j}}Z_{j}=Z_{1}$$ (2)

[8] show that under acceptable assumptions, combining the previous equations yields the following:$Y_{1t}^{N}=\sum_{j=2}^{j+1}{\hat{\omega }}_{j}Y_{jt}$. They further show that this equality will hold for all *t* provided the number of pre-intervention periods is large enough. While there are 14 years of pre-disaster data, which is comparable to [7] and [8], our data is available in variable frequency over this time period. Some of the variables are only available in 5-year gaps, and we therefore have only 3 observations for them, while others are available for either 9 or 14 annual pre-treatment time periods. We obtain an estimate of the impact of treatment (the earthquake) as:

$${\hat{\alpha }}_{1t}=Y_{1t}-\sum_{j=2}^{J+1}{\hat{\omega }}_{j}Y_{jt}\;f\;or\;t>T_{0}$$ (3)

Our goal is to select a set of weights for which (2) holds approximately. We determine the appropriate weights by examining the goodness of fit over the pre-treatment period as well as the predictor balance for all of the variables in *Z*_1_.

From Eq (2), one of the key requirements of the synthetic control methodology is that not only should the counterfactual match the variable of interest during the pretreatment period, but it should also be able to match each of the predictor variables as well. The predictor balance is a table that shows the value of each of the predictor variables for both the variable of interest and the synthetic control. This information is available from the authors upon request.

The set of weights *W* is selected to minimize the distance between the predictor variables for the treated city (*X*_1_) and those of the synthetic control (*X*_0_ *W*) during the pretreatment period. We choose *W* so that the following is minimized: ${\left|\left|X_{1}-X_{0}W\right|\right|}_{V}=\sqrt{\left(X_{1}-X_{0}W\right)\prime V\left(X_{1}-X_{0}W\right)}$ where *V* is a (*k* × *k*) symmetric and positive semi-definite matrix. In this particular case *k* is the number of explanatory variables. *V* is used to place weights on the predictor variables such that the difference between the variable of interest for the treated city and that of the synthetic control is minimized during the pre-treatment period.

We use the Synth Package for R to obtain *V* such that the root mean squared prediction error (RMSPE) is minimized for the period prior to the earthquake. For robustness, we use two different initial values to obtain *V* and then use the result with the best fit as our final value. We only present, and map, results for which the *RMSPE* ≤ 10% $\left(RMSPE\equiv \sqrt{\frac{{\sum^{}}_{t}{\left(\frac{y_{t}-{\hat{y}}_{t}}{{\hat{y}}_{t}}\times 100\right)}^{2}\times 1_{\left\{t<1994\right\}}}{{\sum^{}}_{t}1_{\left\{t<1994\right\}}}}\right)$. The imposed condition of *RMSPE* ≤ 10% is similar to the one implemented in other papers using synthetic control. We calculate the RMSPE for the entire pre-treatment period, so we therefore do not account for changing fit over time (for example, for worsening fit in the immediate pre-treatment period). The motivation for strict adherence to this condition is that this tight fit establishes the robustness of our results. Our success (or lack thereof) in establishing a counterfactual that successfully tracks the actual observations for the treated units in the pre-treatment period is our main yardstick.

A plausible way to examine the statistical robustness of synthetic control estimates is to examine placebo impacts (impact assessment for geographical units that were not, in reality, affected by the disaster—similarly to the placebo effect in medical studies). This approach is, however, difficult in our case, given the very large dataset we are using (much bigger than what was used in the previously cited papers [7–8]). We nevertheless include placebo results for our main variables of interest in (S1 Fig) and discuss them in the text below.

## Results

**Fig 3:**
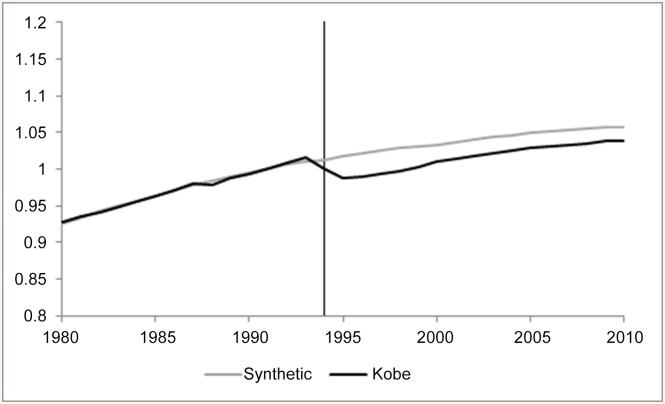
The Impact of the Earthquake on Population of Kobe City.

**Fig 4:**
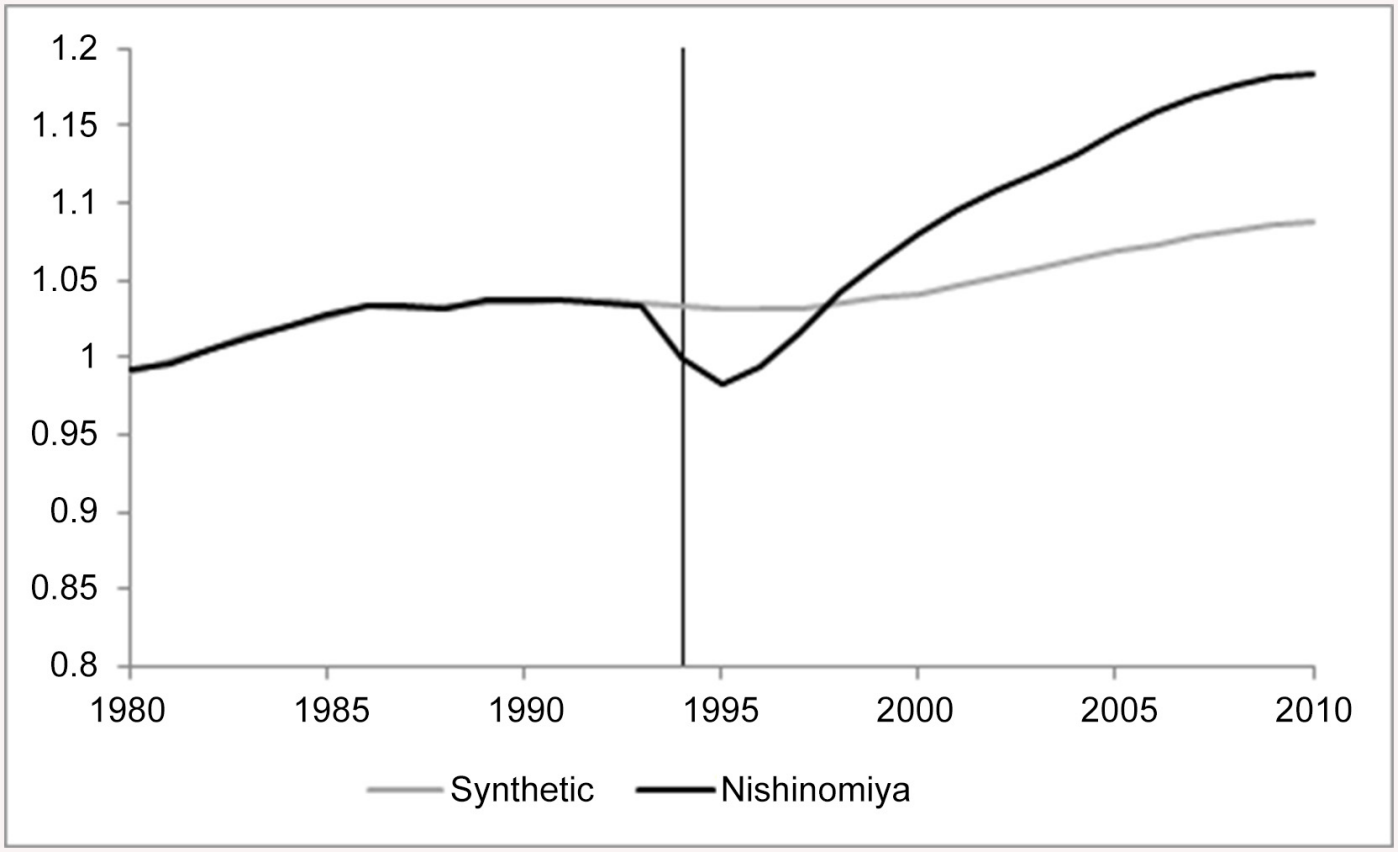
The Impact of the Earthquake on Population of Nishinomiya City.

We start by showing a few illustrative examples of the results we obtain, and follow with a set of maps that summarize our results more comprehensively. In (Figs 3 and 4), we show the impact of the earthquake on the population of Kobe City (aggregated over its wards), and for another nearby city to the East of Kobe, Nishinomiya. These two figures show both the actual observations for Kobe and Nishinomiya (black lines) over the whole sample period (1980–2010) and the calculated synthetic counterfactual (grey line). The vertical distance between the two lines is the calculated impact of the earthquake (${\hat{\propto }}_{1t}$). The units on the vertical axis are standardized to equal one at the time of the earthquake. Thus a line at 1.1 a decade later implies a 10% increase.

In this particular example, only 4 cities received positive weights to serve as the counterfactual for the city of Kobe. They were Kitakyushu (45%), Sapporo (40%), Nagoya (14%) and Yokohama (1%). An advantage of the synthetic control methodology is its built-in transparency—the ability to check whether or not the locations that make up the counterfactual have similar qualities to the affected location. Three of these cities are important coastal cities in Japan. The one exception is Sapporo, which gains access to the coast through the cities of Otaru and Ishikari. Two of these cities, Nagoya and Yokohama, are major Japanese port cities, and three are the designated capital of their respective prefectures (the exception is Kita-Kyushu). As Kobe serves as a satellite of Osaka, this is mirrored by Yokohama, which is a satellite of Tokyo, and to a far lesser degree Kita-Kyushu and nearby Fukuoka. In short, these cities are similar to Kobe in many ways, so that this matching exercise appears to be reasonable on both quantitative and qualitative grounds.

For Kobe City, we find permanent negative but small impact on total population: around 2% decline in population 15 years after the earthquake, after an initial larger decline in the immediate disaster’s aftermath (Fig 3). Similar figures available in (S1 Fig) show that the permanent loss of population in Kobe City can be identified for both males and females. Nishinomiya, shown in (Fig 4), provides an illuminating contrast. After a sharp decline in the immediate aftermath of the earthquake, a decline that was bigger than that experienced in Kobe City, Nishinomiya ended up with population gain; the population 15 years after the earthquake has increased by 10% relative to what it would have been had the earthquake not occurred.

For Kobe’s population estimate, we make two observations from the placebo results: First, the goodness-of-fit for Kobe’s population estimates pre-event is better than for most other cities in our dataset. Second, the post-event trajectory of Kobe is not significantly outside the range of estimates for other regions. This second observation suggests that the small identified impact on Kobe’s population indeed does not indicate a statistically robust and large deviation from its expected trend.

(Figs 3 and 4) show the impact of the earthquake for only two geographical units. In order to summarize the information included in the results for every impacted city/town/ward in the region, we plot these on a map. We color every geographical unit with the estimated impact on the variable of interest, calculated as the difference between the synthetic and the actual observation for that region (as the distance between the two lines in (Figs 3 and 4) expressed in percent); blue colors denote decreases and the reds denote increase. Only those results for which the pre-event fit is sufficient (*RMSPE* ≤ 10%; see footnote 7) are presented. These maps allow us to observe more clearly the spatial patterns we found. In all figures, the top panel presents our estimates using the city-level data. Thus, the impact plotted for Kobe City is estimated for the city as a whole, using a control group composed of other Japanese cities. The bottom panel provides more detail by focusing on differential impacts across the nine wards of Kobe City; these impacts are estimated using the ward-level dataset.

**Fig 5:**
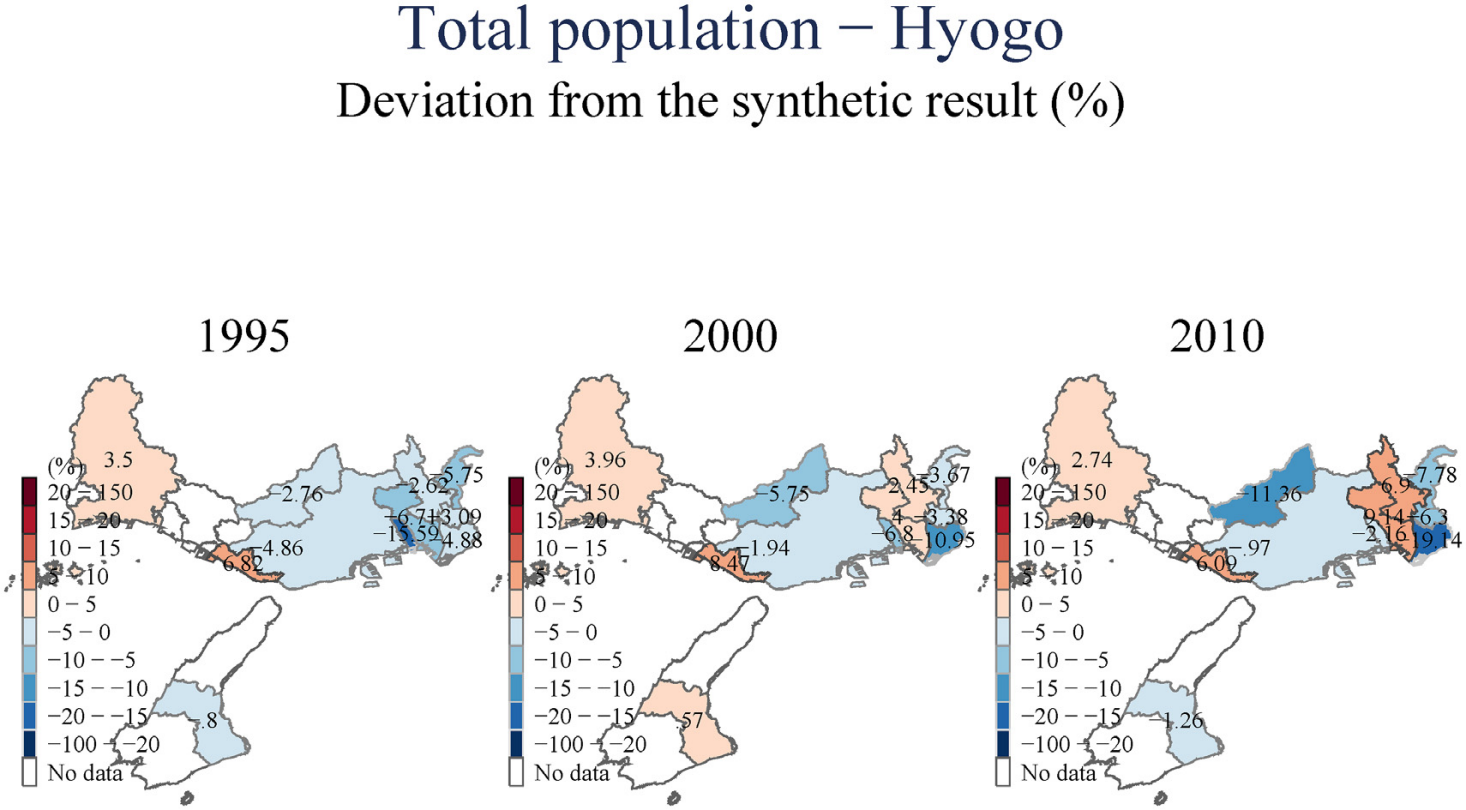
Total population—Hyogo.

**Fig 6:**
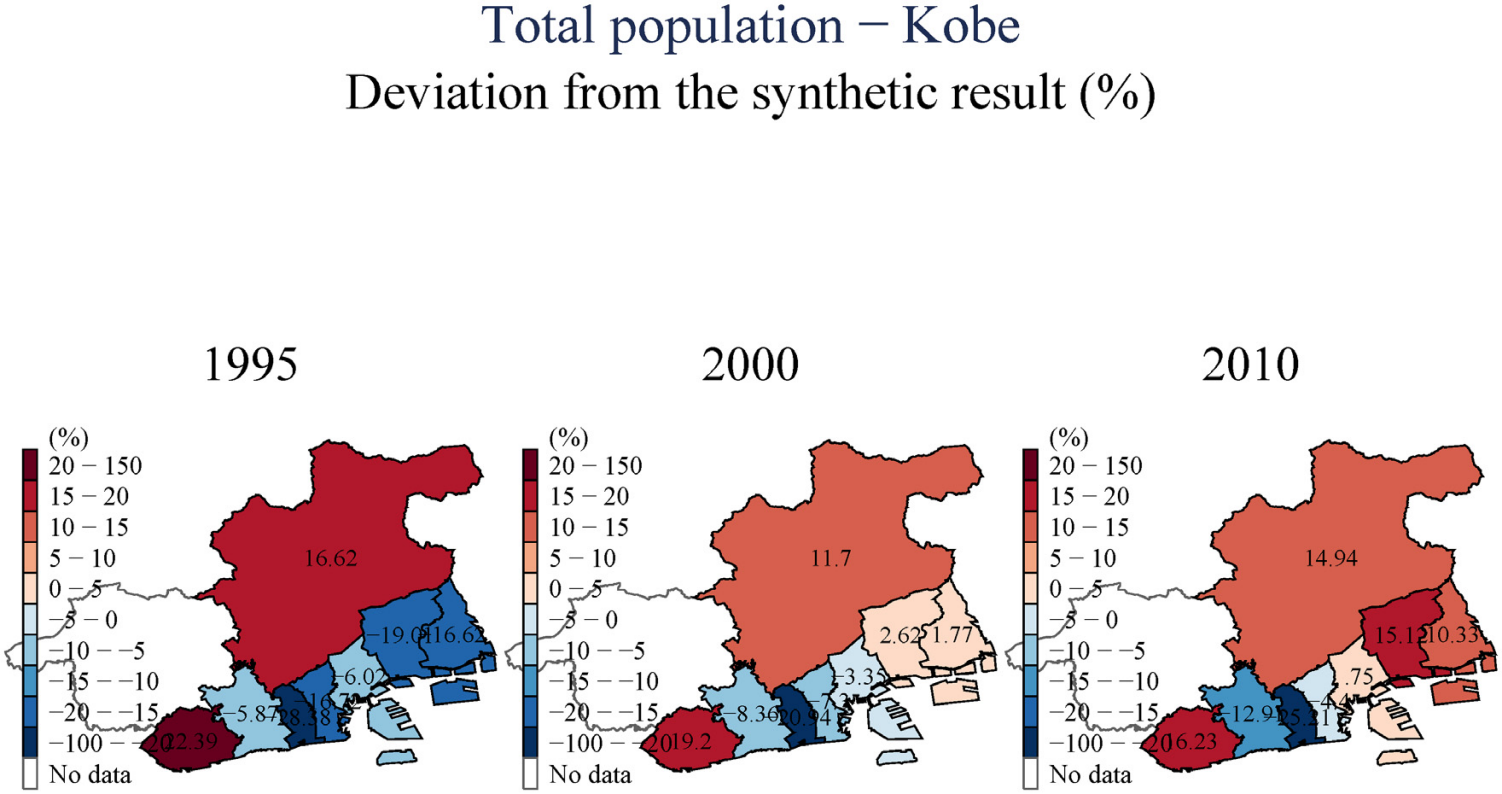
Total population—Kobe.

**Fig 7:**
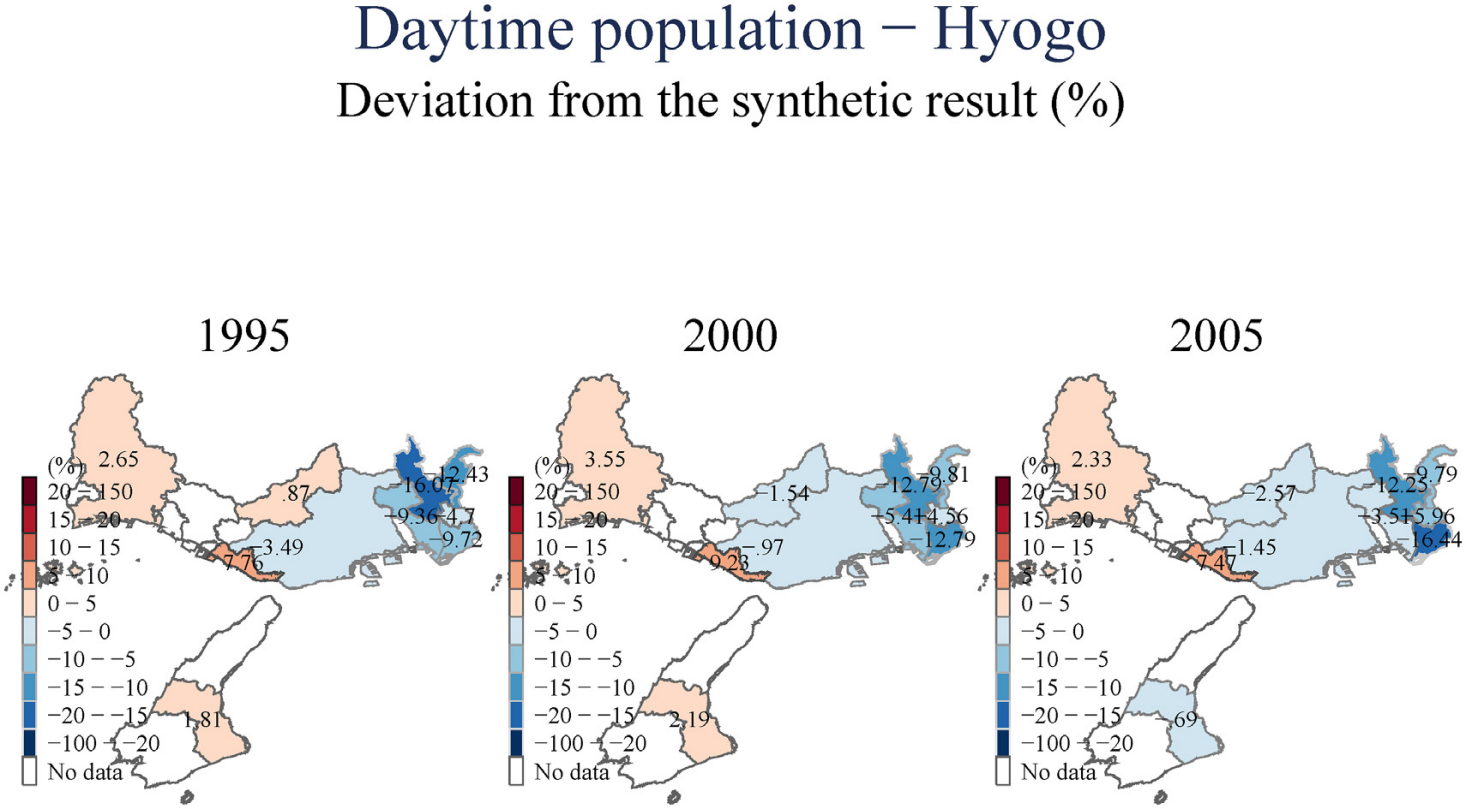
Daytime population—Hyogo.

**Fig 8:**
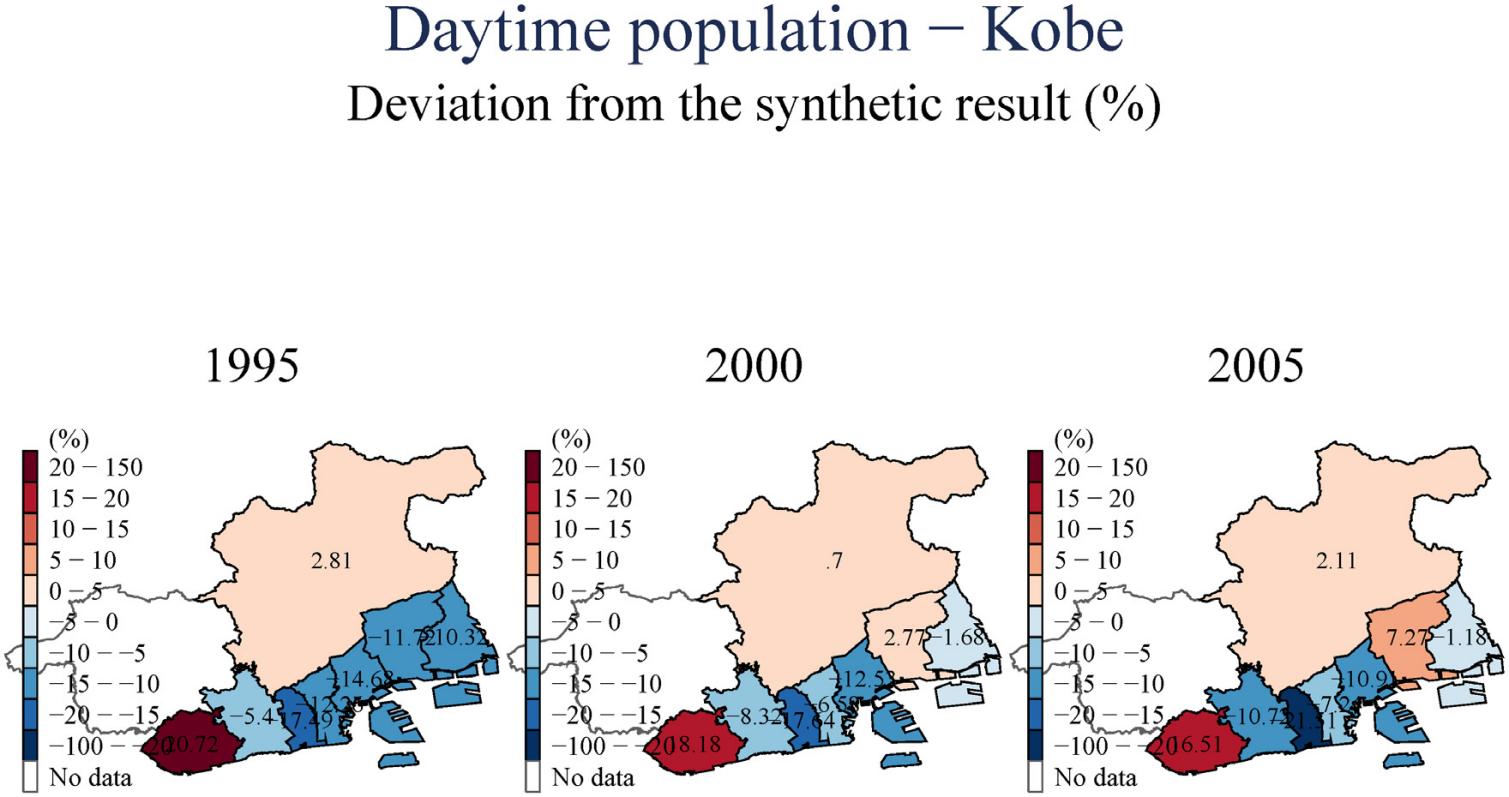
Daytime population—Kobe.

During the first year after the earthquake, there was a short-term dip in population across the whole area nearest to the epicenter, and including the urban Eastern corridor toward Osaka. In the longer-run, however, we observe heterogeneities in permanent population trends. Figures available in (S1 Fig) present the population impact maps for the aggregate figures, and disaggregated by gender and age and using several population measures from different sources. In (Figs 5 and 6), we observe a pattern of movement away from the most severely affected areas. However, regions to the east, that were also seriously impacted initially, seem to gain in long run, suggesting that proximity to Osaka may be a driver of population recovery. These patterns are not uniform; Sumoto city, for example, which is located near the epicenter, has been largely unaffected in the long-run, implying that the community and industry employment characteristics matter as well. One possibility, elaborated on by [14], is that the community is a major determinant of these differing recovery trajectories, and that cohesive communities recover faster and more completely. [29] on the other hand, emphasize industry/sector characteristics as determinants of recovery trajectories. Our data does not allow us to distinguish between these differing explanations, and it is likely that they all interact in complex ways to determine outcomes.

(Figs 7 and 8) include an examination of the day-time population of the area we examine. These estimates suggest that there is a uniform and persistent decline of population even in the longer-term. This decline in daytime population is even observed for towns to the East, for which we observed population increases in (Figs 5 and 6). This suggests that the increase in population observed to the East of Kobe City is driven by people who have moved to these areas from the devastated center, but have also switched their location of employment eastward to Osaka.

**Fig 9:**
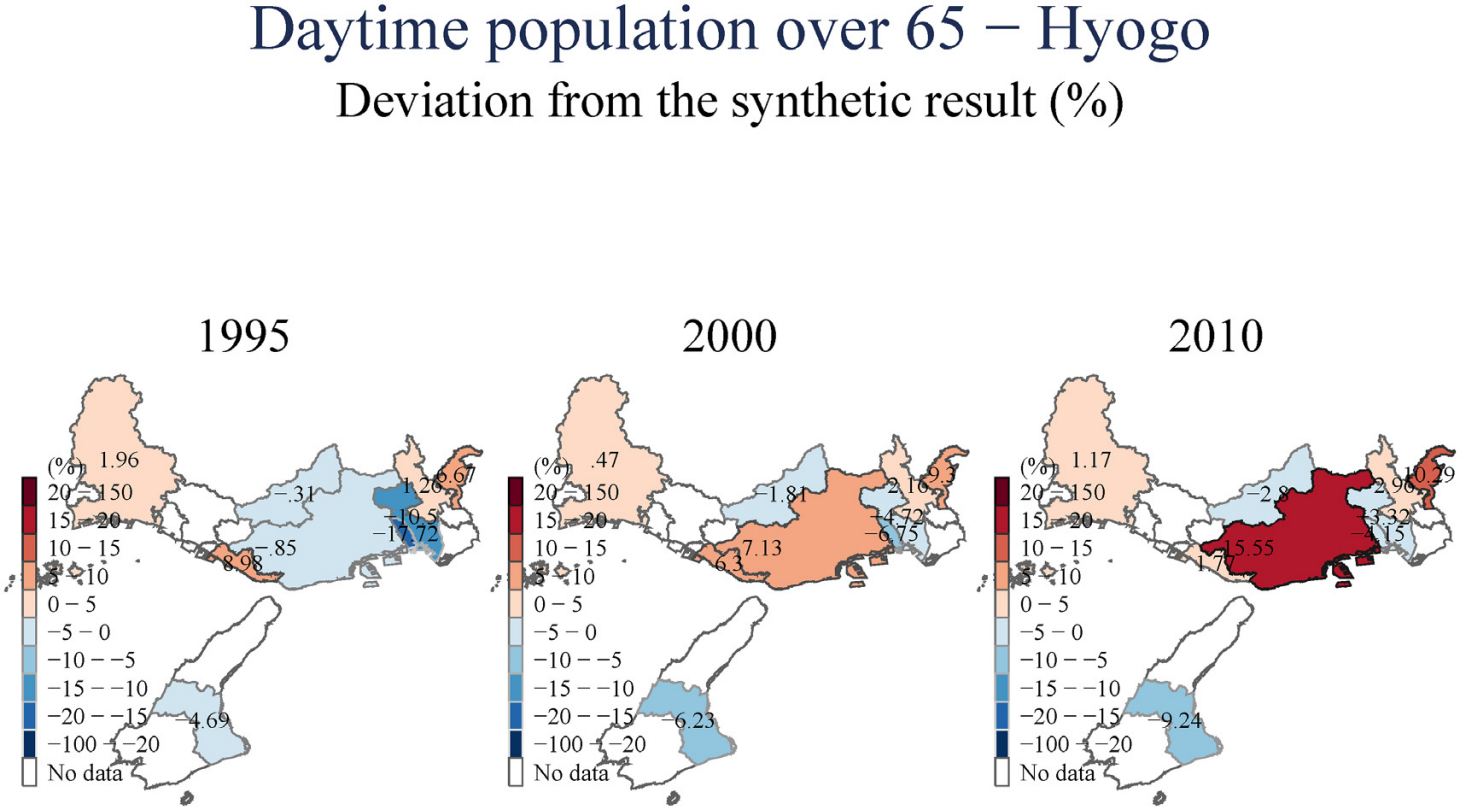
Daytime population over 65 –Hyogo.

Another intriguing trend, presented in (Fig 9), is the increase in the number of people over the age of 65. When compared with other geographical units in Japan (the synthetic control), Kobe City seemed to have gained more. While we do not know the exact reasons for this shift, we can speculate that it may be associated with either people returning to their cultural roots (as the impact of the earthquake leads to shifts in preferences), or that over-65, living mostly on fixed incomes, are moving to a place where living costs are more manageable (both because of the relative economic decline of the region and the generous government support).

**Fig 10:**
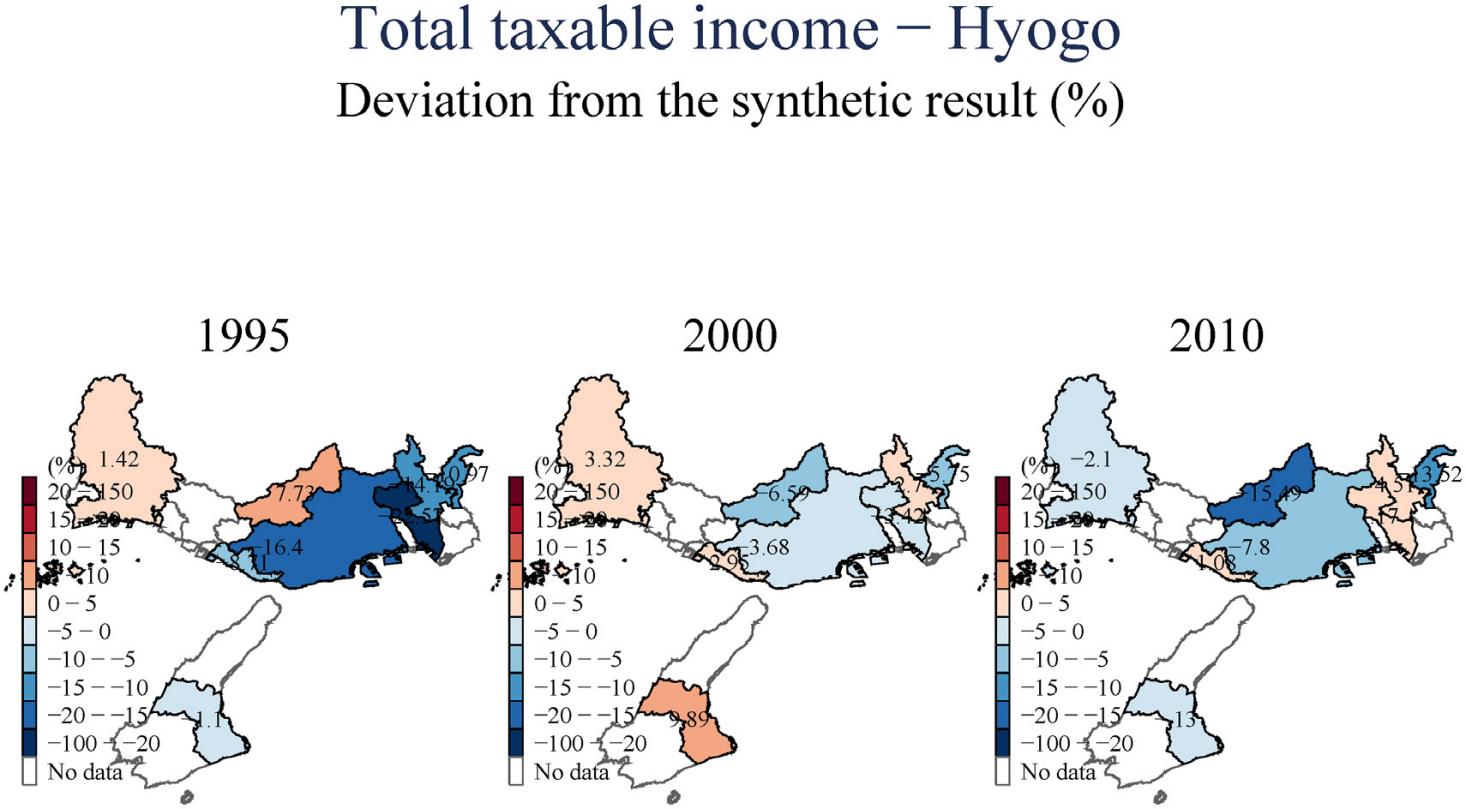
Total Taxable Income—Hyogo.

For income, as we can see from (Fig 10), Kobe City partially bounced back after the earthquake, but there still appears to be a permanent loss in income. Again, we find intra-regional heterogeneous variations in income recovery. While the areas East of Kobe seem to gain in long run, other parts closer to central Kobe lost substantial amount of income, suggesting once more that the proximity to Osaka as a new provider of employment and income may be a driver of the (partial) economic recovery in Kobe’s Eastern region.

**Fig 11:**
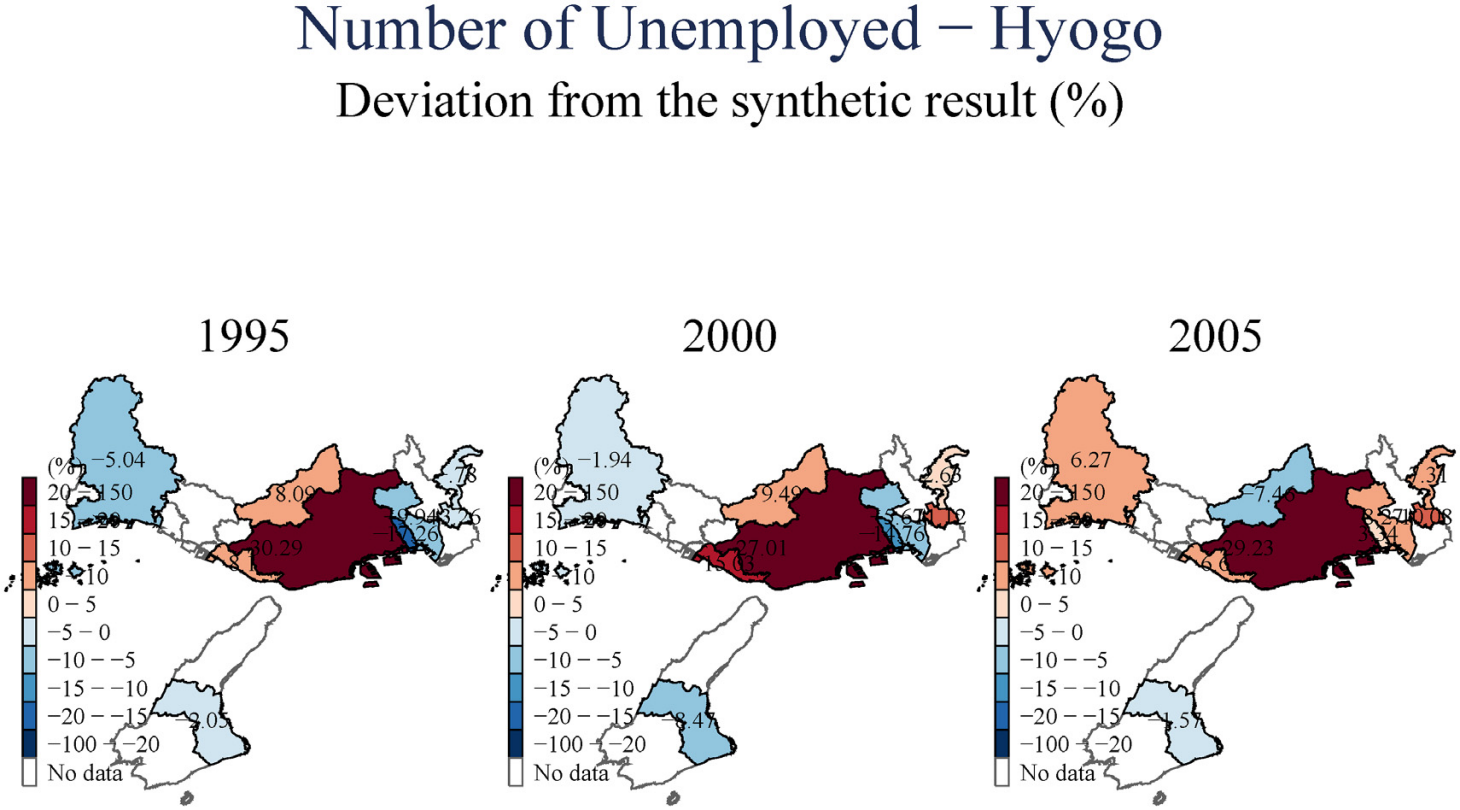
Number of Unemployed—Hyogo.

**Fig 12:**
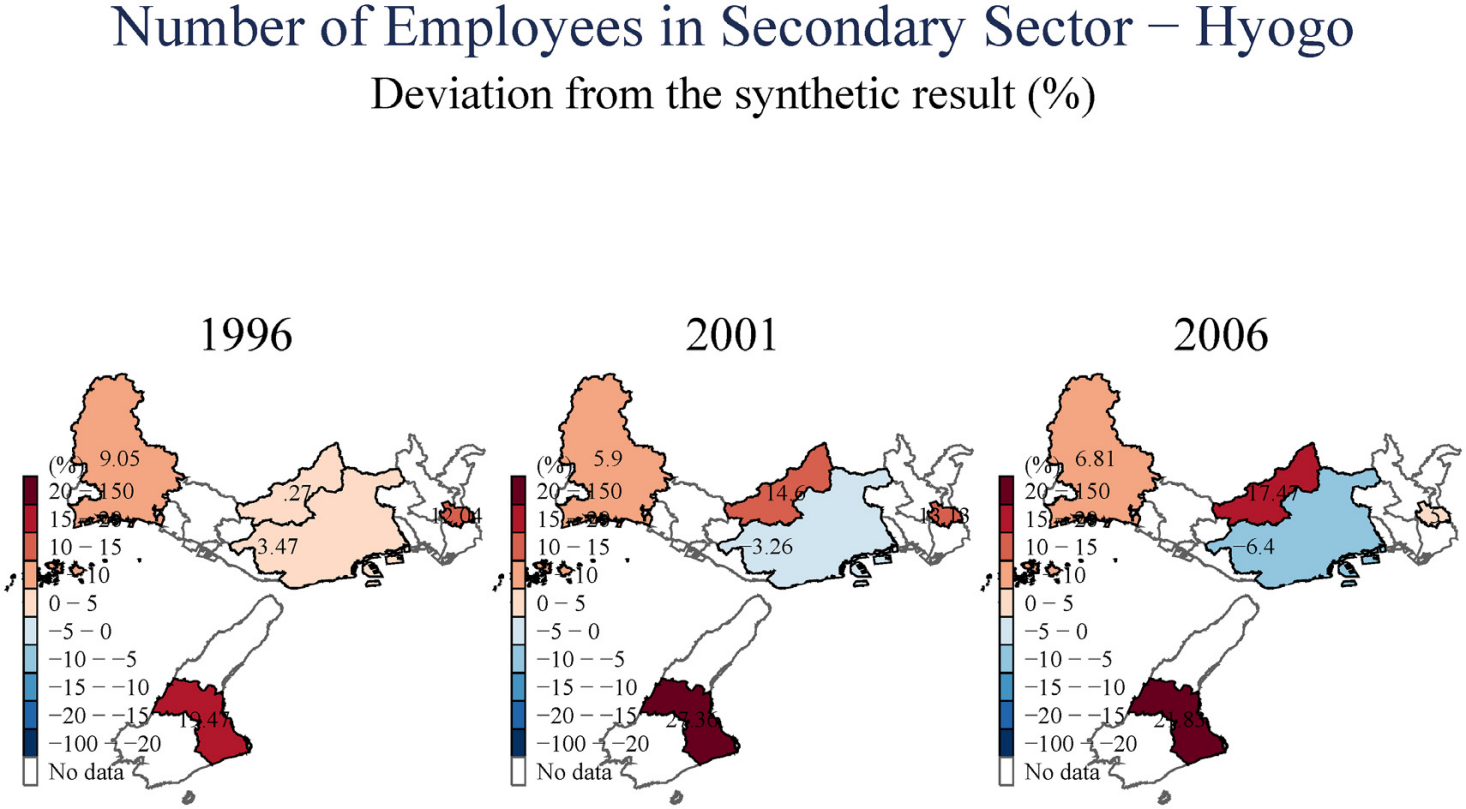
Number of Employees in Secondary Sector—Hyogo.

**Fig 13:**
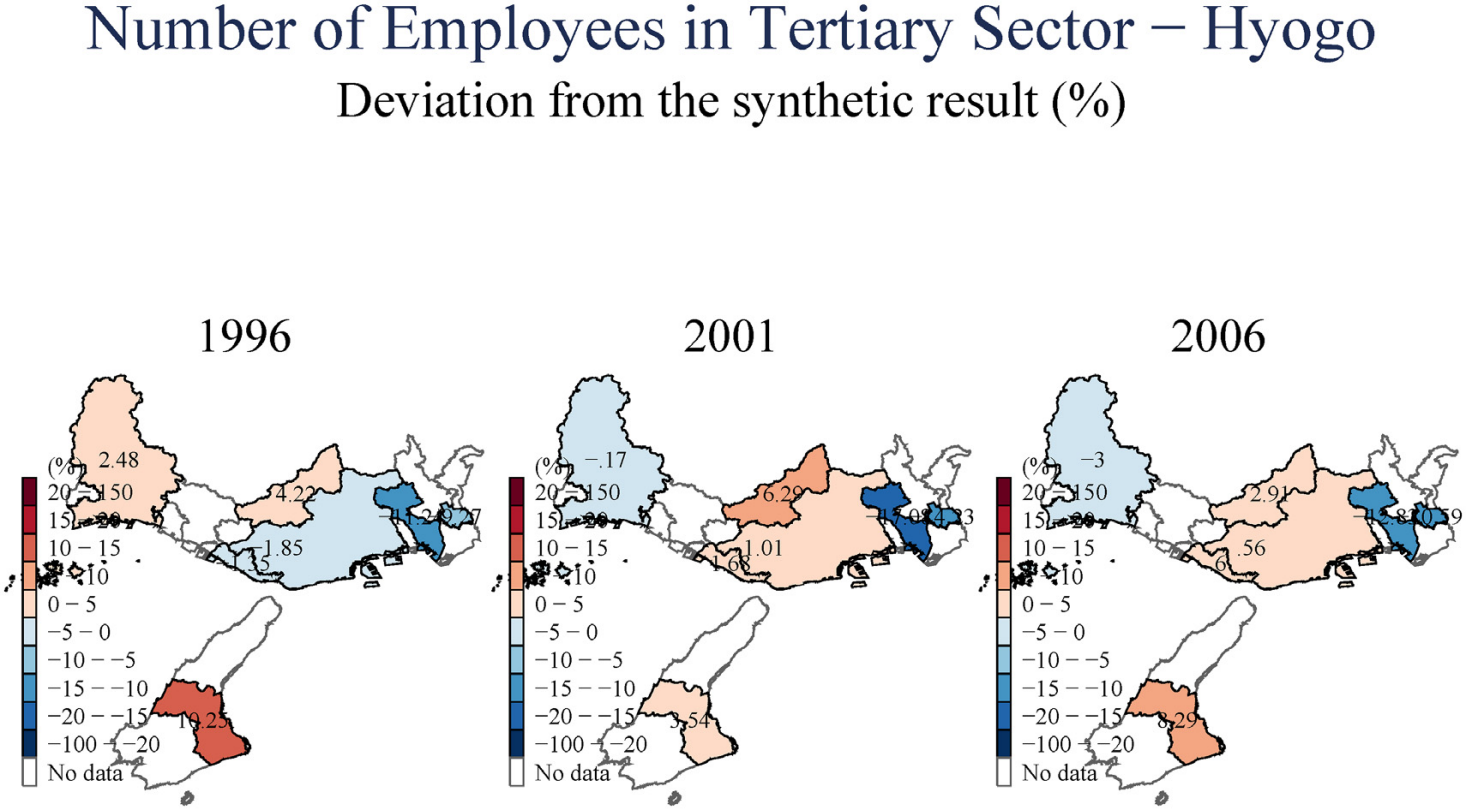
Number of Employees in Tertiary Sector—Hyogo.

We next study aggregate unemployment (Fig 11), and then employment in the secondary (manufacturing) and tertiary (services) sectors in (Figs 12 and 13), respectively. Equivalent analysis of the number of businesses in the secondary and tertiary sectors is available in S1 Fig. The evidence on aggregate unemployment is quite clear. Unemployment increased, both in the short- and in the long-term, and both in Kobe City itself, and in the peripheral towns. Remarkably, the evidence seems to suggest a stronger adverse impact in the long-term (15 years after the earthquake).

The secondary (manufacturing) sector in Kobe City declined both in the short- and long-terms; this decline is observable in both the number of secondary-sector businesses operating in the city, and the level of employment in this sector. The spatial distribution is quite different for the tertiary sector (services). As before, we observe a short-term decline for Kobe City, its wards, and the surrounding towns in both number of operating businesses and employment. However, once we examine the longer-horizon, 15 years after the earthquake, we observe an increase in the number of tertiary (services) businesses operating, accompanied by a smaller increase in employment when evaluated against employment trends elsewhere in Japan. Essentially, it appears that Kobe City experienced a shift from secondary to tertiary employment. This shift may explain the declines in aggregate total taxable income, and as the wages in service sector employment are typically lower than in the industrial/manufacturing sector.

## Conclusions, Caveats, and Future Considerations

The three central empirical regularities that emerged from our synthetic control analysis are: First, incomes and, to a lesser extent, the population of Kobe City have both decreased. This effect of the earthquake lasted for over fifteen years, indicating a significant permanent negative impact. Such a negative impact can be found especially in the central area (e.g., Chuo, Hyogo, and Nagata wards), which is closest to the epicenter of the earthquake. These population shifts to outside of the central area were also identified by [5]. In addition to further confirming these earlier results, we also document the difference between the dynamics for daytime and nighttime populations. This suggests a residential move to the East of Kobe, a shift in employment from Kobe to Osaka, and an increase in the population of the elderly. All these observed patterns are potentially very important if we are to understand the policy implications of these transitions. We further document rigorously that this decline in the central areas also involved a shift from manufacturing to services, a shift that goes beyond the general de-industrialization and shift to services that has been enfolding in Japan and in cities similar to Kobe in the last two decades (as our counterfactuals account for those general trends).

Second, the surrounding areas, in particular East of Kobe (e.g., Nishinomiya city), experienced positive permanent impacts in terms of total taxable income after facing short-run negative effects in the immediate aftermath of the earthquake. Previous research has not examined incomes, but we note that this positive impact did not result in increased employment in this region. Rather, this region’s increased population is mostly employed in nearby Osaka (further to the East).

Third, the peripheral areas in Hyogo seem to have been insulated from the large direct and indirect impacts of the earthquake. This further confirms previous findings from other case studies that disasters are localized events, and do not entail larger and more permanent impacts further afield, as noted by [14] and [19], for example. It also further highlights the need to focus on the immediate locale, and not mis-interpret the economic state of the larger region as an indicator of what is occurring in the immediate location of the disaster (as we have seen frequently done).

Some important caveats for this work are worth mentioning. (S1 Fig) includes a variety of placebo tests we conducted to establish the statistical significance of our results (as distinct from their real economic significance). We have not been able to always do that, so in some cases our results are more tentative. The general malaise afflicting the Japanese economy in the 1990s (and later into the 2000s) is accounted for by the synthetic control methodology, and that is probably the most important advantage of this method. However, if that malaise was at least partially made worse by the events in Kobe in 1995, then our estimates are understated. Given previous research on other case studies, we find that unlikely.

Another potential drawback of our description is that our examination of labour markets focuses only on employees, even though this is probably the most important mechanism in the changes we describe. A potentially fruitful research agenda is to identify more specifically the changes from the employers’ perspectives. [29], for example, attempts to identify the incentives and circumstances that guided employers’ decisions to exit the region. This research agenda, however, is still not sufficiently conclusive in our view.

Once the spatial and dynamic responses of each region, city and ward has been described, the next research task is to identify the policy determinants of these differing trajectories, and to further investigate whether possible policy shifts could have led to more favorable outcomes. This, unfortunately, is outside the scope of this paper. Instead of relying on the ward-level dataset we used, other alternative sources of information and methodology may yield additional insights about the process of recovery (or lack thereof) in Kobe post-1995, and especially on its policy determinants (e.g. [22]).

Finally, it is worth repeating that we believe that the long-term or permanent costs of disasters may be significant as they impose large permanent impacts on human wellbeing in affected regions [33, 34]. Our results here suggest that one such large catastrophic shock, the 1995 Kobe earthquake, did indeed impose long-term quantifiable costs on the affected region. These costs are typically not clearly identified and are thus not considered when assessing the benefits from disaster risk reduction and mitigation policies.

This failure leads to under-investment in reducing risks from disasters (the direct costs), and in trying to mitigate their impacts (the indirect long-term ones). Maybe more importantly and less obviously, we also believe that this failure to recognize the long-term permanent impacts leads to complacency during the post-disaster recovery process itself. Policymakers and the public believe that recovery will inevitably be achieved, and are thus mostly making policy and electoral decisions based on short-term considerations rather than in an attempt to guide this long-term process on a more successful and improved trajectory.

A different concern and motivation for our research agenda is the well-documented increasing economic costs of natural disasters [35], even if there is uncertainty regarding the reasons for this trend. The socio-economic dynamics we investigated here are bound to become more important in the future, even if some of the more dire predictions regarding the impact of climate change on extreme climatic events do not materialize [36]. Our publics, our governments, our international organizations, and the international agreements and covenants we agree on (most relevant is the recently agreed UN Sendai Framework for Disaster Risk Reduction) must take into account these long-term permanent impacts in guiding future actions. Awareness of these potentially long-term adverse impacts should lead, ceteris paribus, to more concentrated and effective investment in disaster risk prevention and reduction, and to better policy-making in the aftermath of catastrophes.

## Supporting Information

S1 Fig Additional Variables and Placebo Results. (PDF)
