# Supplementary material for: The Long-Run Socio-Economic Consequences of a Large Disaster: The 1995 Earthquake in Kobe
Source: PLoS One. 2015 Oct 1;10(10):e0138714. doi: 10.1371/journal.pone.0138714 (PMC4591010; doi:10.1371/journal.pone.0138714)
Supplement: S1 Fig — (PDF) [file pone.0138714.s001.pdf]

The Long-Run Socio-Economic Consequences of a Large Disaster: The 1995 Earthquake in Kobe

ONLINE APPENDIX

William duPont IV (*College of Saint Benedict|Saint John's University*), Ilan Noy (*Victoria University of Wellington*), Yoko Okuyama (*Yale University), and* Yasuyuki Sawada (*University of Tokyo and RIETI).*

Appendix posted at:
https://sites.google.com/site/noyeconomics/research/natural-disasters

Abstract

We quantify the 'permanent' socio-economic impacts of the Great Hanshin-Awaji (Kobe) earthquake in 1995 by employing a large-scale panel dataset of 1,719 cities, towns, and wards from Japan over three decades. In order to estimate the counterfactual—i.e., the Kobe economy without the earthquake—we use the synthetic control method. Three important empirical patterns emerge: First, the population size and especially the average income level in Kobe have been lower than the counterfactual level without the earthquake for over fifteen years, indicating a permanent negative effect of the earthquake. Such a negative impact can be found especially in the central areas that are closer to the epicenter. Second, the surrounding areas experienced some positive permanent impacts in spite of short-run negative effects of the earthquake. Much of this is associated with movement of people to East Kobe, and consequent movement of jobs to the metropolitan center of Osaka, that is located immediately to the East of Kobe. Third, the furthest areas in the vicinity of Kobe seem to have been insulated from the large direct and indirect impacts of the earthquake.

**Contents**		**Page No.**
Figure 1.	A1001 The total number of the population	3
Figure 2.	A1076 More than 65 year-old total population	4
Figure 3.	C1352 Population in register – Total	5
Figure 4.	C1353 Population in register – Male	6
Figure 5.	C1354 Population in register – Female	7
Figure 6.	C1495 Day time population	8
Figure 7.	Impact on Taxpayer Income in Kobe City	9
Figure 8.	Impact on Taxpayer Income in Nishinomiya City	9
Figure 9.	Impact on Taxpayer Income in Yokohama City	10
Figure 10.	C1632 Taxable income	10
Figure 11.	C1633 Number of taxpayers	11
Figure 12.	C1690 Number of the secondary industry businesses	12
Figure 13.	C1691 Number of tertiary sector businesses	13
Figure 14.	C1724 Number of employees in the secondary sector	14
Figure 15.	C1725 Number of employees in the tertiary sector	15
Figure 16.	F2655 Number of Unemployed in Kobe	16
Figure 17.	F2655 Number of Unemployed	17
Figure 18.	Placebos for Registered Population (all cities)	18-28
Figure 19.	Placebos for Taxable Income	29-39
Table 1.	Kobe Population (A1352) Predictor Means	40-41
Table 2.	Data sources	42-44
Appendix.	Impacts on Each Variable in Each Ward	45-51

Figure 1. A1001 The total number of the population

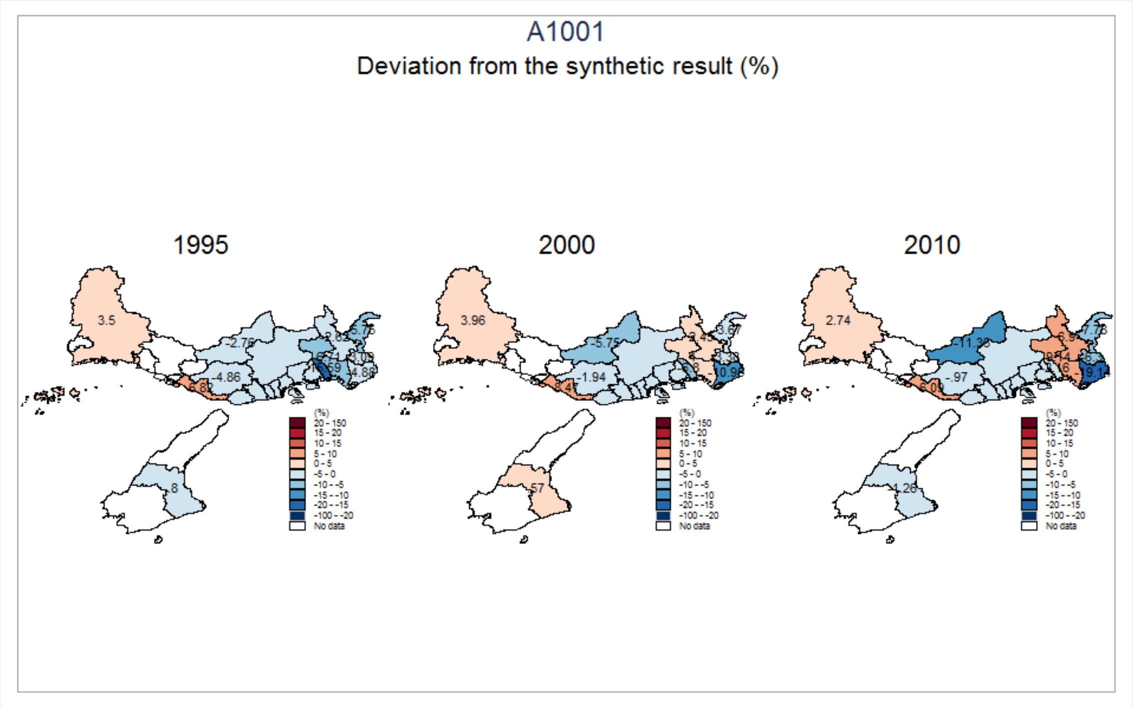

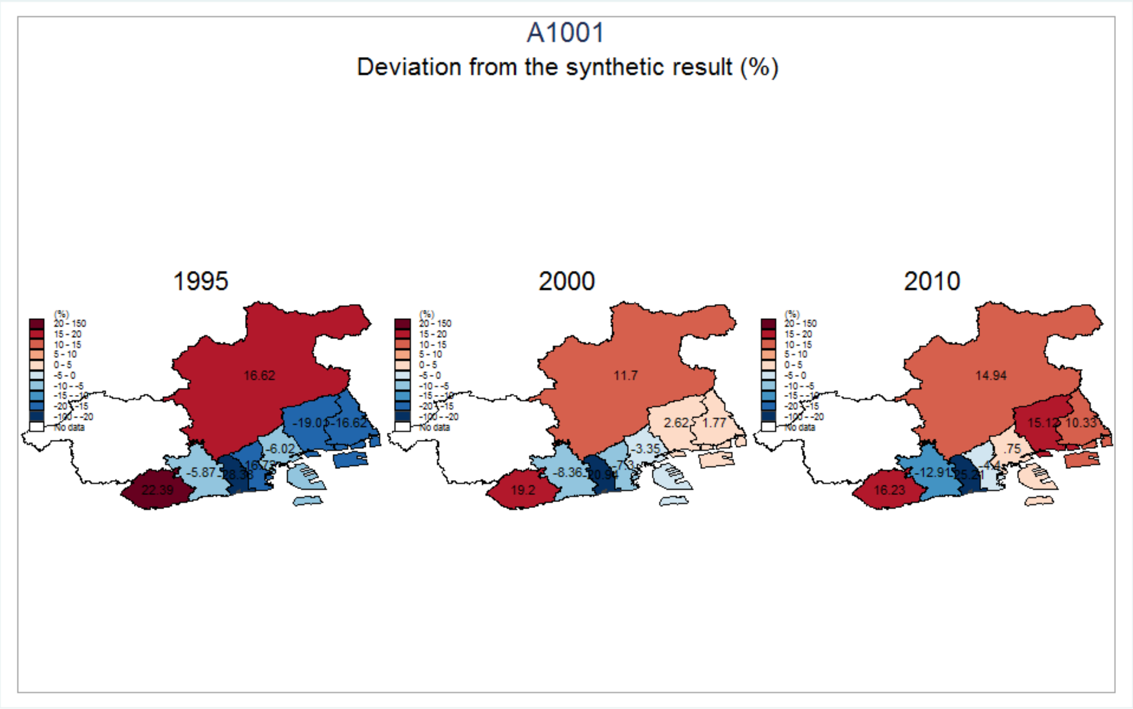

*Cities and wards with synthetic results that sufficiently reproduce the actual values prior to the Hanshin Awaji Earthquake (Jan.1995).

Figure 2. A1076 More than 65 year-old total population

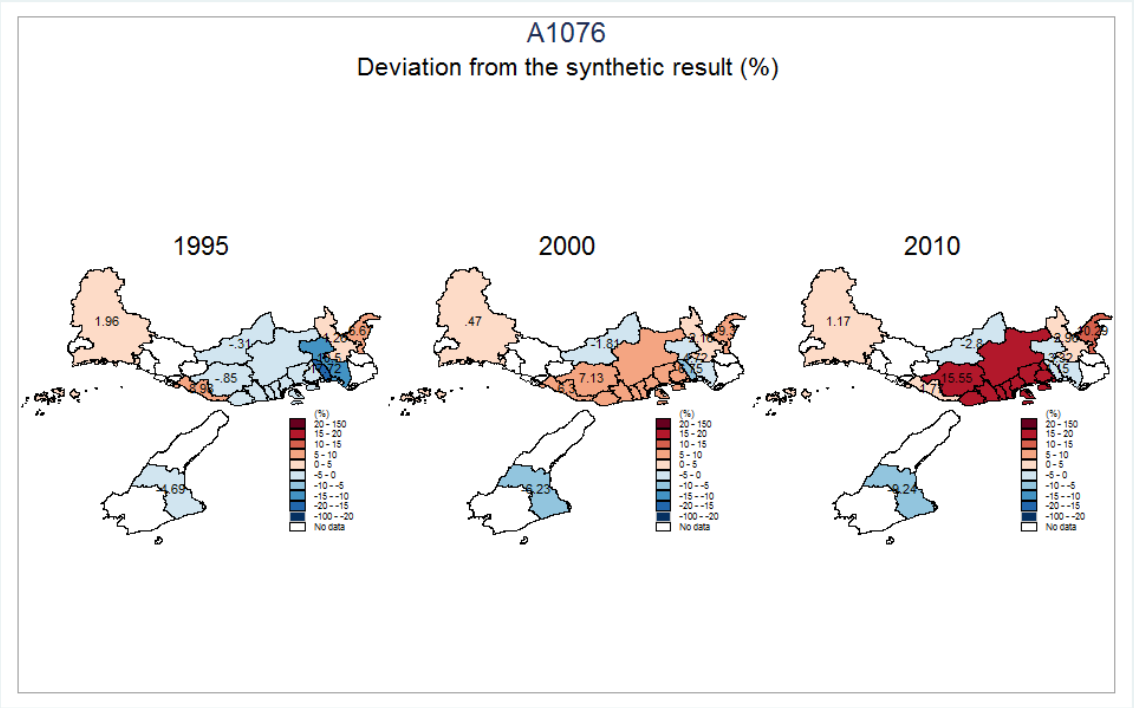

*Cities and wards with synthetic results that sufficiently reproduce the actual values prior to the Hanshin Awaji Earthquake (Jan.1995).

Figure 3. A1352 Population in register – Total

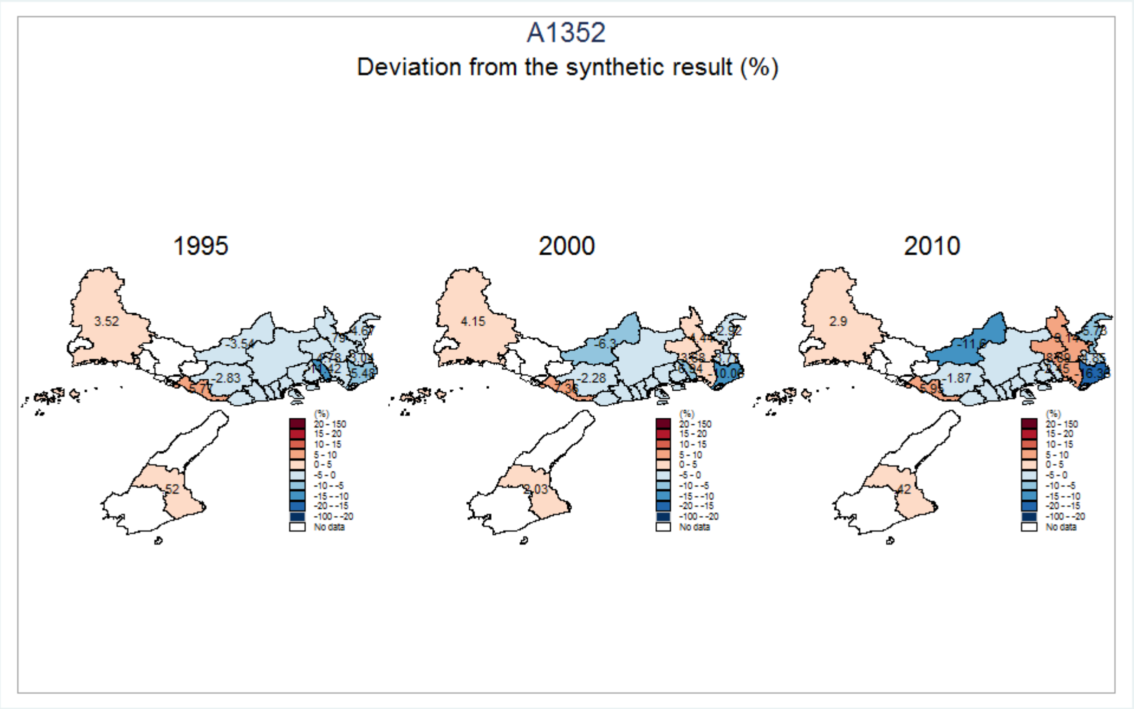

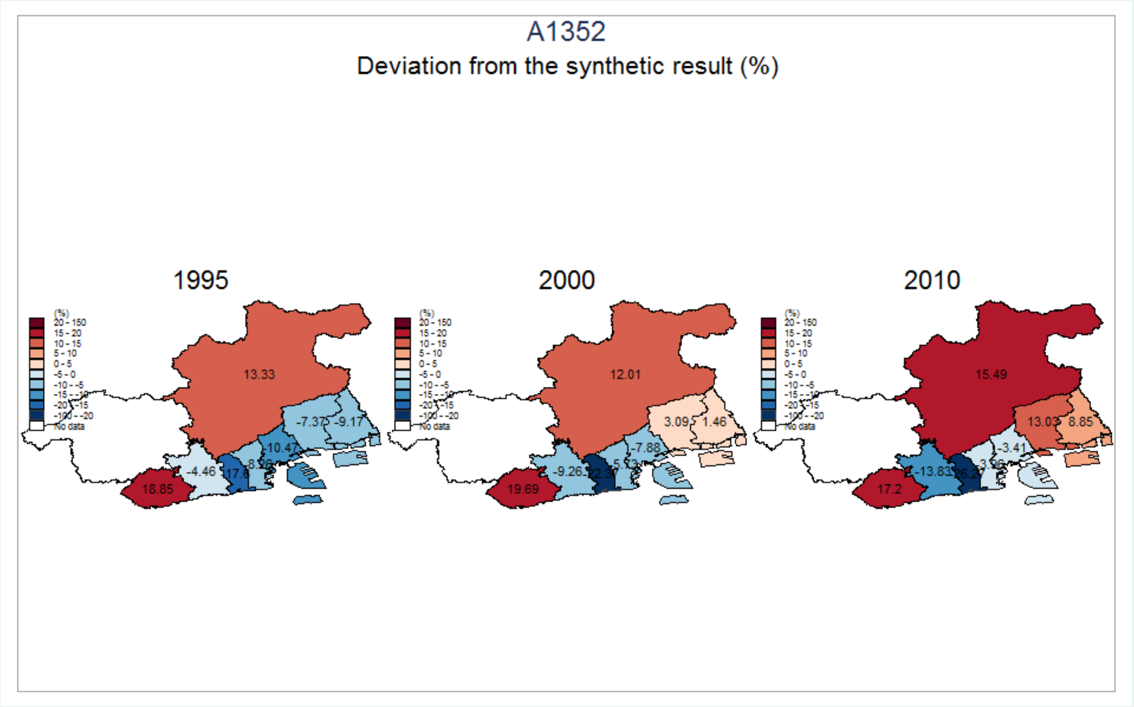

*Cities and wards with synthetic results that sufficiently reproduce the actual values prior to the Hanshin Awaji Earthquake (Jan.1995).

Figure 4. A1353 Population in register – Male

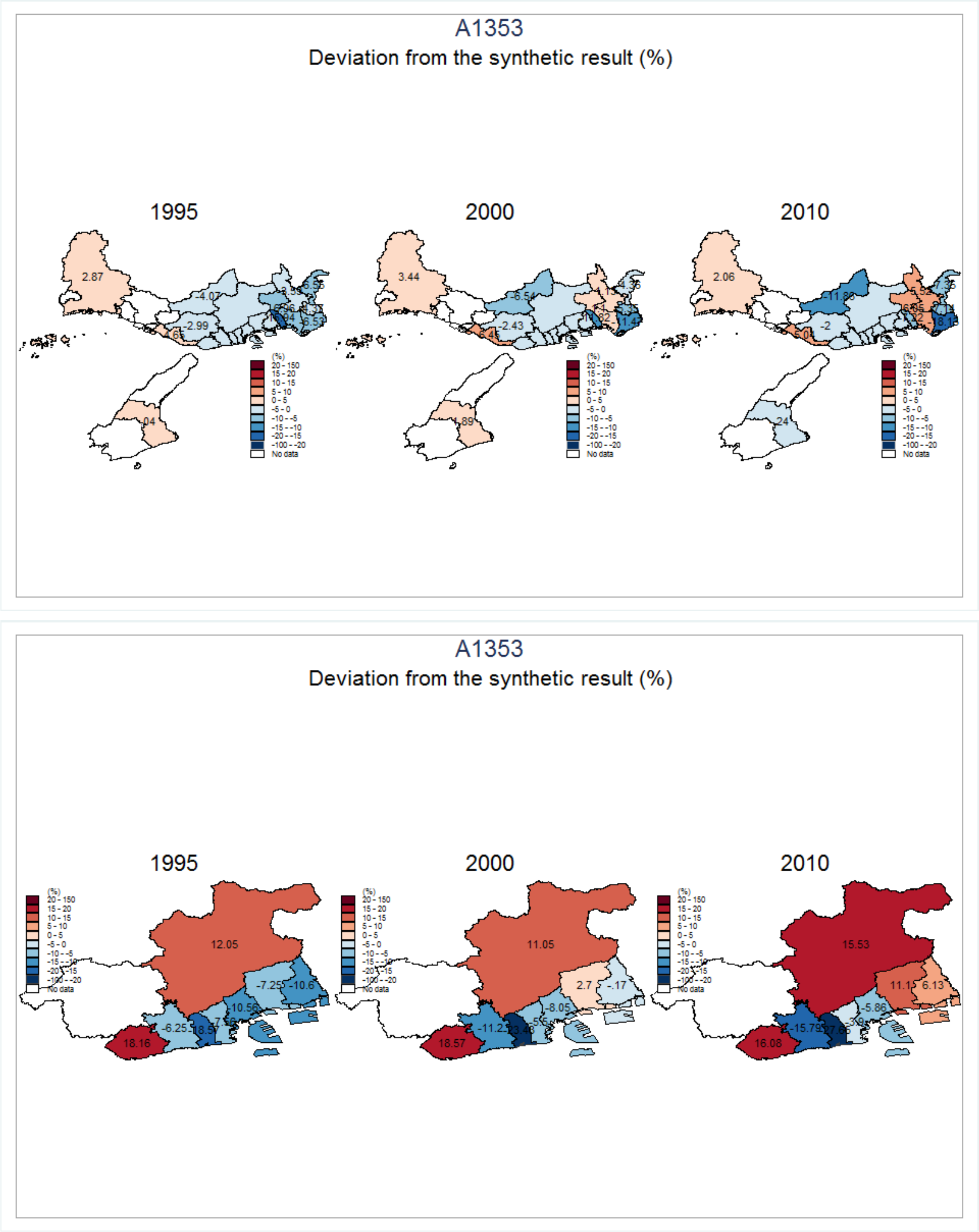

*Cities and wards with synthetic results that sufficiently reproduce the actual values prior to the Hanshin Awaji Earthquake (Jan.1995).

Figure 5. C1354 Population in register – Female

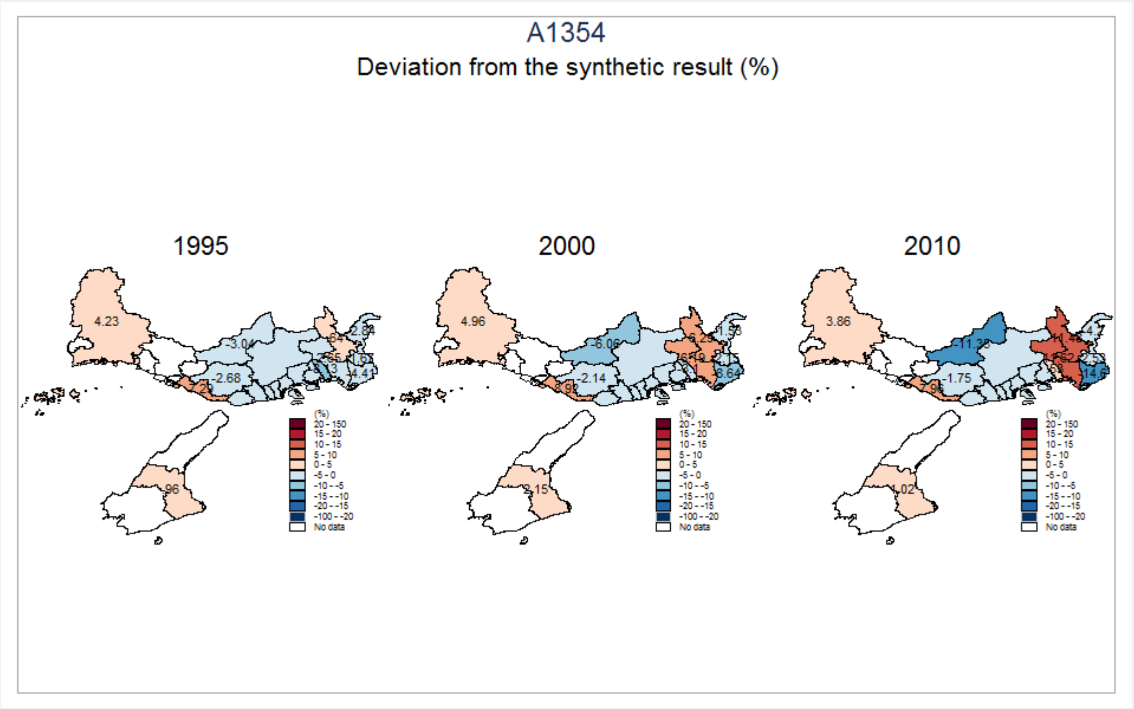

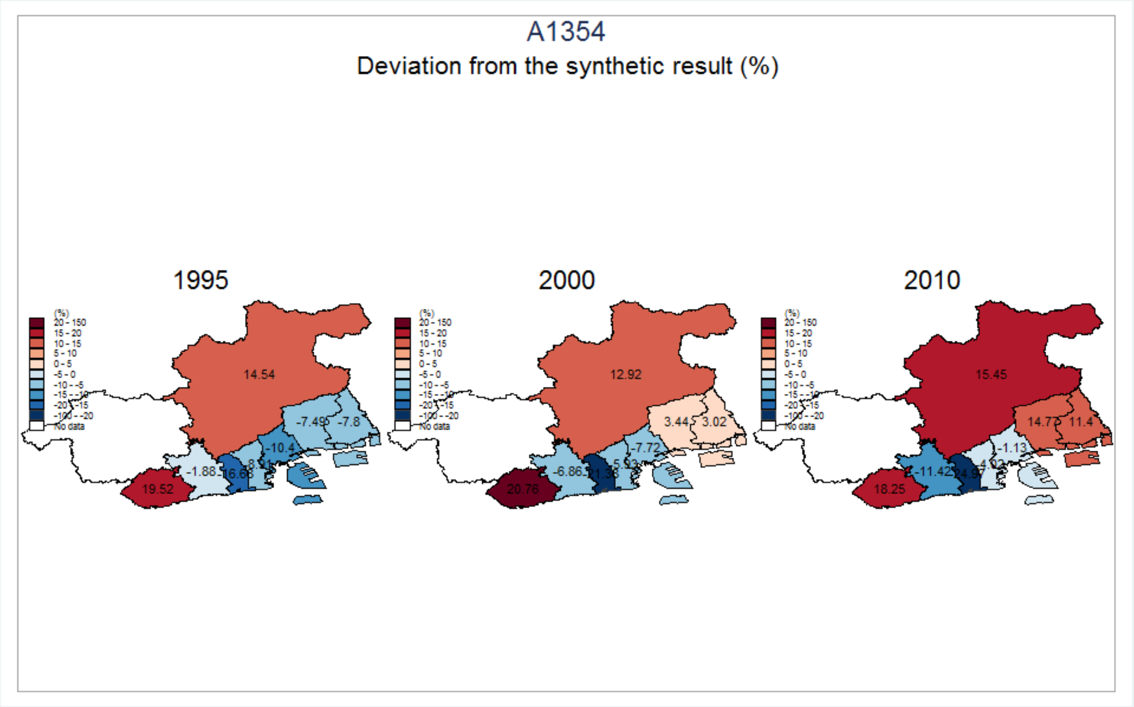

*Cities and wards with synthetic results that sufficiently reproduce the actual values prior to the Hanshin Awaji Earthquake (Jan.1995).

Figure 6. C1495 Day time population

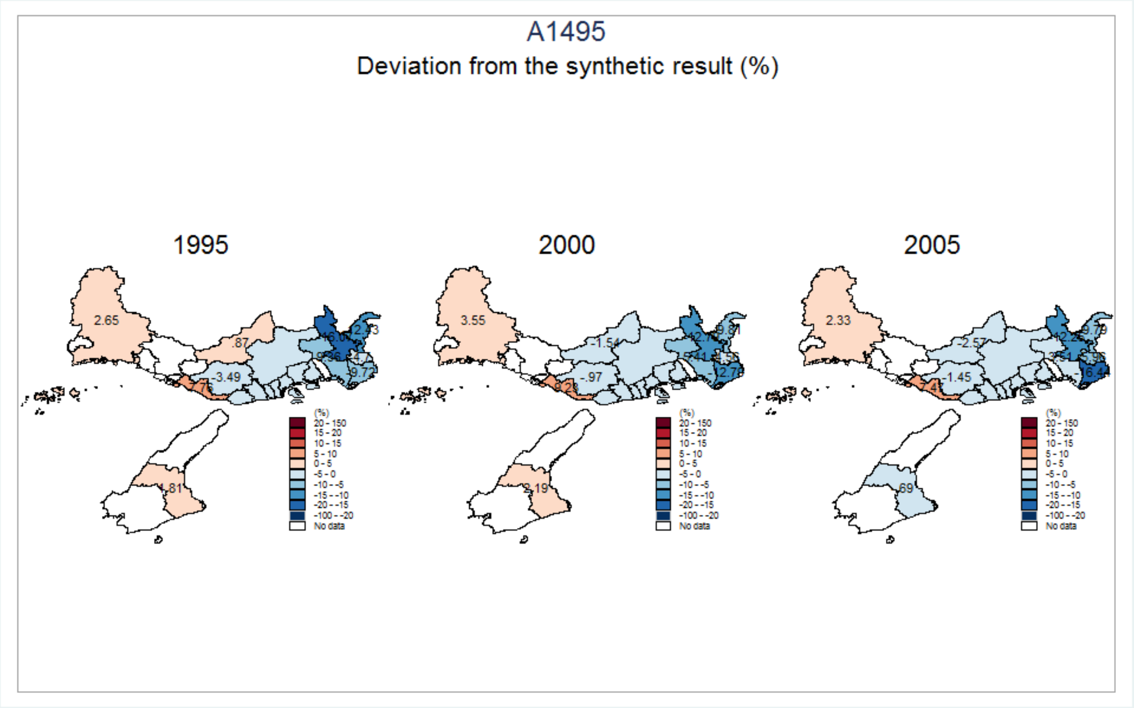

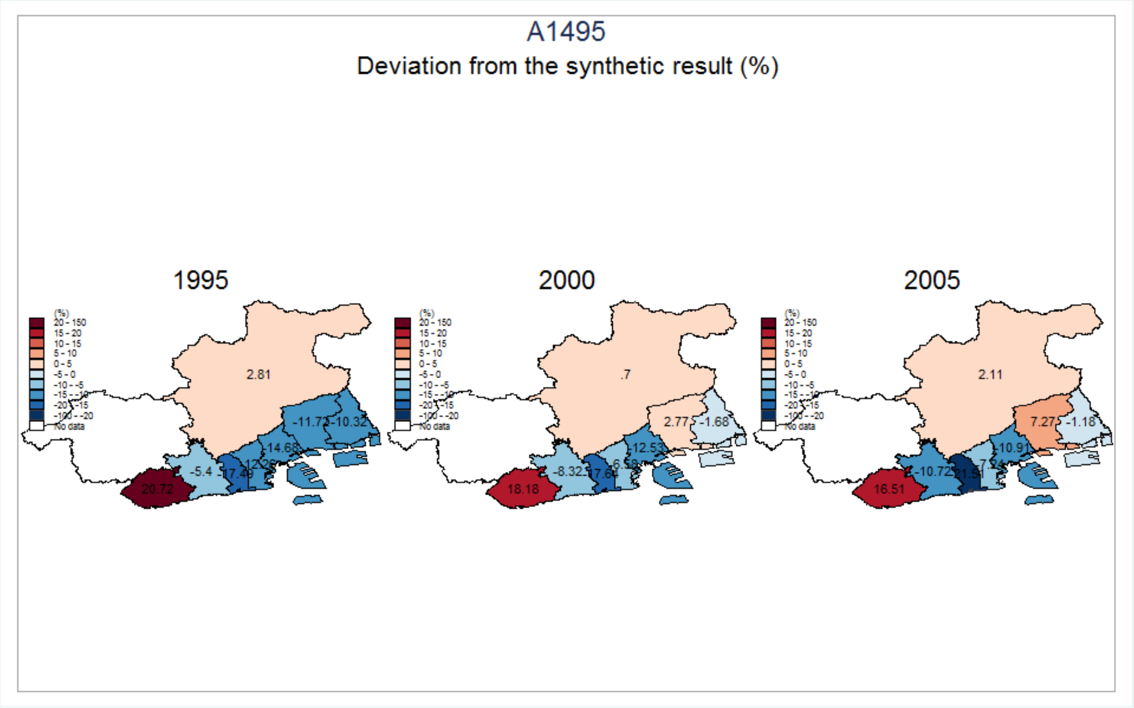

*Cities and wards with synthetic results that sufficiently reproduce the actual values prior to the Hanshin Awaji Earthquake (Jan.1995).

Figure 7. Impact of the Earthquake on Taxpayer Income in Kobe City

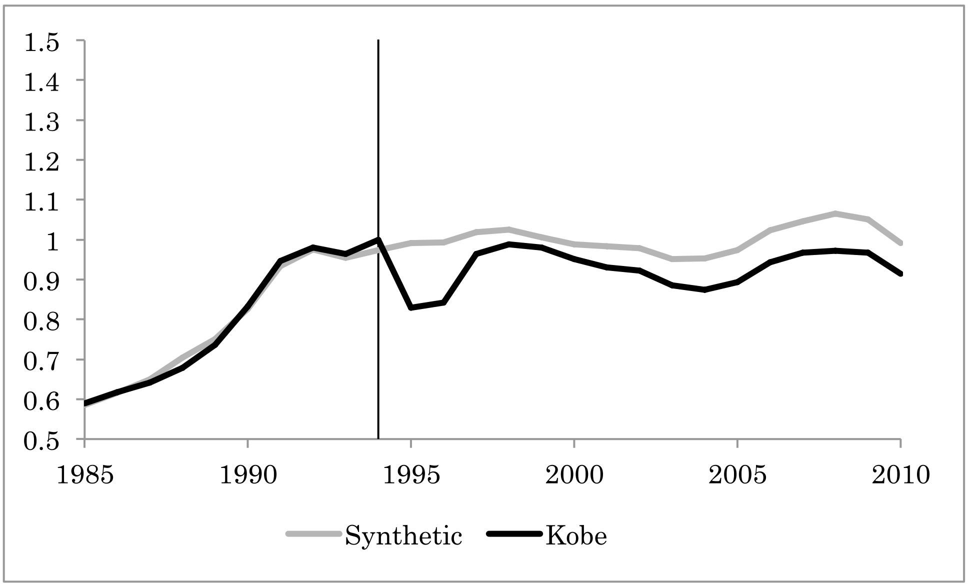

Figure 8. Impact of the Earthquake on Taxpayer Income in Nishinomiya City

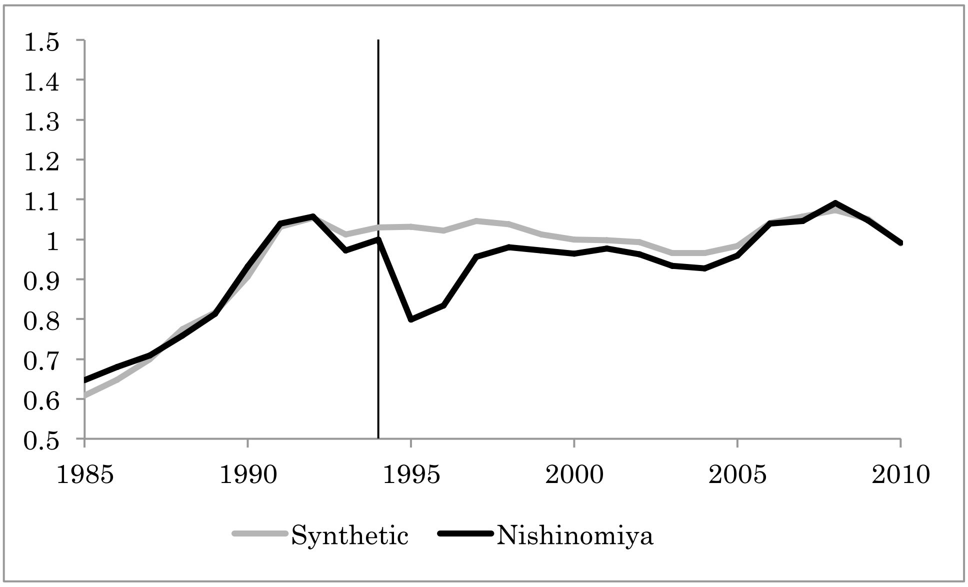

Figure 9. Impact of the Earthquake on Taxpayer Income in Yokohama City

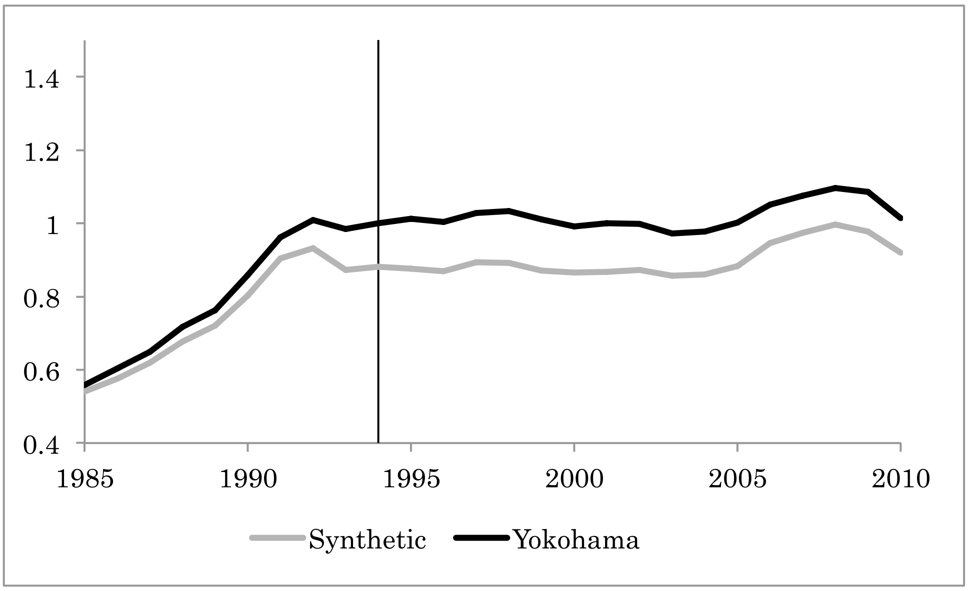

Figure 10. C1632 Taxable income

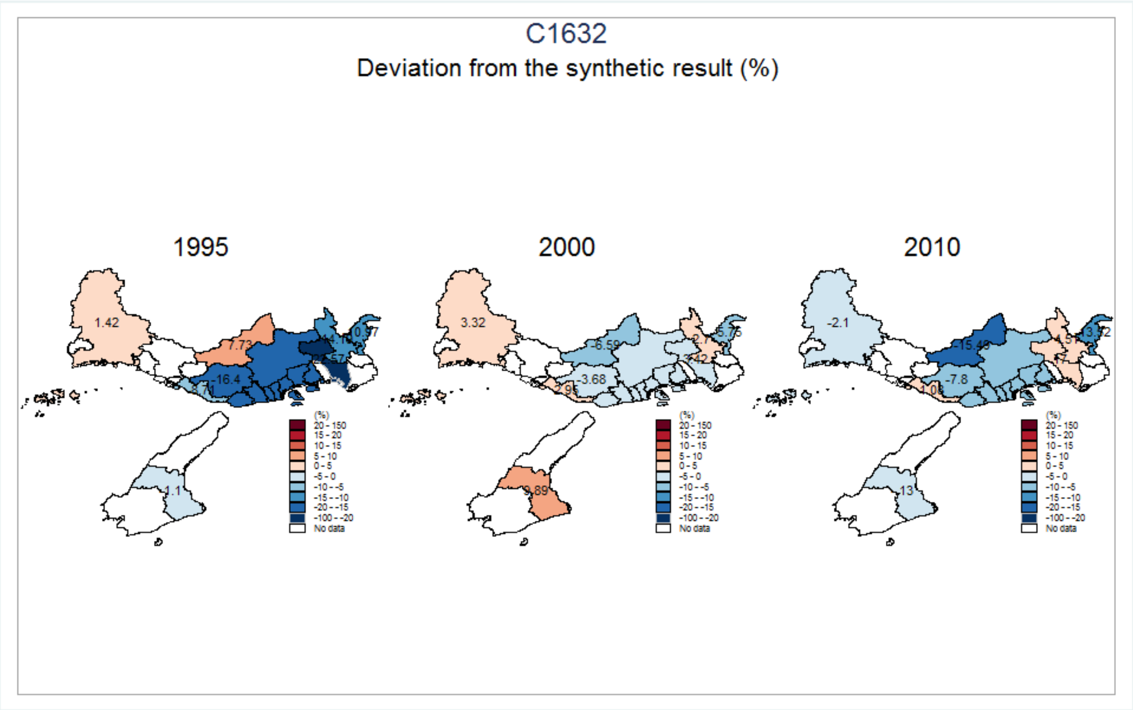

Figure 11. C1633 Number of taxpayers

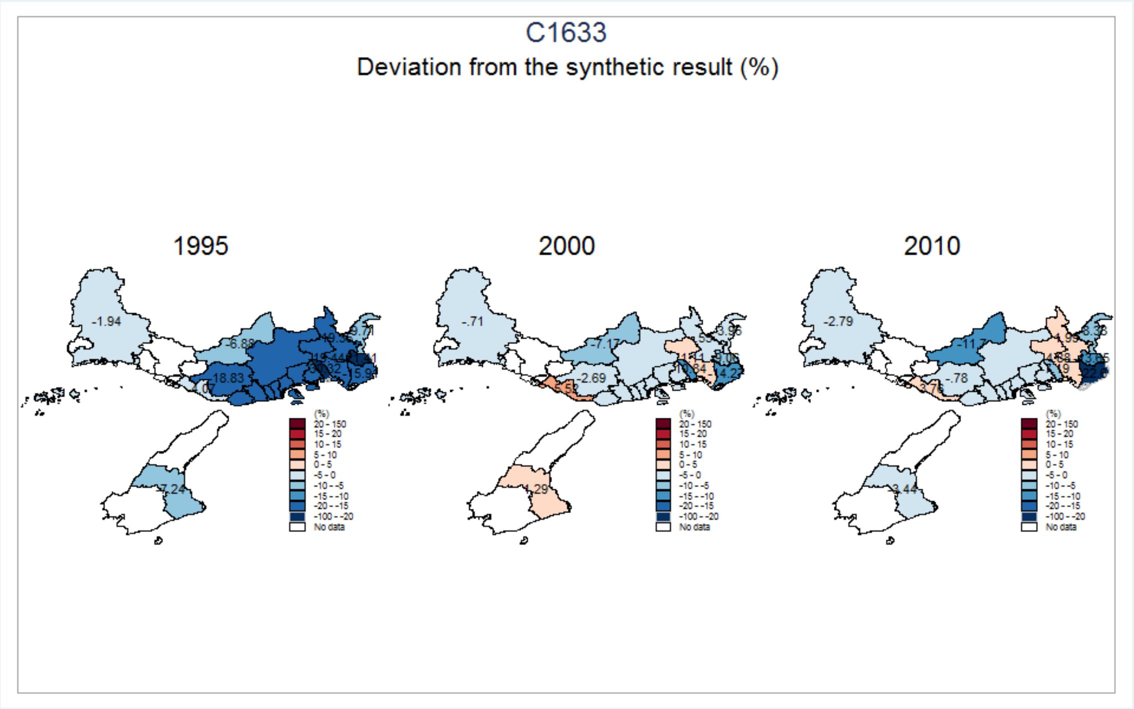

*Cities and wards with synthetic results that sufficiently reproduce the actual values prior to the Hanshin Awaji Earthquake (Jan.1995).

Figure 12. C1690 Number of the secondary industry business

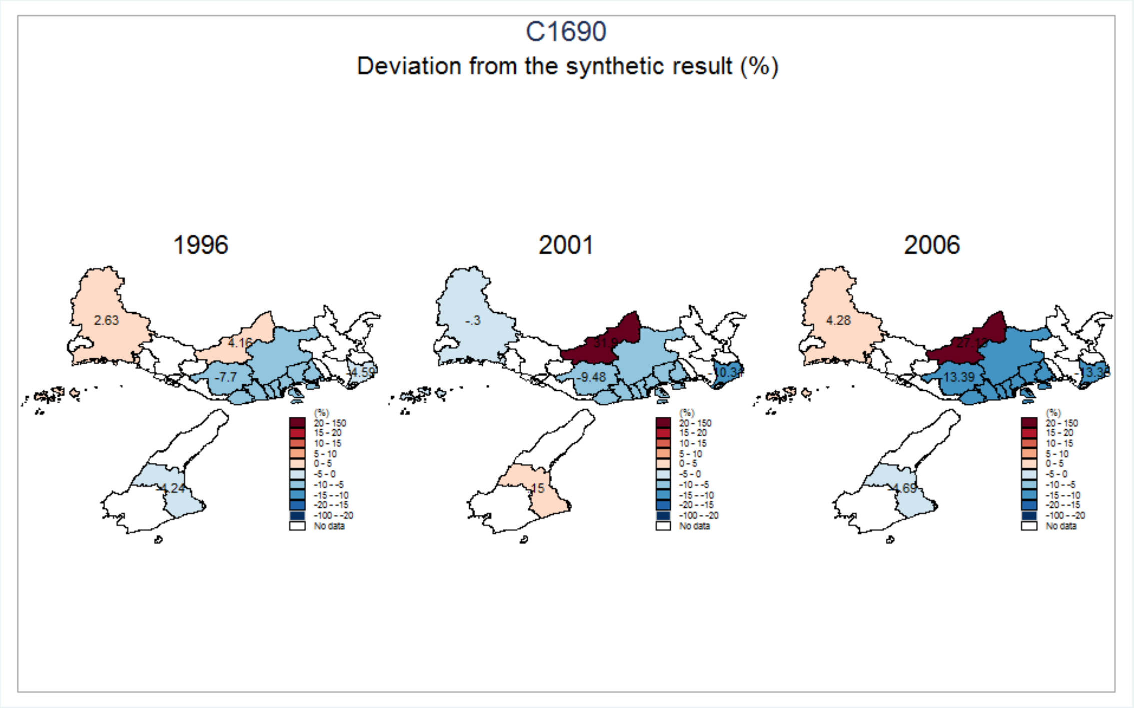

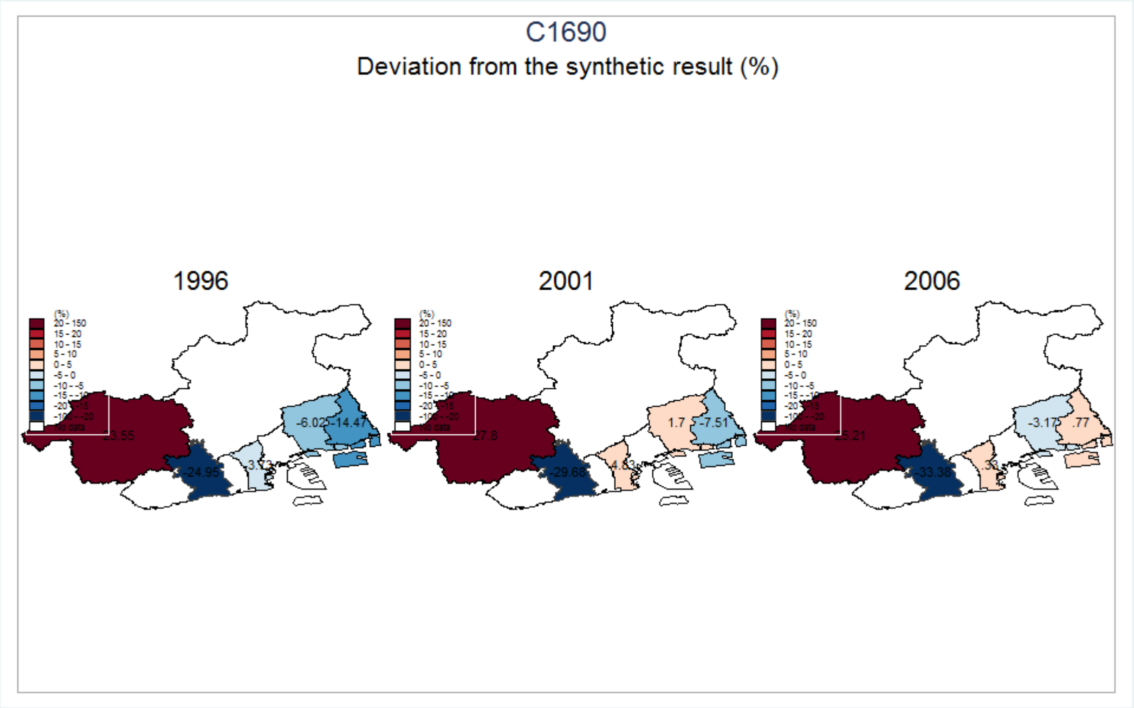

*Cities and wards with synthetic results that sufficiently reproduce the actual values prior to the Hanshin Awaji Earthquake (Jan.1995).

Figure 13. C1691Number of tertiary industry business

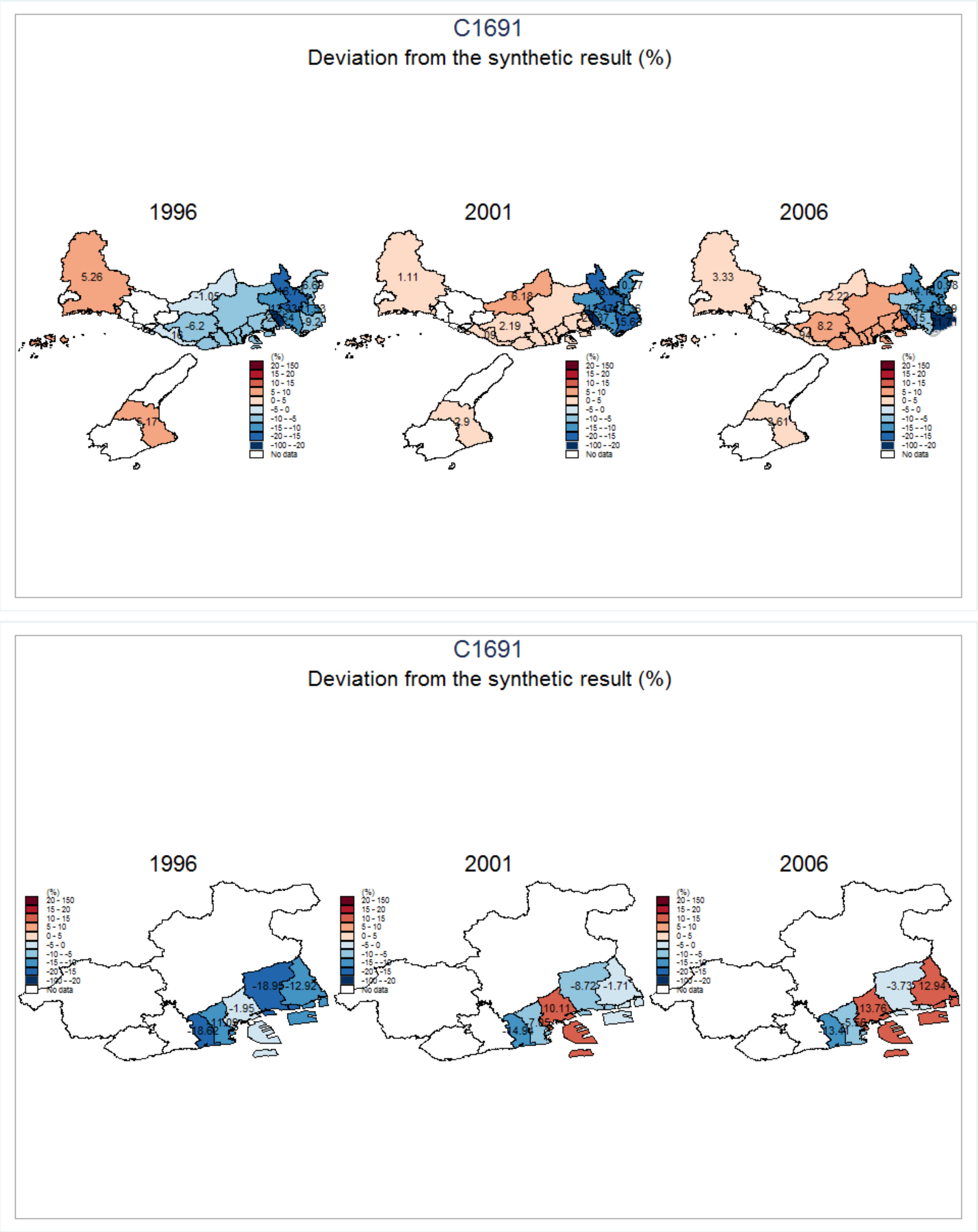

*Cities and wards with synthetic results that sufficiently reproduce the actual values prior to the Hanshin Awaji Earthquake (Jan.1995).

Figure 14. C1724 Number of employees in the secondary industry business

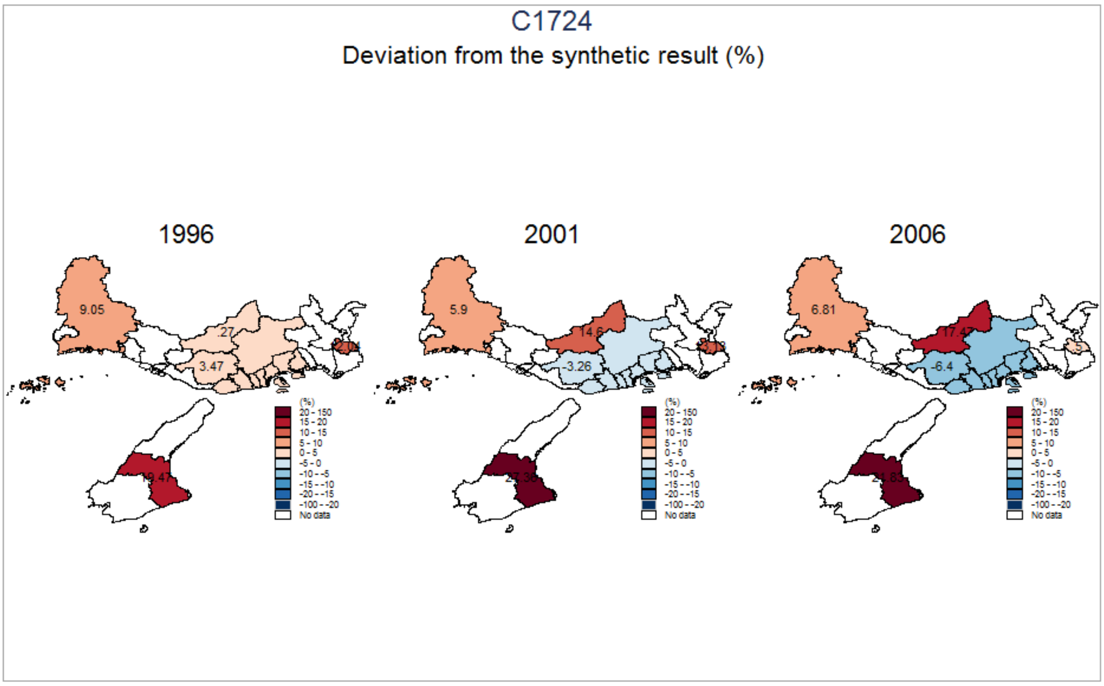

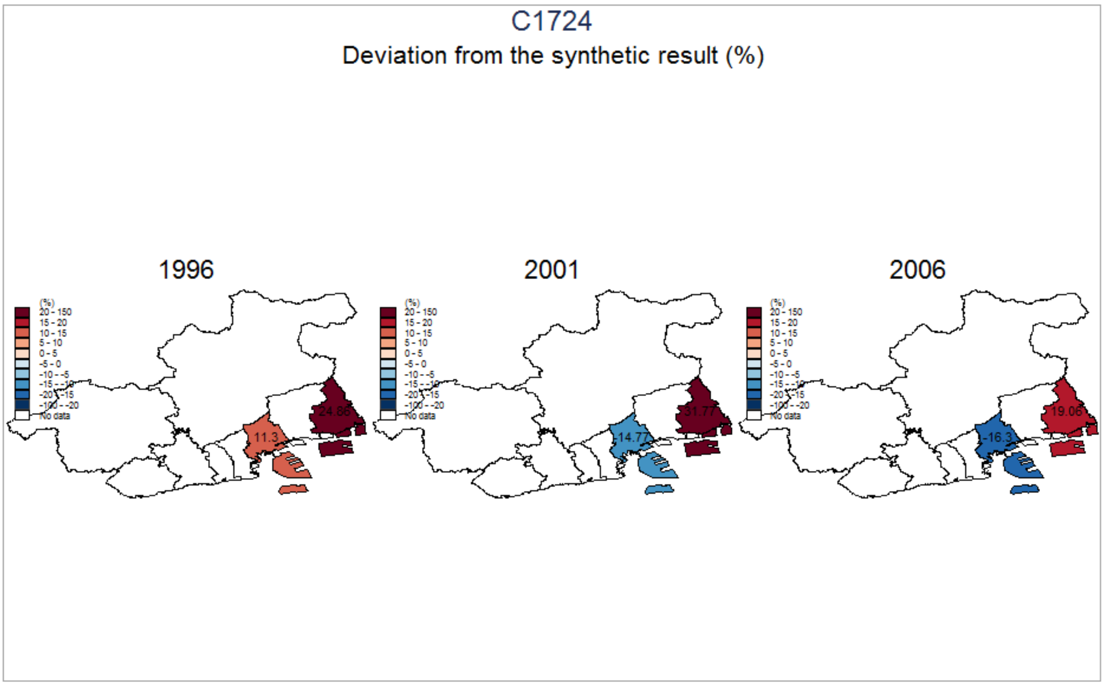

*Cities and wards with synthetic results that sufficiently reproduce the actual values prior to the Hanshin Awaji Earthquake (Jan.1995).

Figure 15. C1725 Number of employees in the tertiary sector

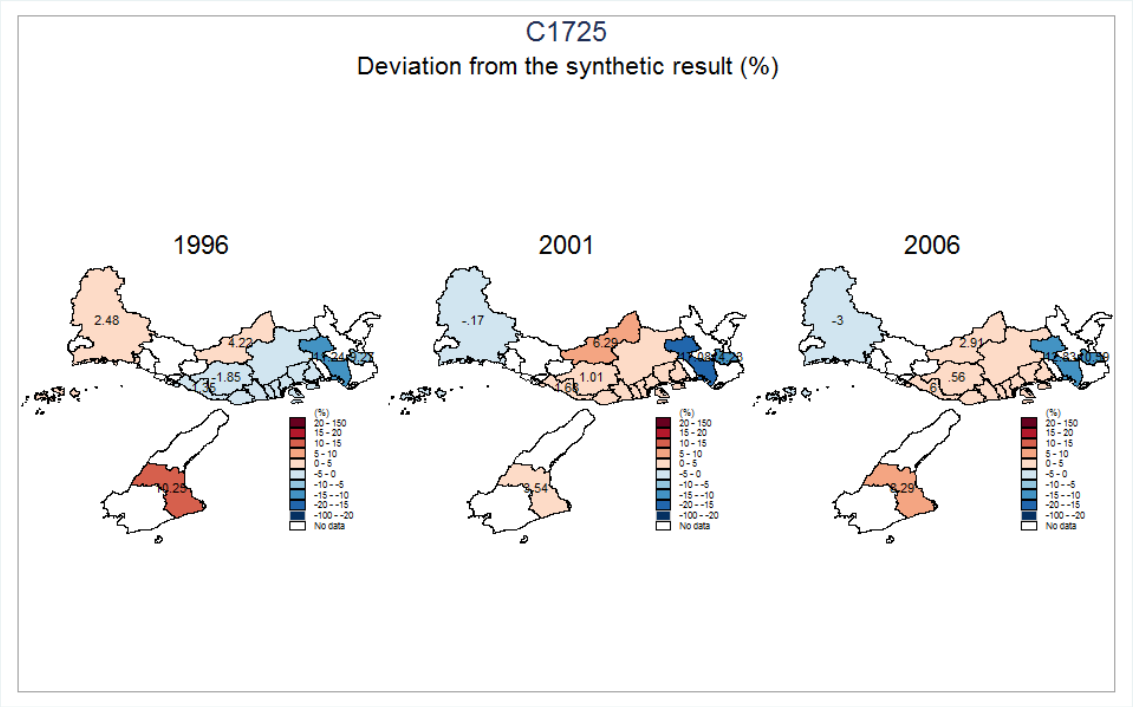

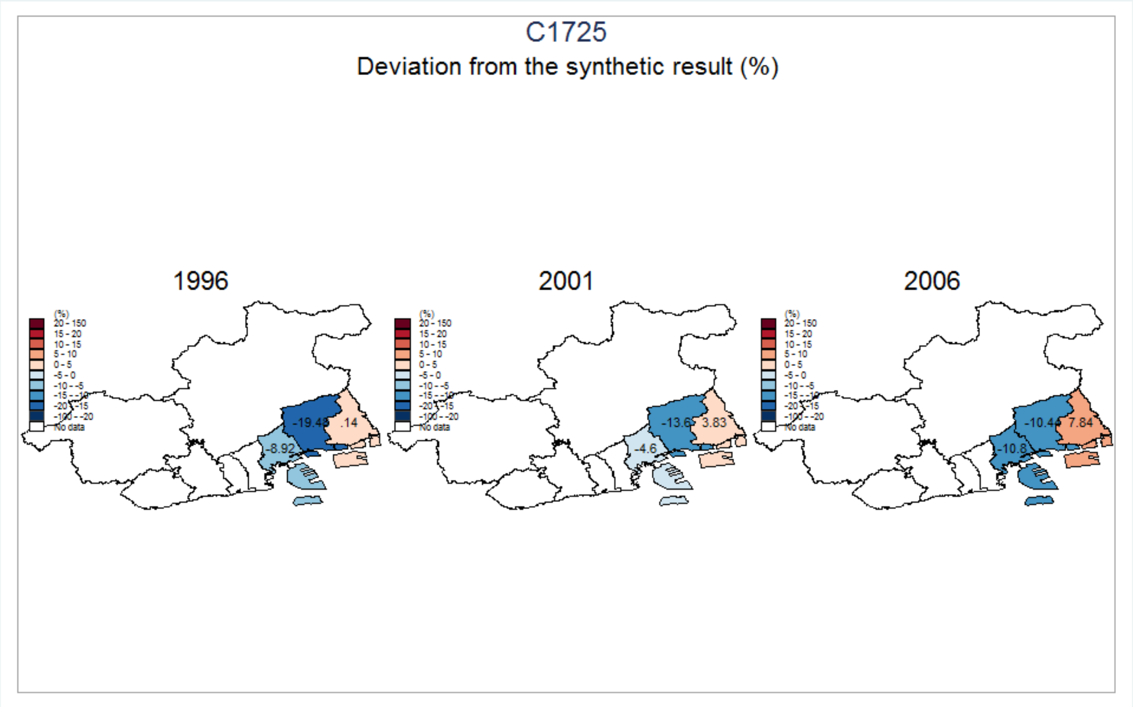

*Cities and wards with synthetic results that sufficiently reproduce the actual values prior to the Hanshin Awaji Earthquake (Jan.1995).

Figure 16. F2655 Number of Unemployed in Kobe

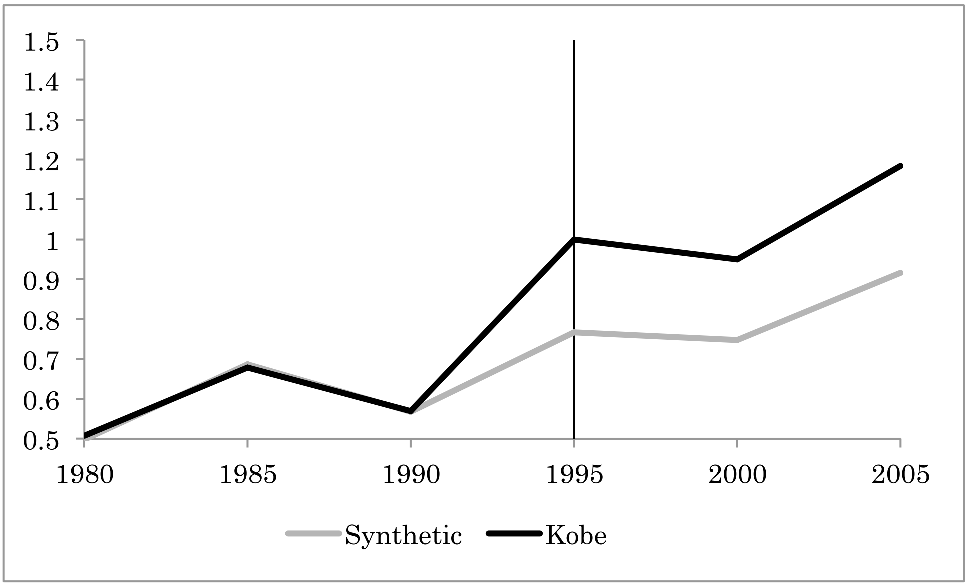

Figure 17. F2655 Number of Unemployed

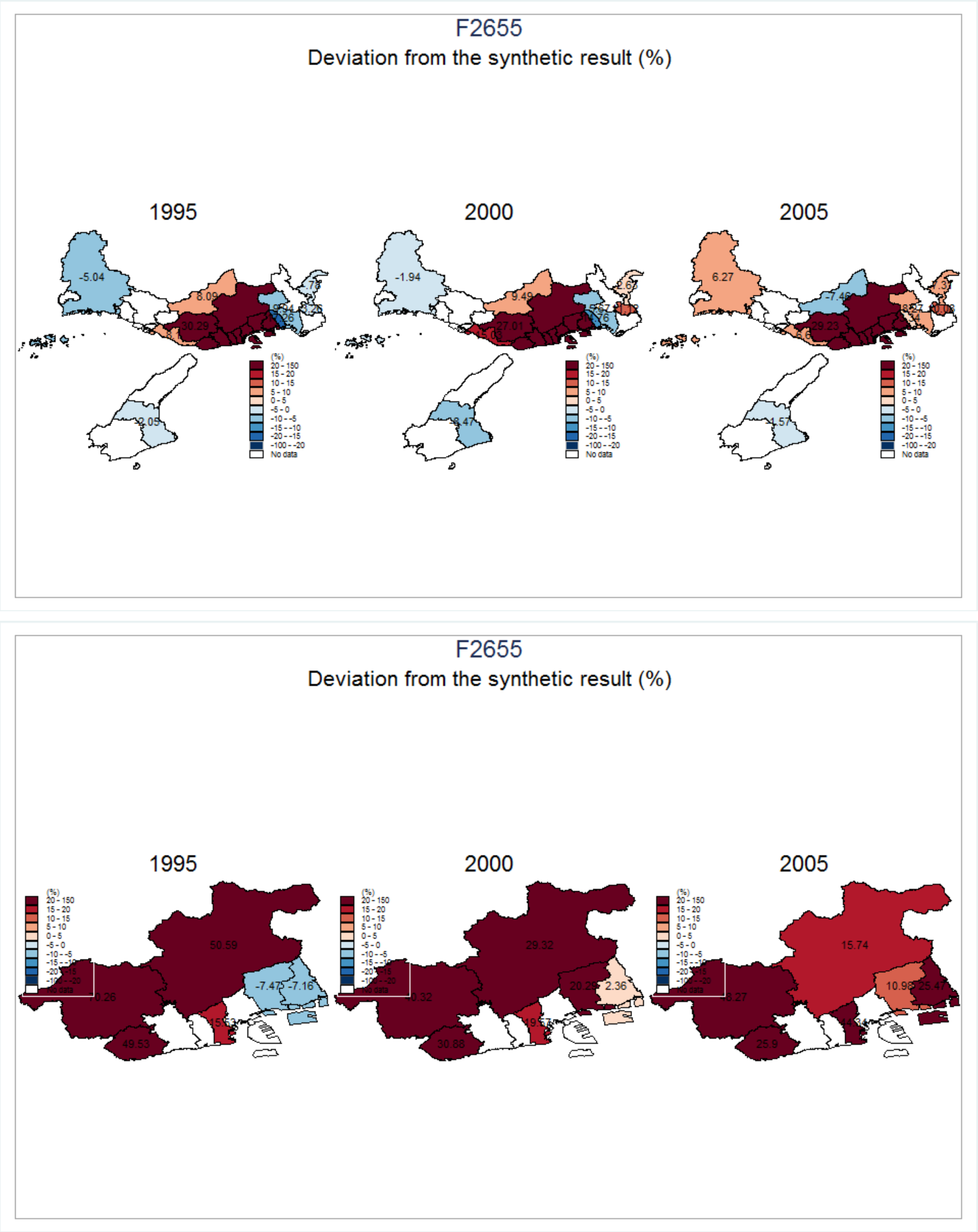

*Cities and wards with synthetic results that sufficiently reproduce the actual values prior to the Hanshin Awaji Earthquake (Jan.1995).

Figure 18. Placebos for Registered Population

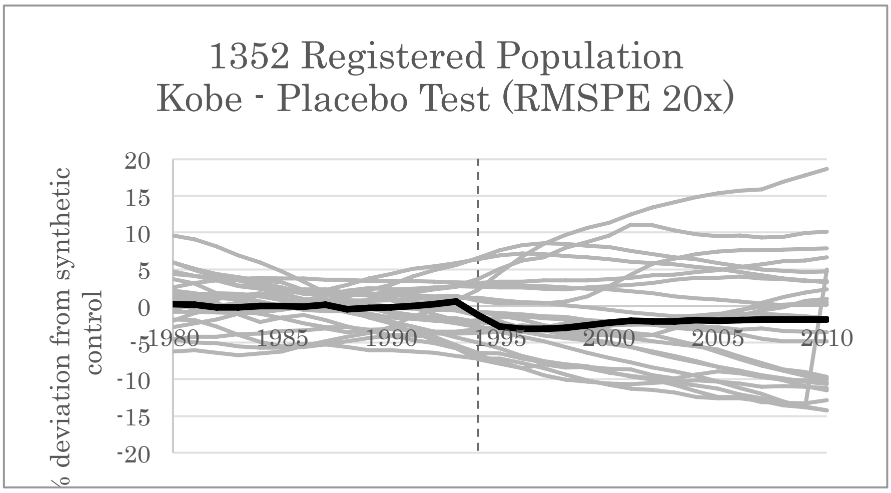

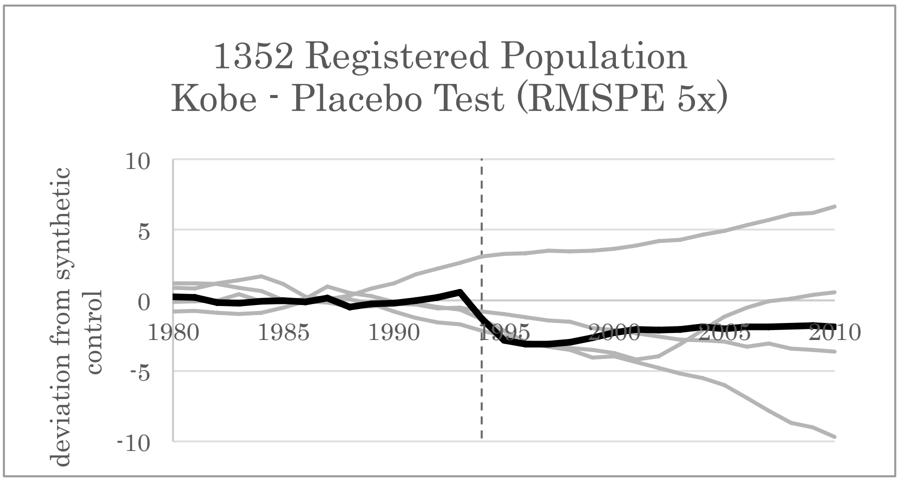

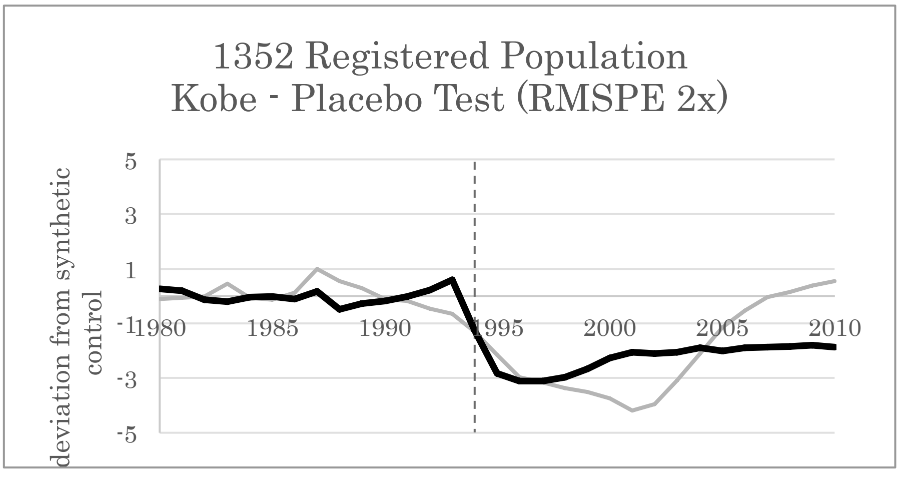

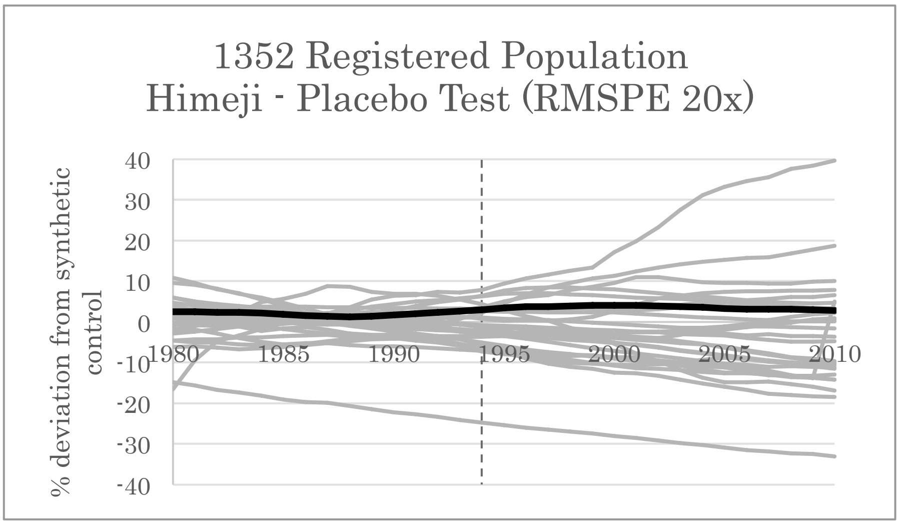
1352 Registered Population
Himeji - Placebo Test (RMSPE 20x)
% deviation from synthetic control
40
30
20
10
0
-10
-20
-30
-40
1980
1985
1990
1995
2000
2005
2010

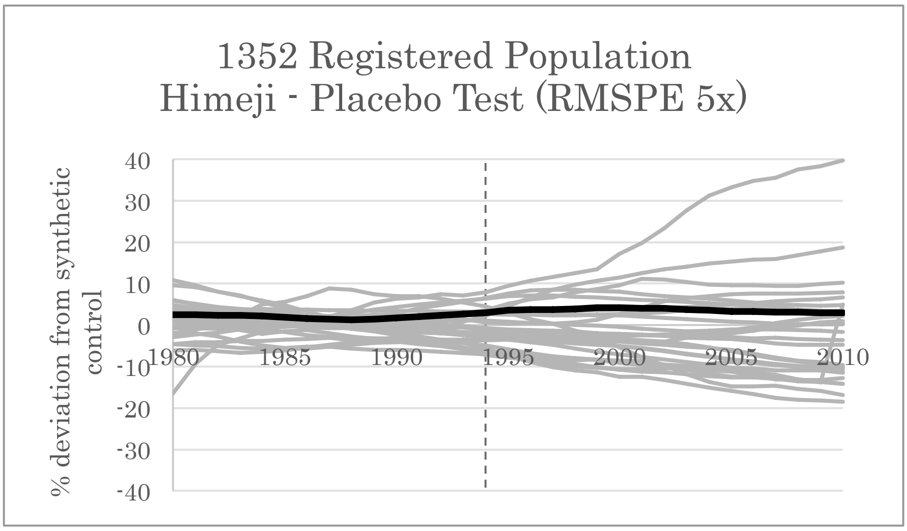
1352 Registered Population
Himeji - Placebo Test (RMSPE 5x)
% deviation from synthetic control
40
30
20
10
0
-10
-20
-30
-40
1980
1985
1990
1995
2000
2005
2010

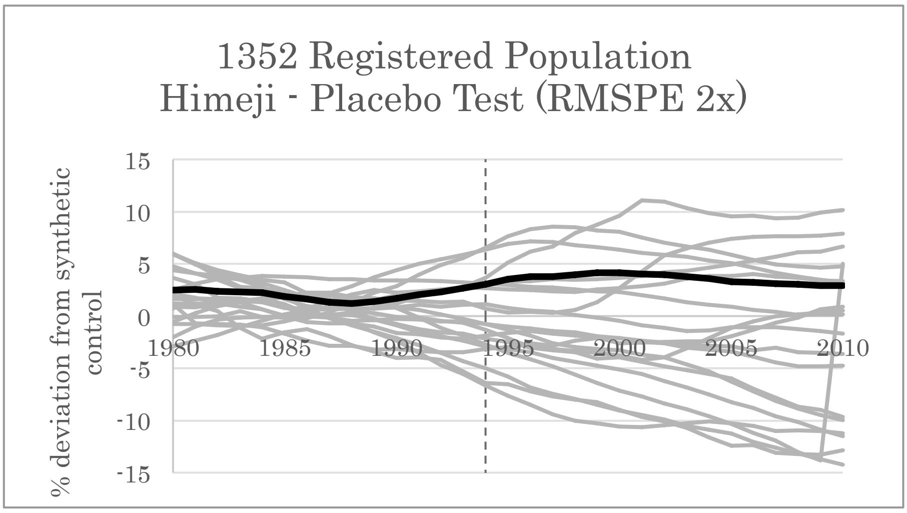
1352 Registered Population
Himeji - Placebo Test (RMSPE 2x)
% deviation from synthetic control
15
10
5
0
-5
-10
-15
1980
1985
1990
1995
2000
2005
2010

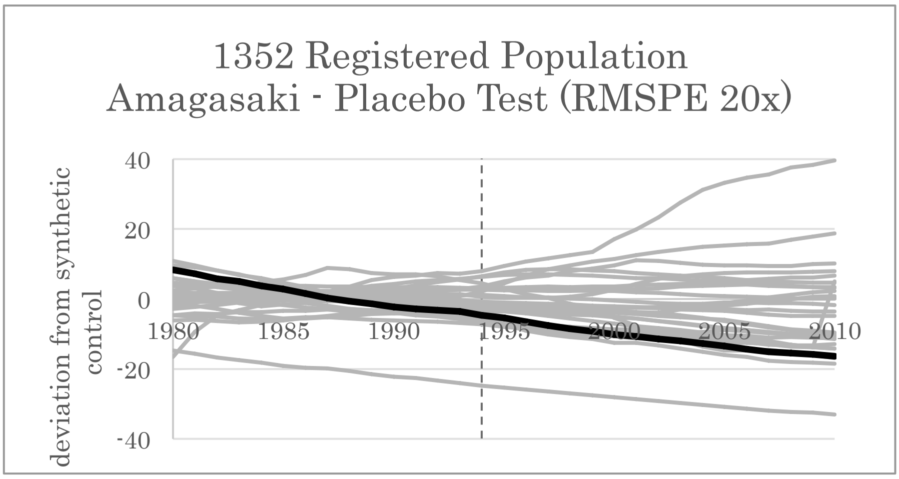
1352 Registered Population
Amagasaki - Placebo Test (RMSPE 20x)
deviation from synthetic control
40
20
0
-20
-40
1980
1985
1990
1995
2000
2005
2010

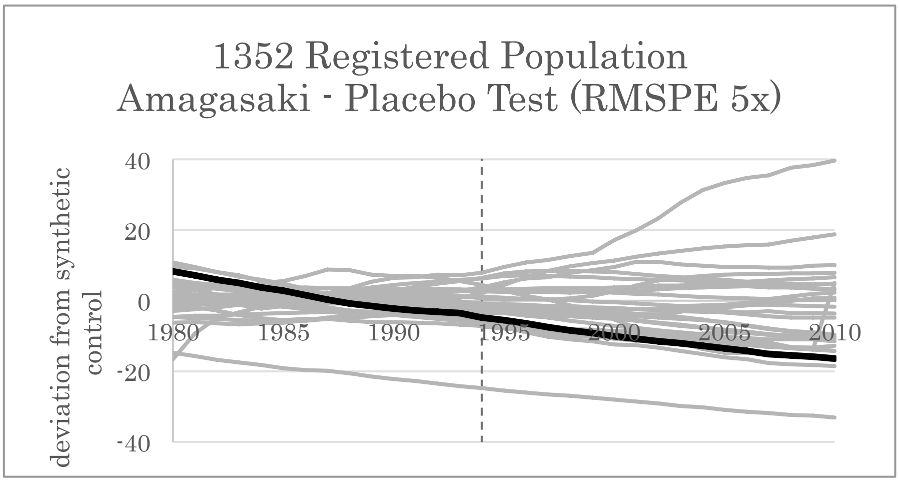
1352 Registered Population
Amagasaki - Placebo Test (RMSPE 5x)
deviation from synthetic control
40
20
0
-20
-40
1980
1985
1990
1995
2000
2005
2010

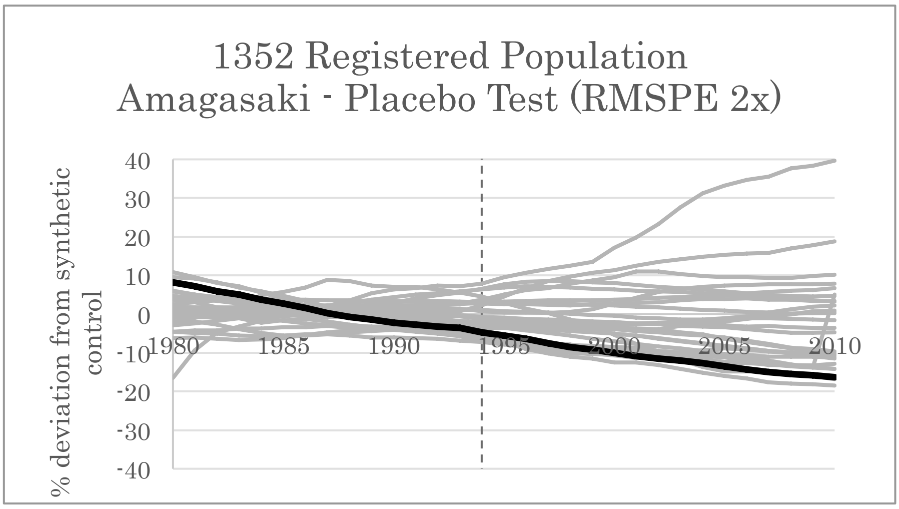
1352 Registered Population
Amagasaki - Placebo Test (RMSPE 2x)
% deviation from synthetic control
40
30
20
10
0
-10
-20
-30
-40
1980
1985
1990
1995
2000
2005
2010

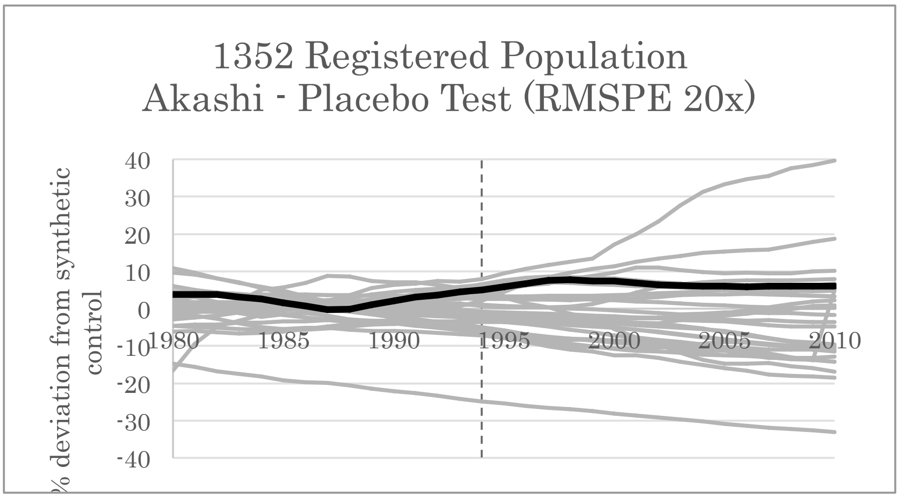
1352 Registered Population
Akashi - Placebo Test (RMSPE 20x)
% deviation from synthetic control
40
30
20
10
0
-10
-20
-30
-40
1980
1985
1990
1995
2000
2005
2010

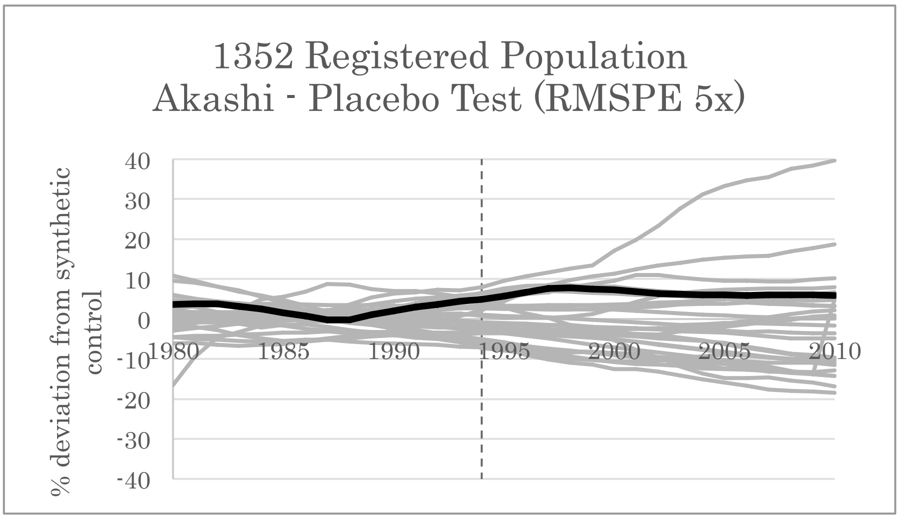
1352 Registered Population
Akashi - Placebo Test (RMSPE 5x)
% deviation from synthetic control
40
30
20
10
0
-10
-20
-30
-40
1980
1985
1990
1995
2000
2005
2010

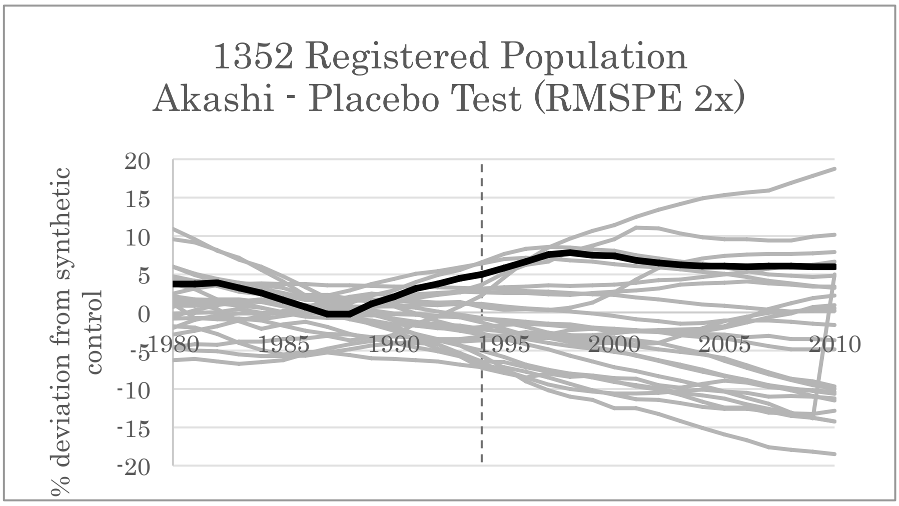
1352 Registered Population
Akashi - Placebo Test (RMSPE 2x)
% deviation from synthetic control
20
15
10
5
0
-5
-10
-15
-20
1980
1985
1990
1995
2000
2005
2010

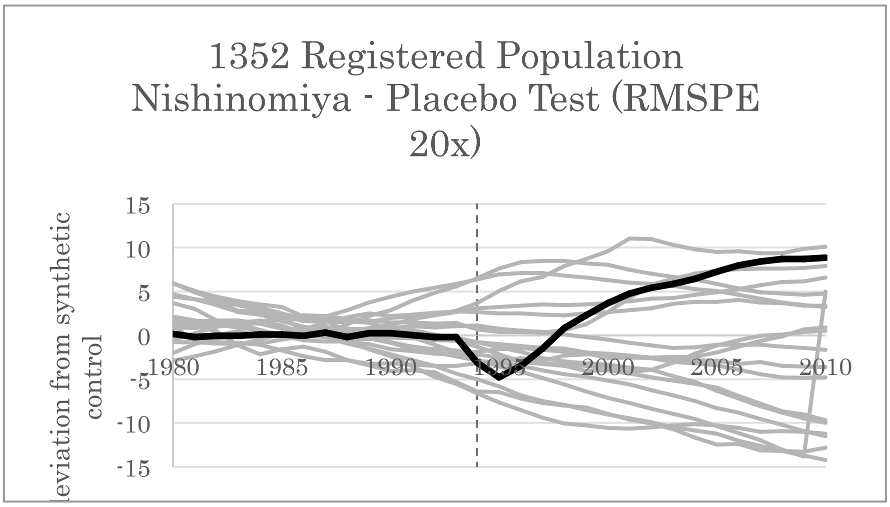
1352 Registered Population
Nishinomiya - Placebo Test (RMSPE
20x)
from synthetic
control
15
10
5
0
-5
-10
-15
1980
1985
1990
1995
2000
2005
2010

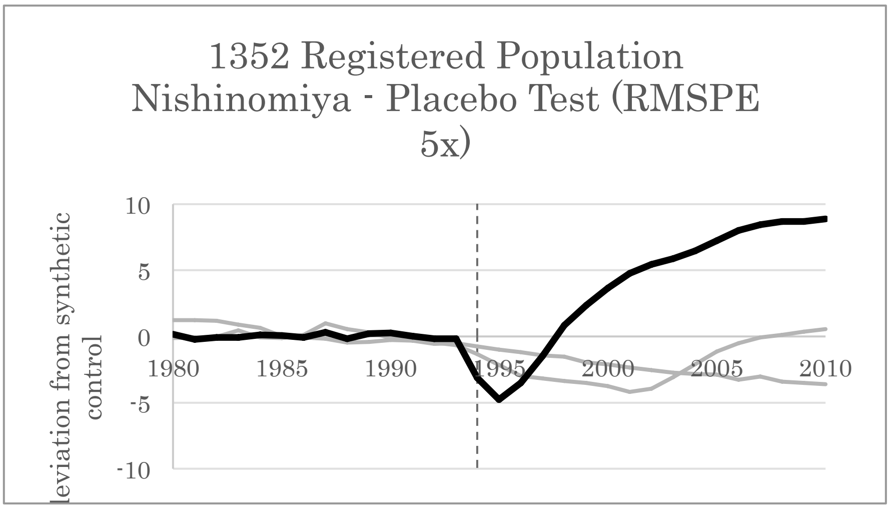
1352 Registered Population
Nishinomiya - Placebo Test (RMSPE
5x)
from synthetic
control
10
5
0
-5
-10
1980
1985
1990
1995
2000
2005
2010

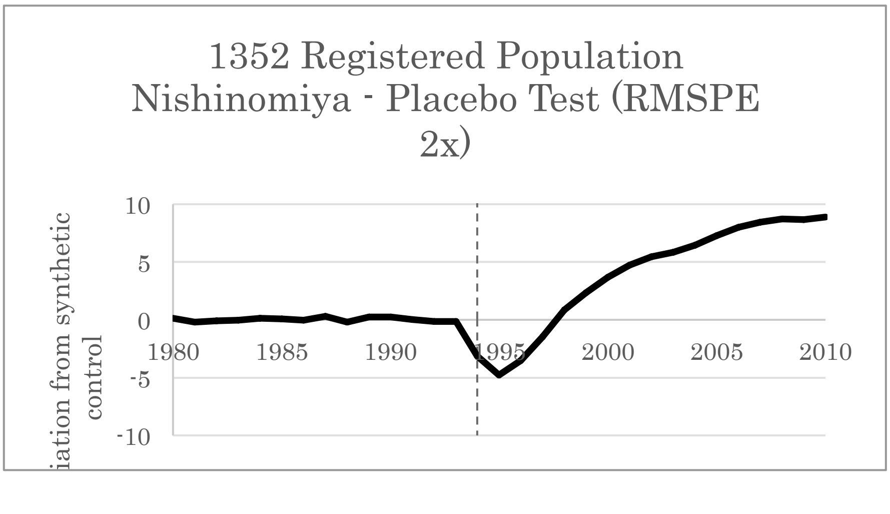
1352 Registered Population
Nishinomiya - Placebo Test (RMSPE
2x)
from synthetic
control
10
5
0
-5
-10
1980
1985
1990
1995
2000
2005
2010

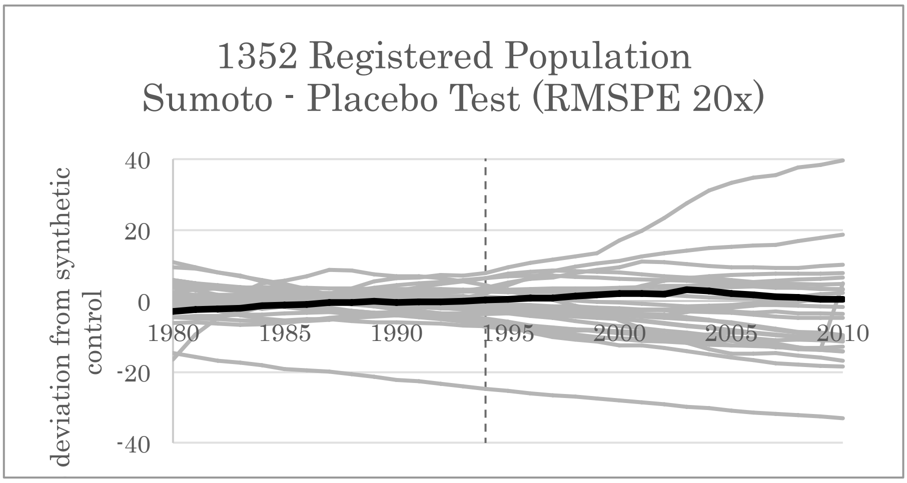
1352 Registered Population
Sumoto - Placebo Test (RMSPE 20x)
deviation from synthetic control
40
20
0
-20
-40
1980
1985
1990
1995
2000
2005
2010

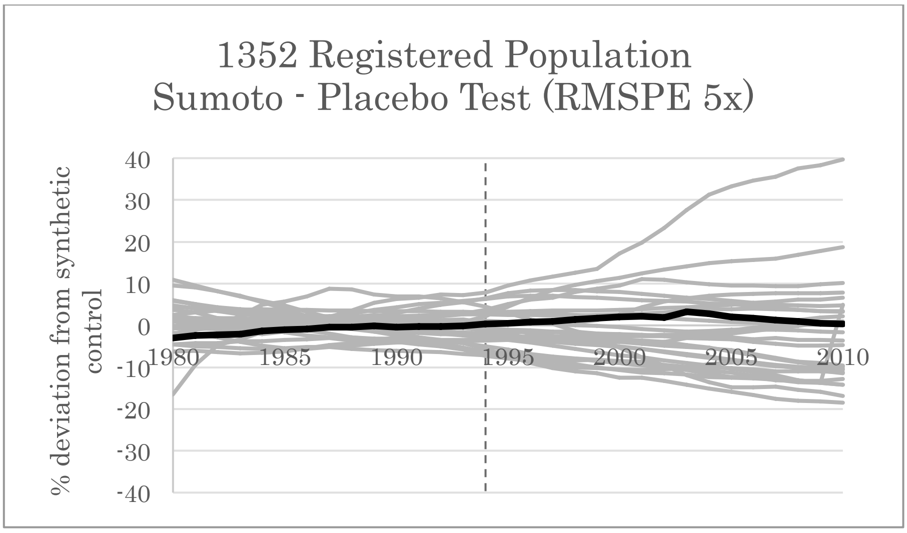
1352 Registered Population
Sumoto - Placebo Test (RMSPE 5x)
% deviation from synthetic control
40
30
20
10
0
-10
-20
-30
-40
1980
1985
1990
1995
2000
2005
2010

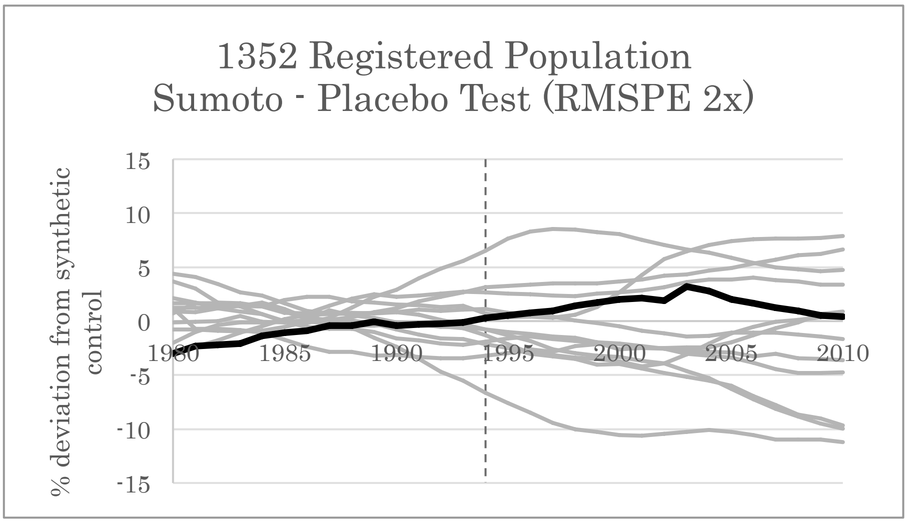
1352 Registered Population
Sumoto - Placebo Test (RMSPE 2x)
% deviation from synthetic control
15
10
5
0
-5
-10
-15
1980
1985
1990
1995
2000
2005
2010

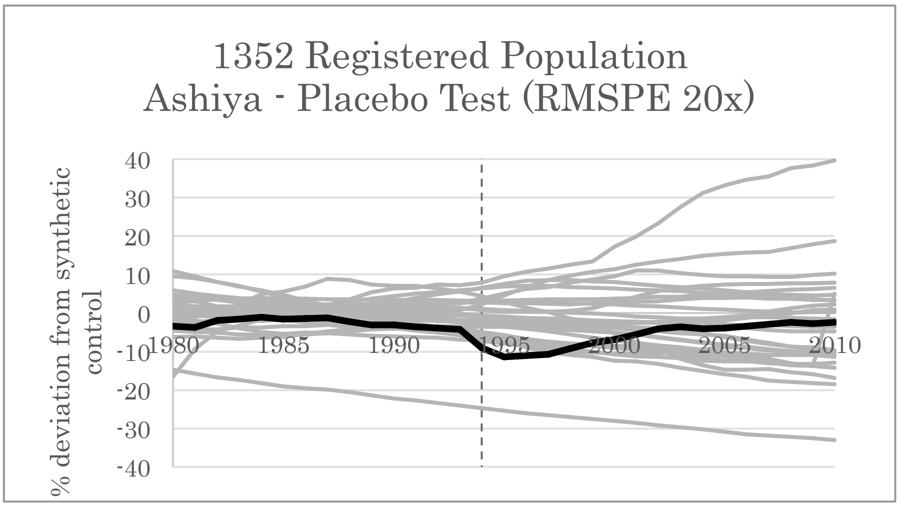
1352 Registered Population
Ashiya - Placebo Test (RMSPE 20x)
% deviation from synthetic control
40
30
20
10
0
-10
-20
-30
-40
1980
1985
1990
1995
2000
2005
2010

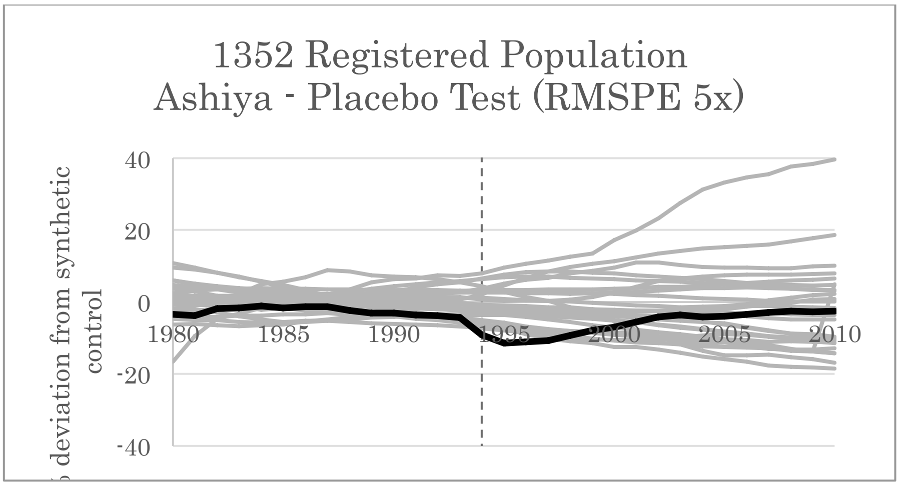
1352 Registered Population
Ashiya - Placebo Test (RMSPE 5x)
% deviation from synthetic control
40
20
0
-20
-40
1980
1985
1990
1995
2000
2005
2010

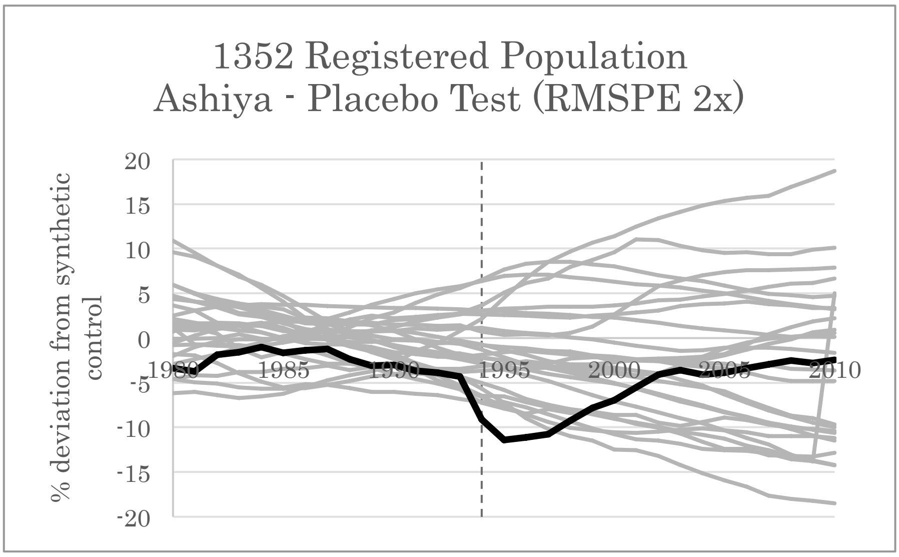
1352 Registered Population
Ashiya - Placebo Test (RMSPE 2x)
% deviation from synthetic control
20
15
10
5
0
-5
-10
-15
-20
1980
1985
1990
1995
2000
2005
2010

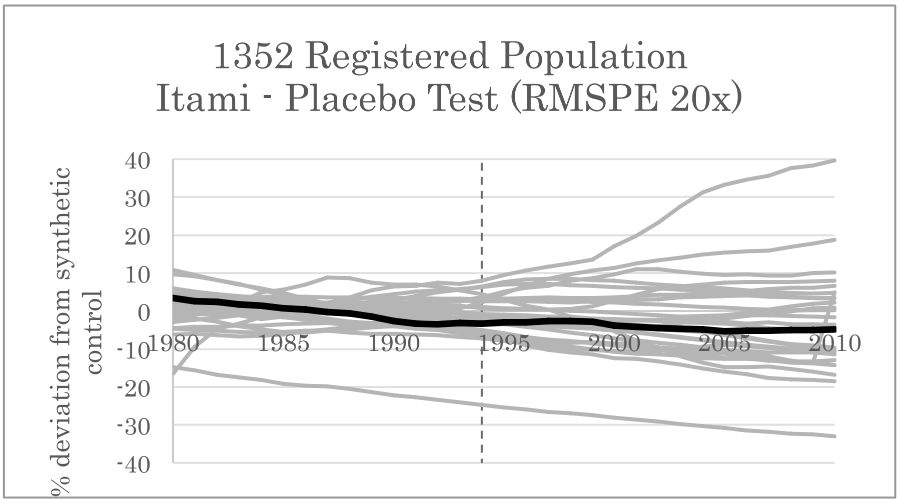

1352 Registered Population
Itami - Placebo Test (RMSPE 20x)
% deviation from synthetic control
40
30
20
10
0
-10
-20
-30
-40
1980
1985
1990
1995
2000
2005
2010

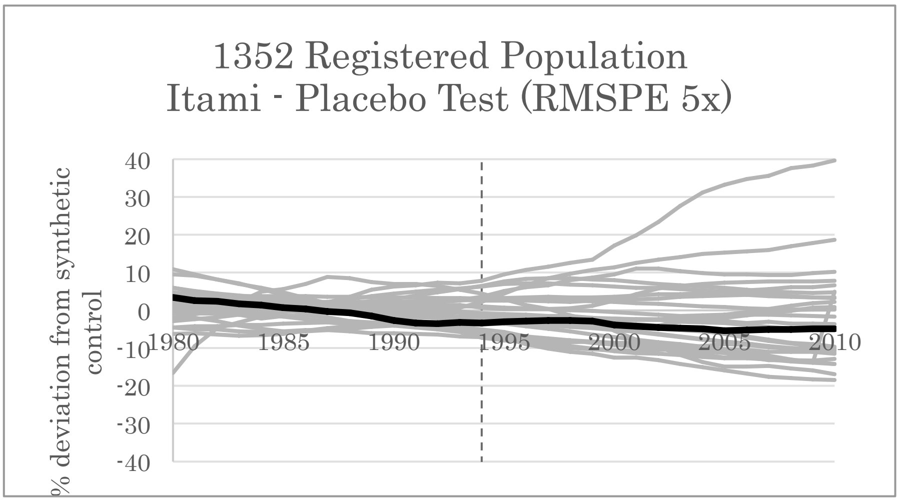

1352 Registered Population
Itami - Placebo Test (RMSPE 5x)
% deviation from synthetic control
40
30
20
10
0
-10
-20
-30
-40
1980
1985
1990
1995
2000
2005
2010

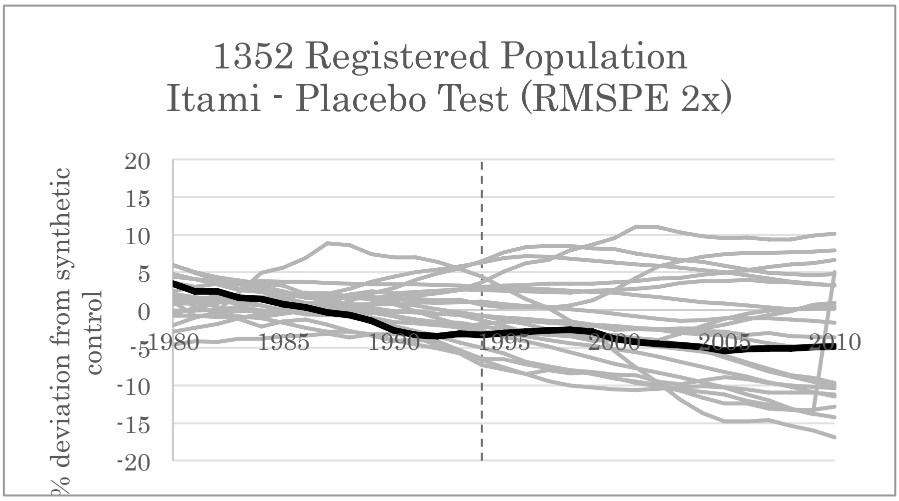

1352 Registered Population
Itami - Placebo Test (RMSPE 2x)
% deviation from synthetic control
20
15
10
5
0
-5
-10
-15
-20
1980
1985
1990
1995
2000
2005
2010

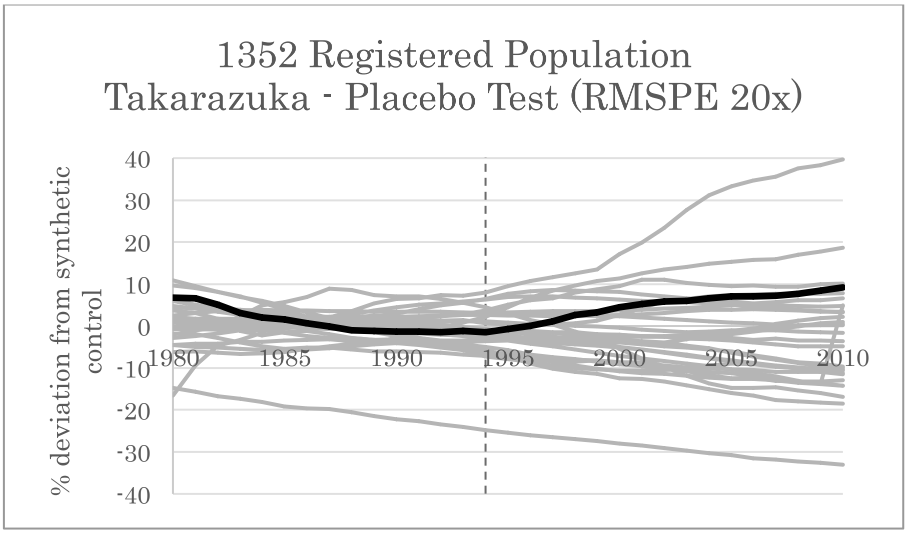
1352 Registered Population
Takarazuka - Placebo Test (RMSPE 20x)
% deviation from synthetic control
40
30
20
10
0
-10
-20
-30
-40
1980
1985
1990
1995
2000
2005
2010

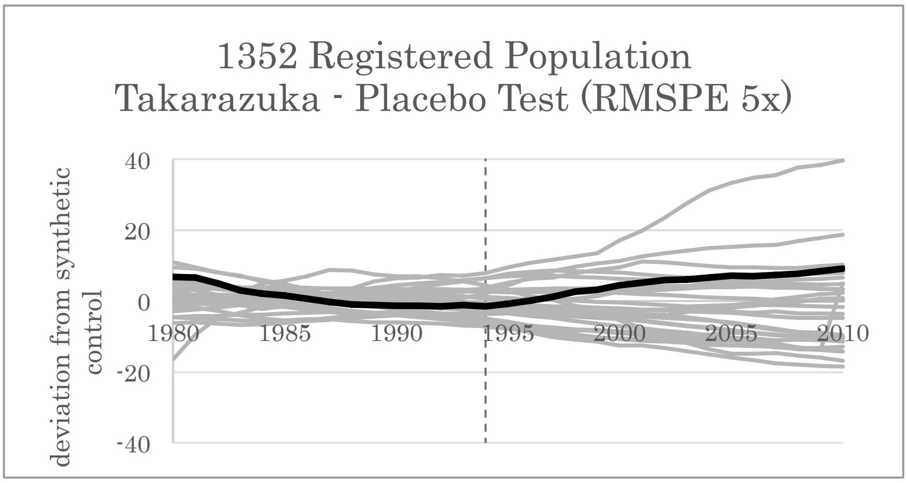
1352 Registered Population
Takarazuka - Placebo Test (RMSPE 5x)
deviation from synthetic control
40
20
0
-20
-40
1980
1985
1990
1995
2000
2005
2010

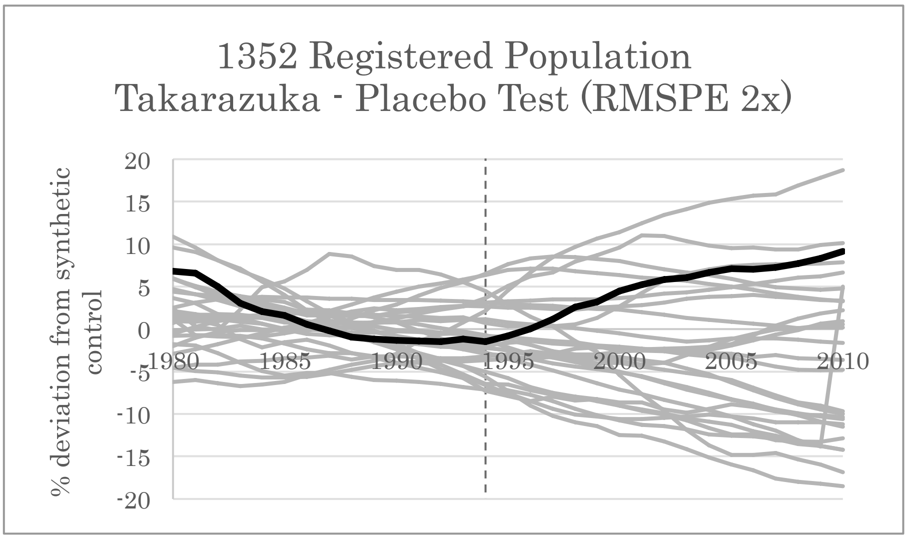
1352 Registered Population
Takarazuka - Placebo Test (RMSPE 2x)
% deviation from synthetic control
20
15
10
5
0
-5
-10
-15
-20
1980
1985
1990
1995
2000
2005
2010

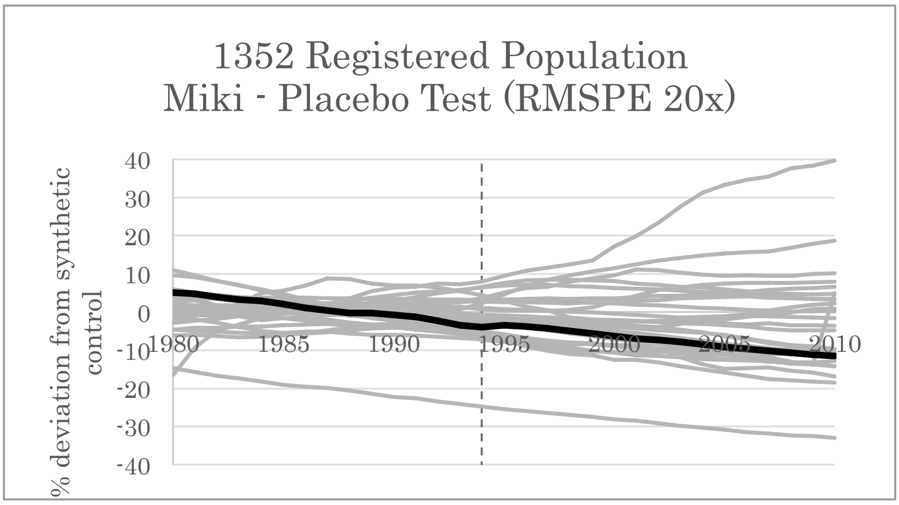
1352 Registered Population
Miki - Placebo Test (RMSPE 20x)
% deviation from synthetic control
40
30
20
10
0
-10
-20
-30
-40
1980
1985
1990
1995
2000
2005
2010

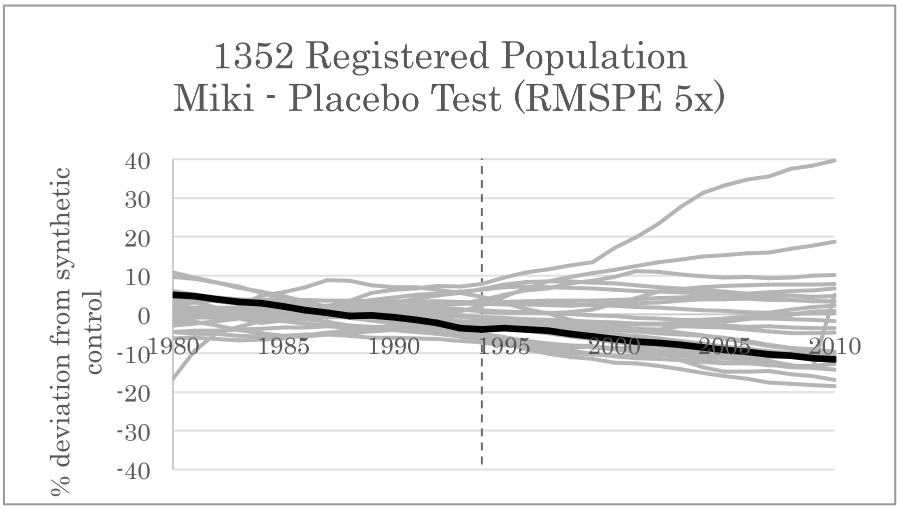
1352 Registered Population
Miki - Placebo Test (RMSPE 5x)
% deviation from synthetic control
40
30
20
10
0
-10
-20
-30
-40
1980
1985
1990
1995
2000
2005
2010

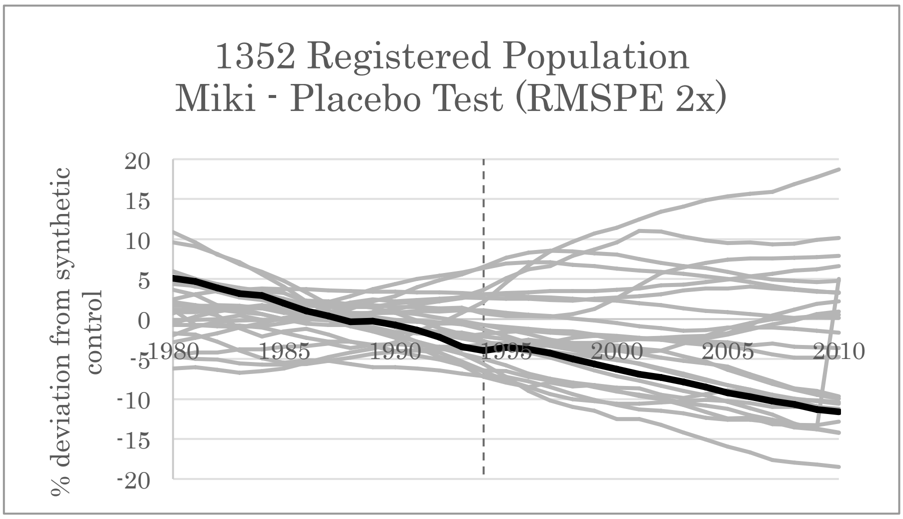
1352 Registered Population
Miki - Placebo Test (RMSPE 2x)
% deviation from synthetic control
20
15
10
5
0
-5
-10
-15
-20
1980
1985
1990
1995
2000
2005
2010

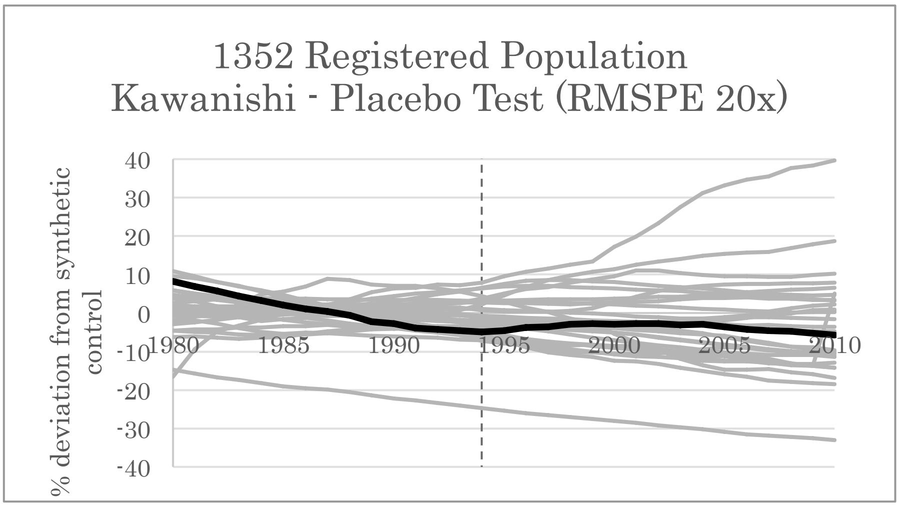
1352 Registered Population
Kawanishi - Placebo Test (RMSPE 20x)
% deviation from synthetic control
40
30
20
10
0
-10
-20
-30
-40
1980
1985
1990
1995
2000
2005
2010

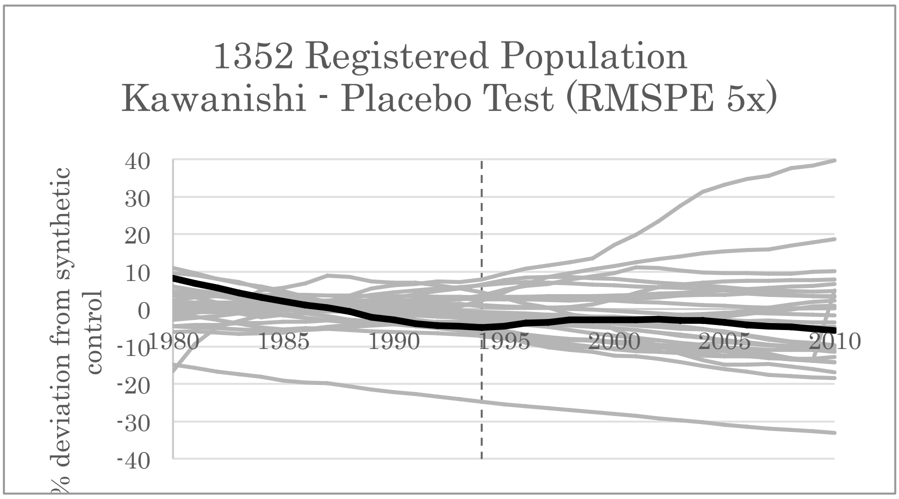
1352 Registered Population
Kawanishi - Placebo Test (RMSPE 5x)
% deviation from synthetic control
40
30
20
10
0
-10
-20
-30
-40
1980
1985
1990
1995
2000
2005
2010

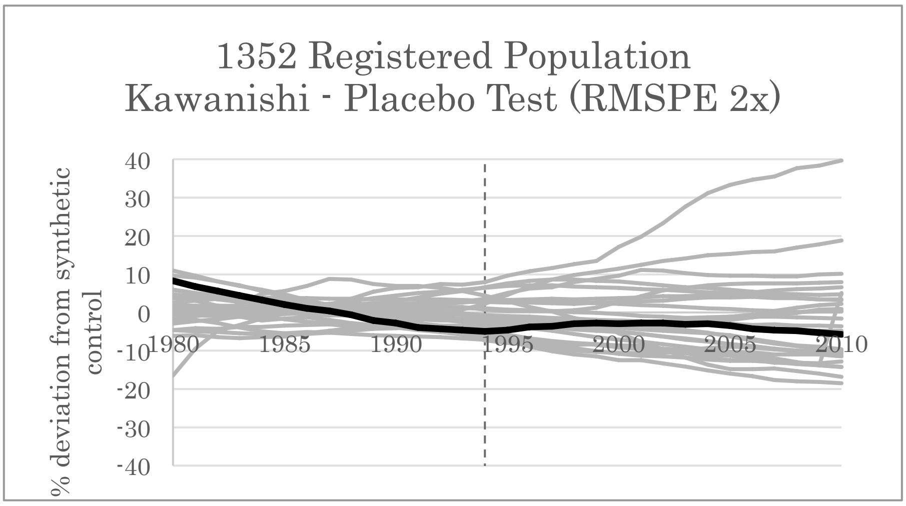
1352 Registered Population
Kawanishi - Placebo Test (RMSPE 2x)
% deviation from synthetic control
40
30
20
10
0
-10
-20
-30
-40
1980
1985
1990
1995
2000
2005
2010

Figure 19. Placebos for Taxable Income

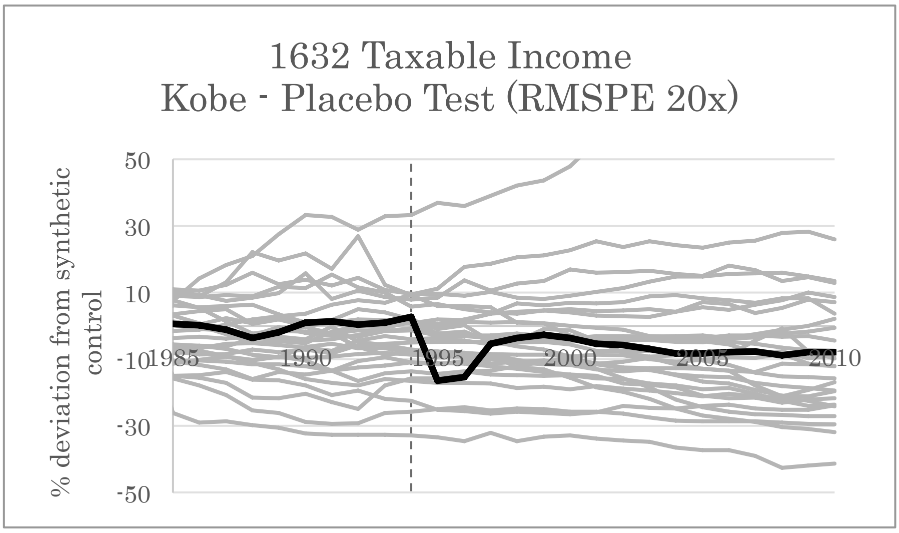

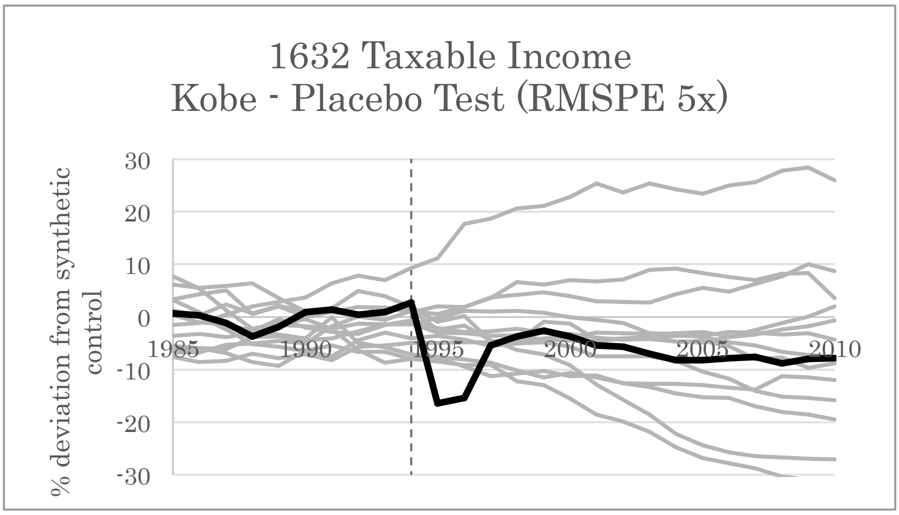

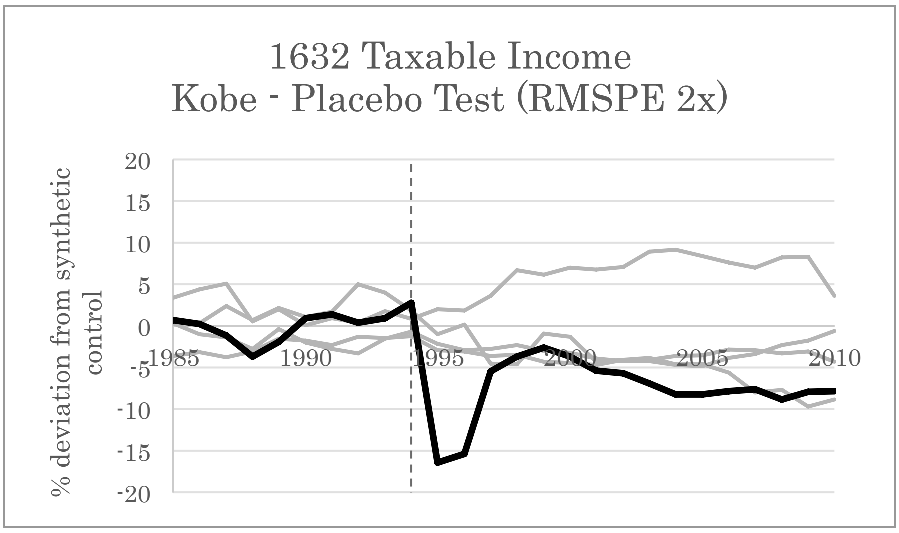

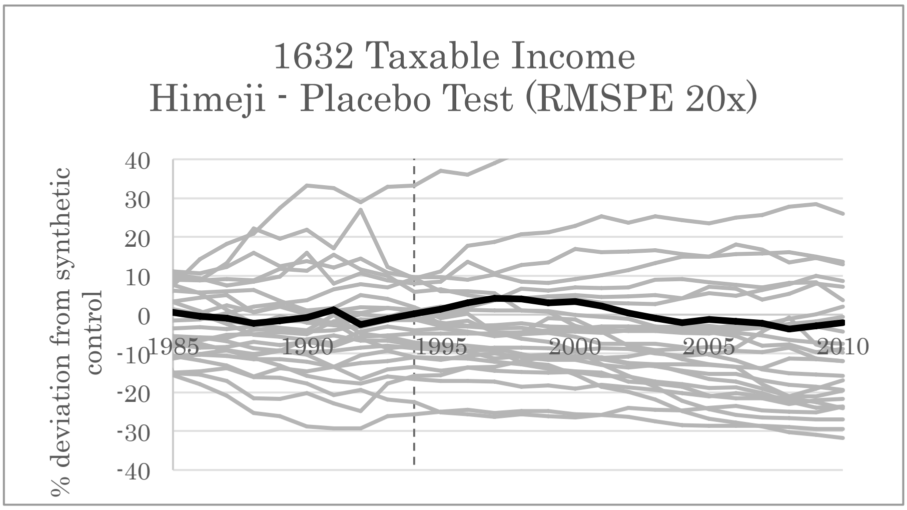
1632 Taxable Income
Himeji - Placebo Test (RMSPE 20x)
% deviation from synthetic control
40
30
20
10
0
-10
-20
-30
-40
1985
1990
1995
2000
2005
2010

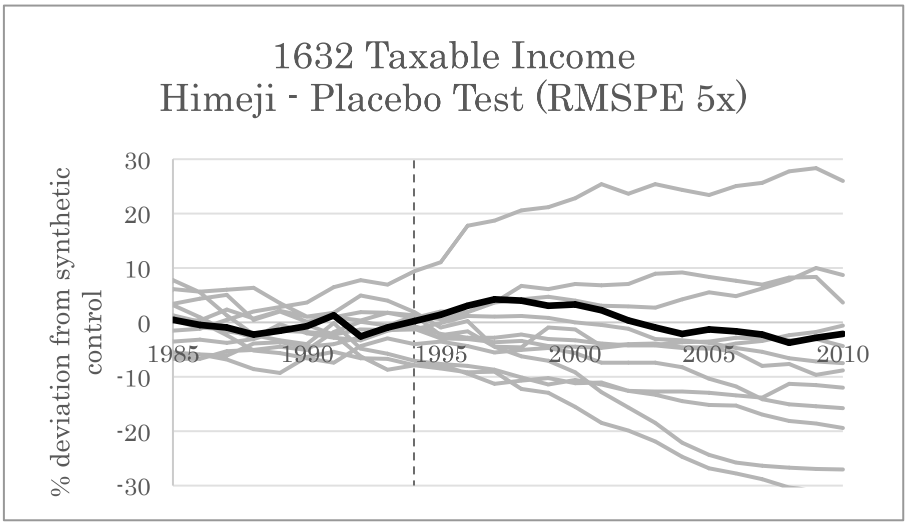
1632 Taxable Income
Himeji - Placebo Test (RMSPE 5x)
% deviation from synthetic control
30
20
10
0
-10
-20
-30
1985
1990
1995
2000
2005
2010

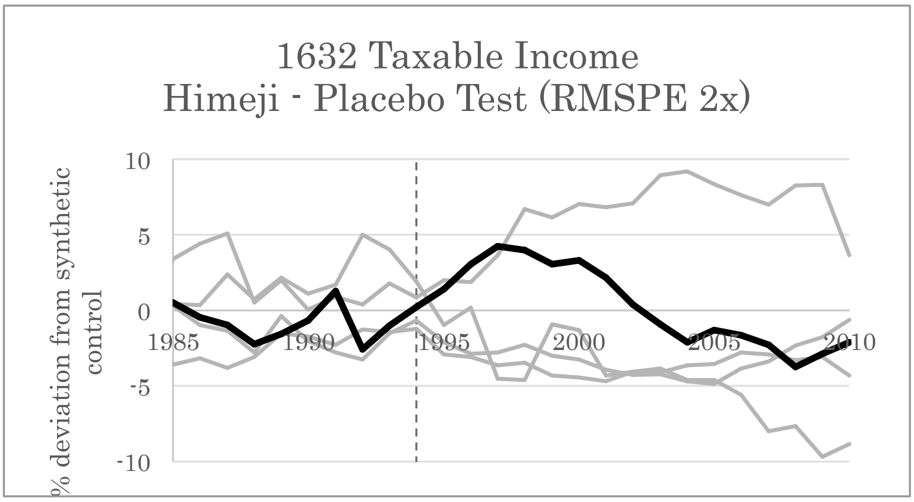
1632 Taxable Income
Himeji - Placebo Test (RMSPE 2x)
% deviation from synthetic control
10
5
0
-5
-10
1985
1990
1995
2000
2005
2010

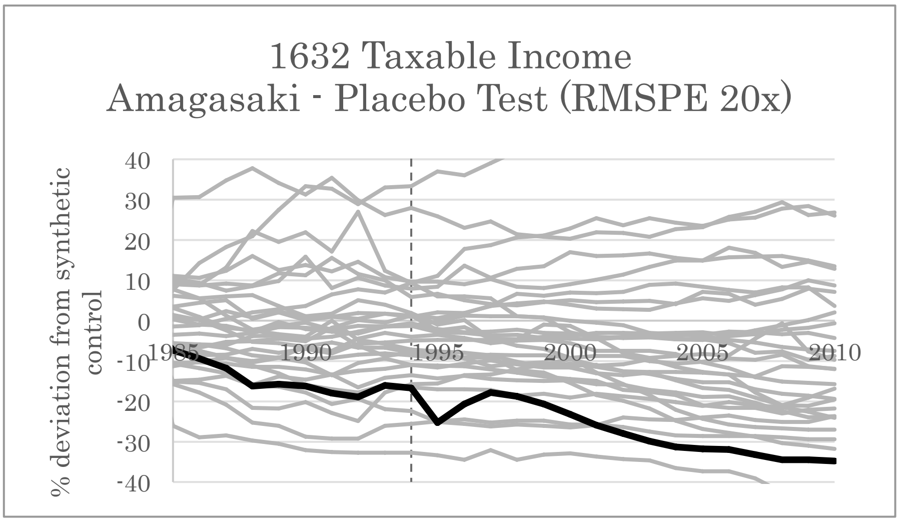
1632 Taxable Income
Amagasaki - Placebo Test (RMSPE 20x)
% deviation from synthetic control
40
30
20
10
0
-10
-20
-30
-40
1985
1990
1995
2000
2005
2010

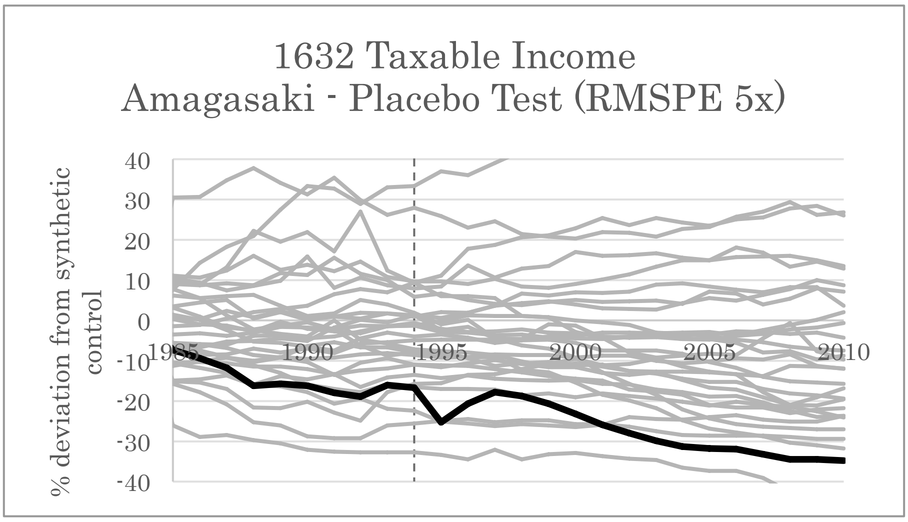
1632 Taxable Income
Amagasaki - Placebo Test (RMSPE 5x)
% deviation from synthetic control
40
30
20
10
0
-10
-20
-30
-40
1985
1990
1995
2000
2005
2010

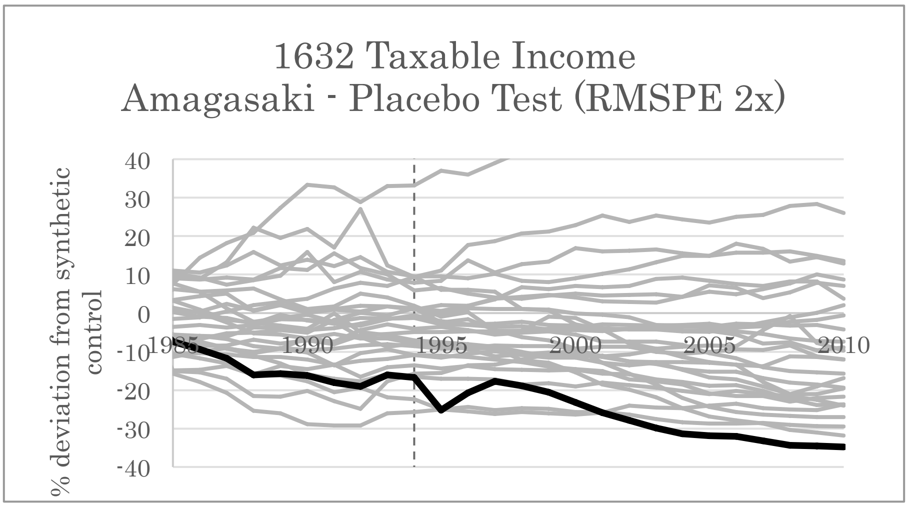
1632 Taxable Income
Amagasaki - Placebo Test (RMSPE 2x)
% deviation from synthetic control
40
30
20
10
0
-10
-20
-30
-40
1985
1990
1995
2000
2005
2010

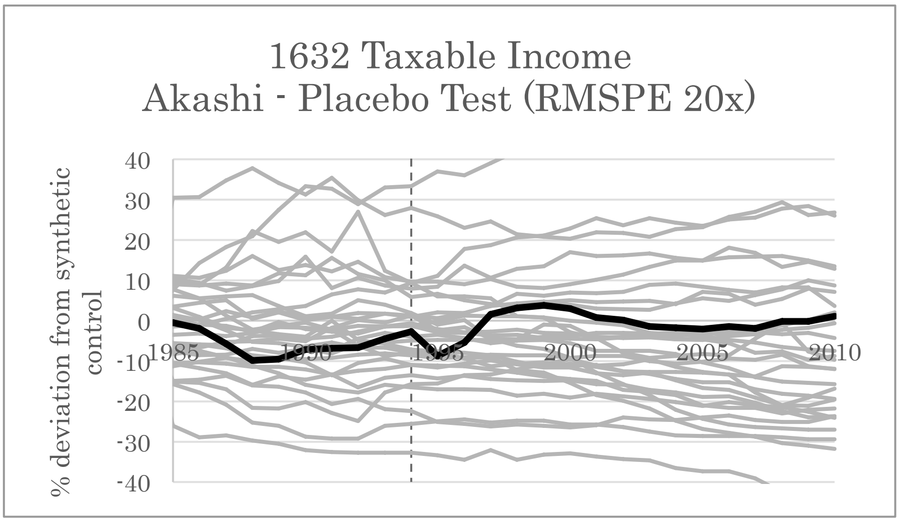
1632 Taxable Income
Akashi - Placebo Test (RMSPE 20x)
% deviation from synthetic control
40
30
20
10
0
-10
-20
-30
-40
1985
1990
1995
2000
2005
2010

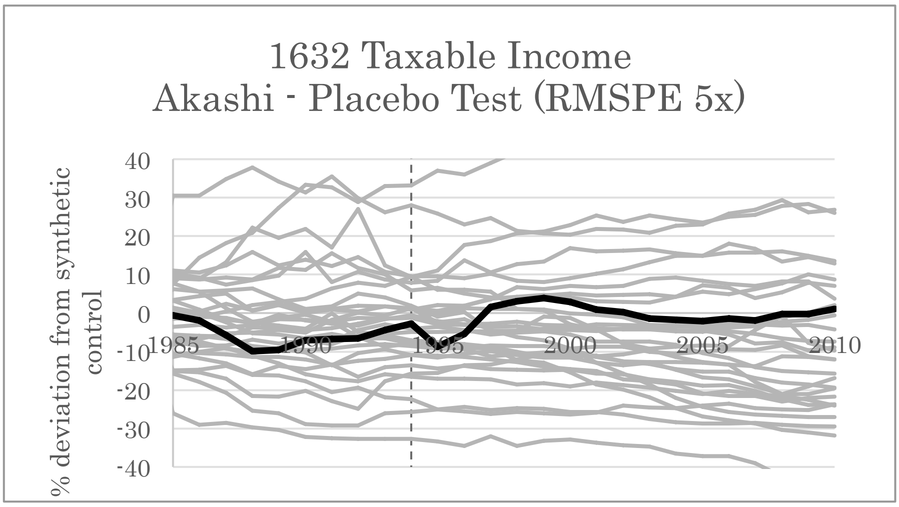
1632 Taxable Income
Akashi - Placebo Test (RMSPE 5x)
% deviation from synthetic control
40
30
20
10
0
-10
-20
-30
-40
1985
1990
1995
2000
2005
2010

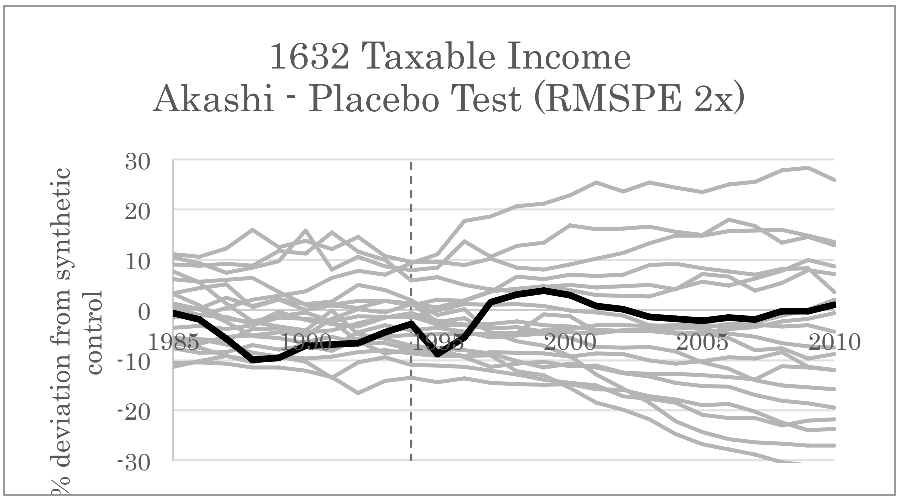
1632 Taxable Income
Akashi - Placebo Test (RMSPE 2x)
% deviation from synthetic control
30
20
10
0
-10
-20
-30
1985
1990
1995
2000
2005
2010

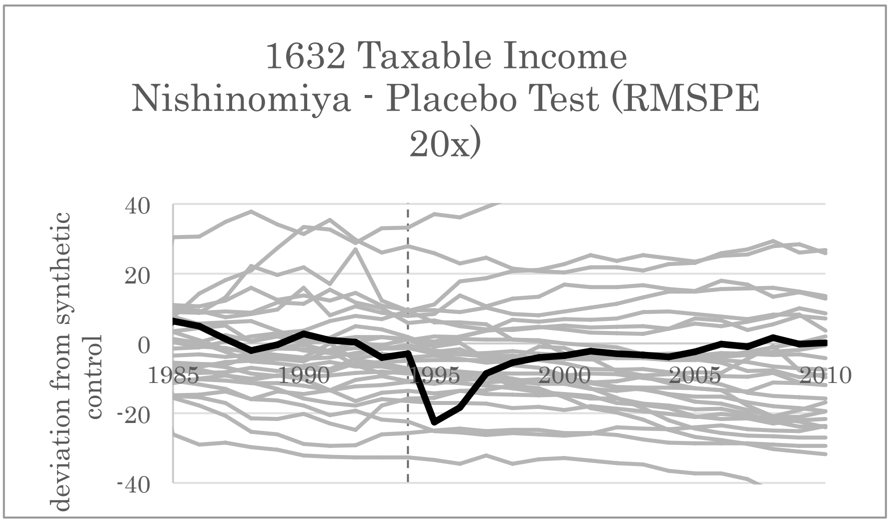
1632 Taxable Income
Nishinomiya - Placebo Test (RMSPE
20x)
deviation from synthetic
control
40
20
0
-20
-40
1985
1990
1995
2000
2005
2010

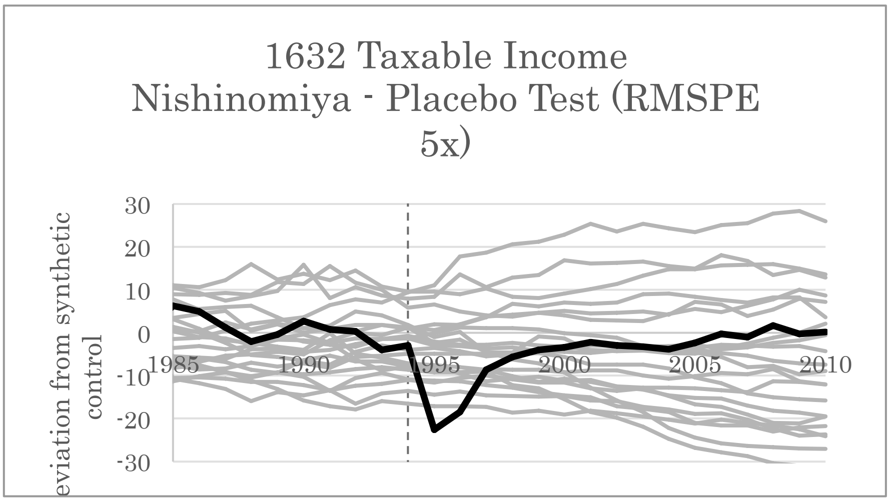
1632 Taxable Income
Nishinomiya - Placebo Test (RMSPE
5x)
eviation from synthetic
control
30
20
10
0
-10
-20
-30
1985
1990
1995
2000
2005
2010

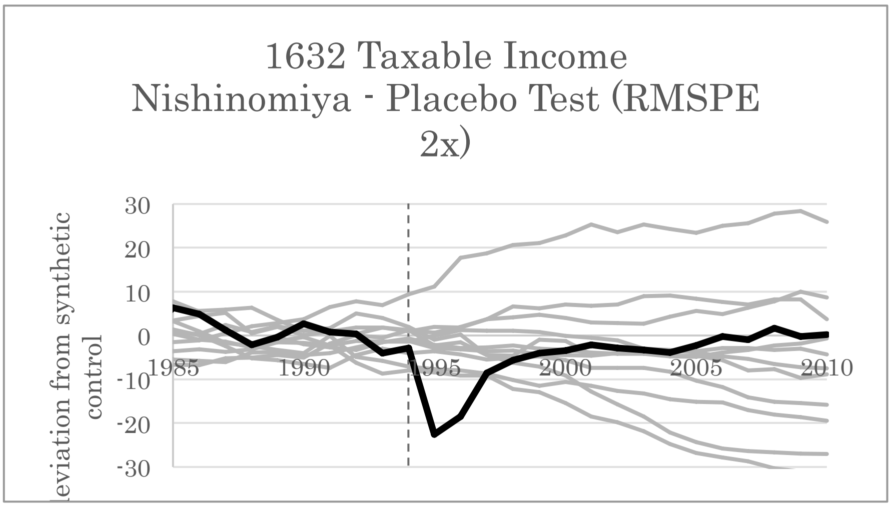
1632 Taxable Income
Nishinomiya - Placebo Test (RMSPE
2x)
eviation from synthetic
control
30
20
10
0
-10
-20
-30
1985
1990
1995
2000
2005
2010

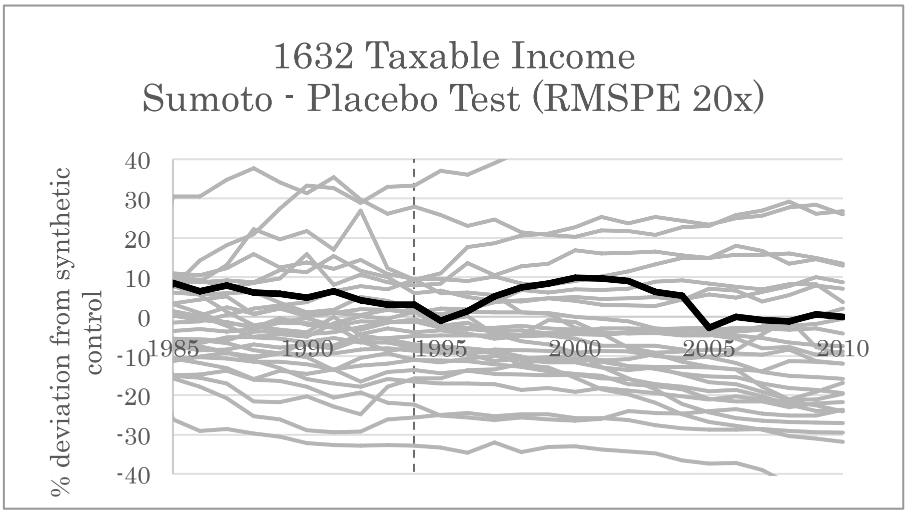
1632 Taxable Income
Sumoto - Placebo Test (RMSPE 20x)
% deviation from synthetic control
40
30
20
10
0
-10
-20
-30
-40
1985
1990
1995
2000
2005
2010

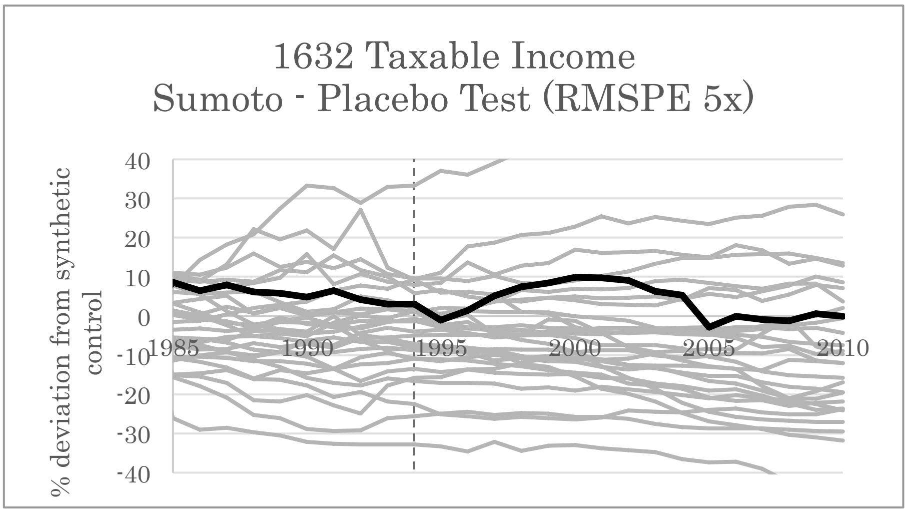
1632 Taxable Income
Sumoto - Placebo Test (RMSPE 5x)
% deviation from synthetic control
40
30
20
10
0
-10
-20
-30
-40
1985
1990
1995
2000
2005
2010

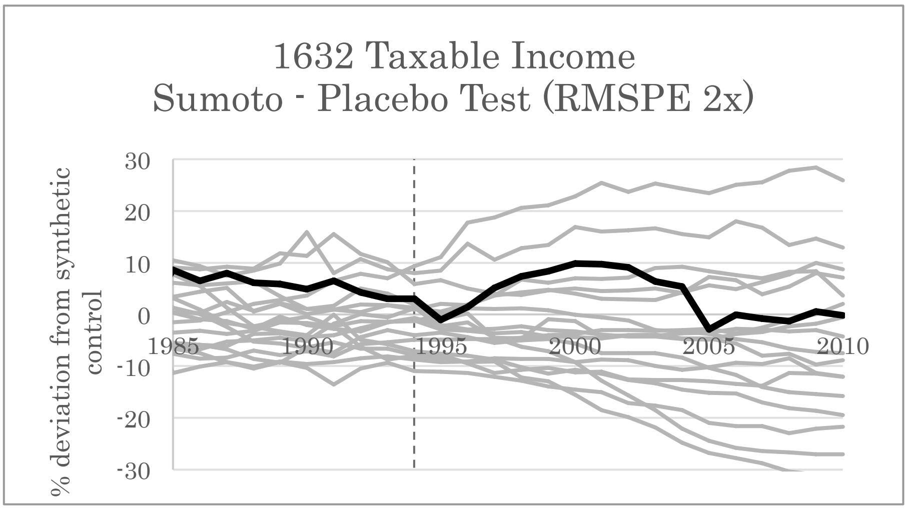
1632 Taxable Income
Sumoto - Placebo Test (RMSPE 2x)
% deviation from synthetic control
30
20
10
0
-10
-20
-30
1985
1990
1995
2000
2005
2010

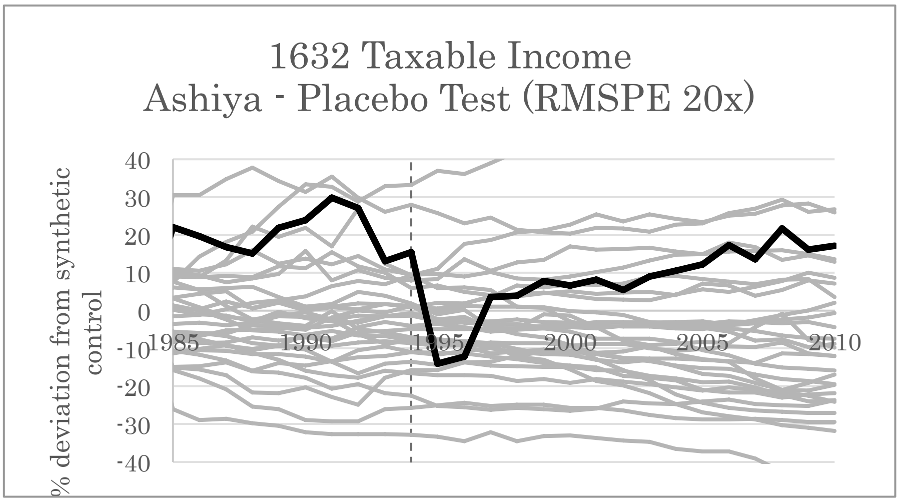
1632 Taxable Income
Ashiya - Placebo Test (RMSPE 20x)
% deviation from synthetic control
40
30
20
10
0
-10
-20
-30
-40
1985
1990
1995
2000
2005
2010

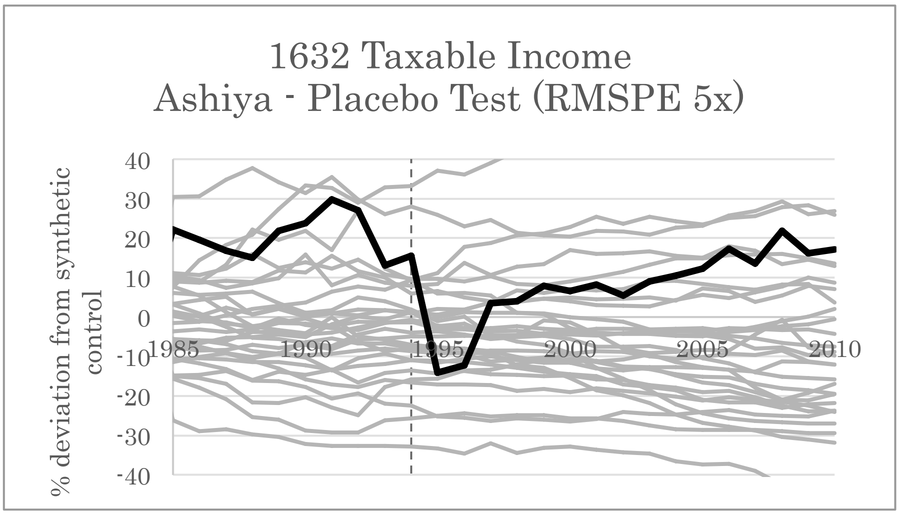
1632 Taxable Income
Ashiya - Placebo Test (RMSPE 5x)
% deviation from synthetic control
40
30
20
10
0
-10
-20
-30
-40
1985
1990
1995
2000
2005
2010

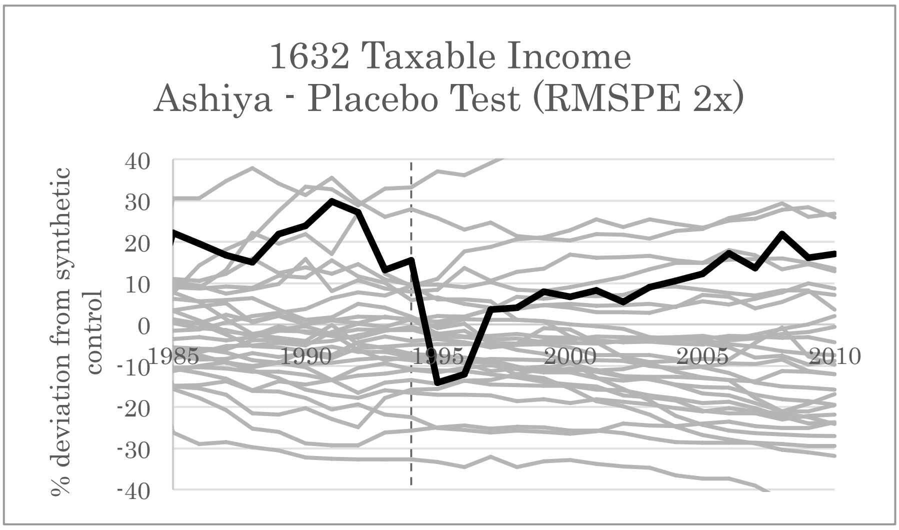
1632 Taxable Income
Ashiya - Placebo Test (RMSPE 2x)
% deviation from synthetic control
40
30
20
10
0
-10
-20
-30
-40
1985
1990
1995
2000
2005
2010

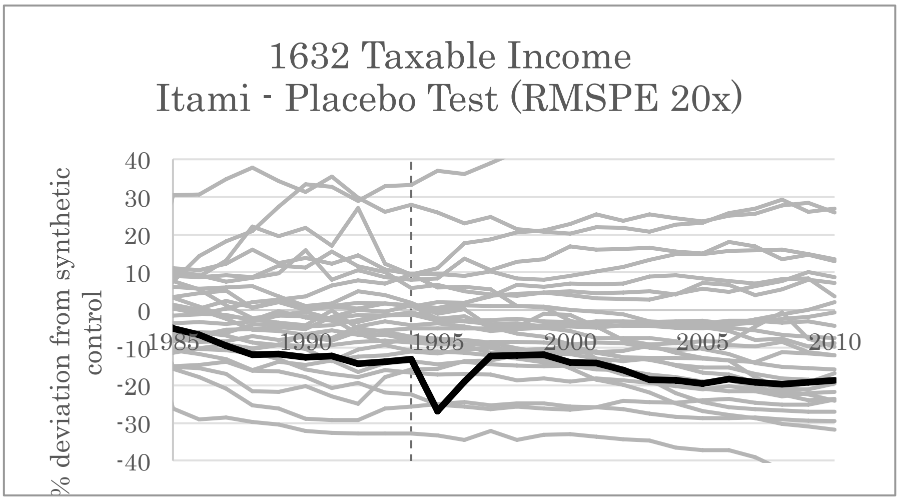

1632 Taxable Income
Itami - Placebo Test (RMSPE 20x)
% deviation from synthetic control
40
30
20
10
0
-10
-20
-30
-40
1985
1990
1995
2000
2005
2010

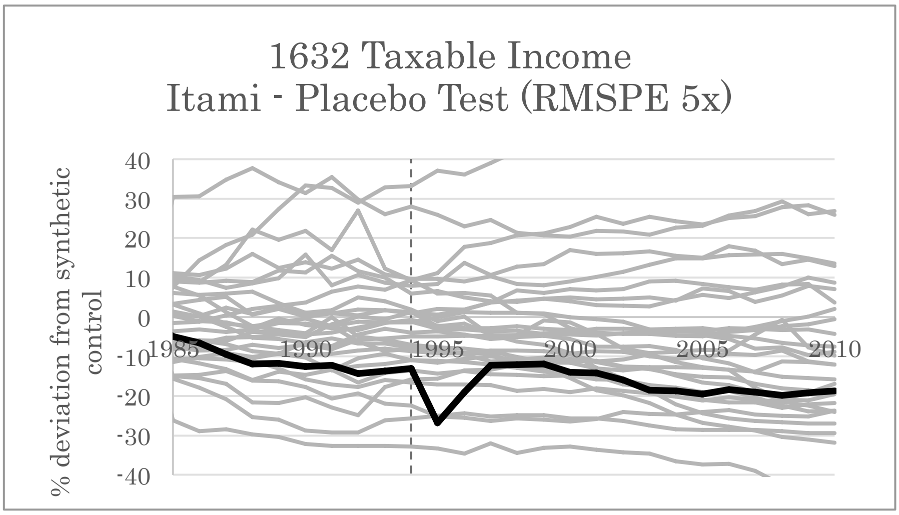

1632 Taxable Income
Itami - Placebo Test (RMSPE 5x)
% deviation from synthetic control
40
30
20
10
0
-10
-20
-30
-40
1985
1990
1995
2000
2005
2010

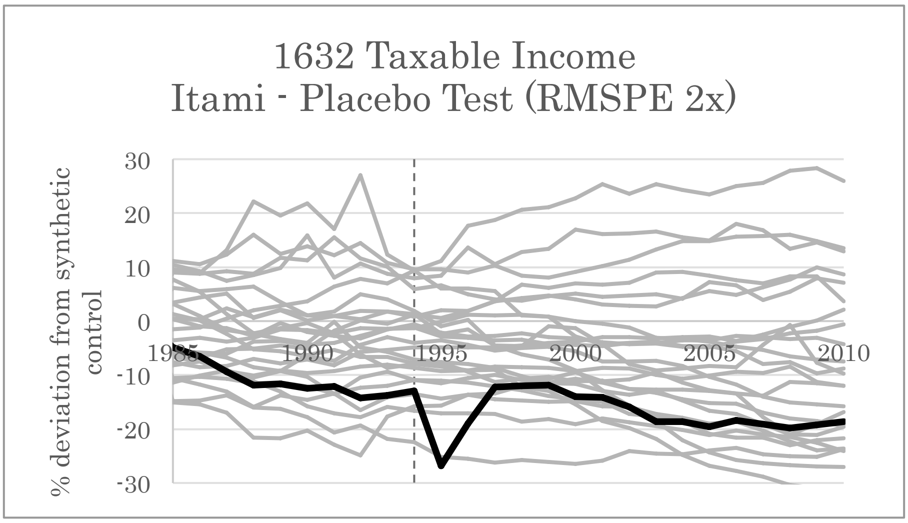

1632 Taxable Income
Itami - Placebo Test (RMSPE 2x)
% deviation from synthetic control
30
20
10
0
-10
-20
-30
1985
1990
1995
2000
2005
2010

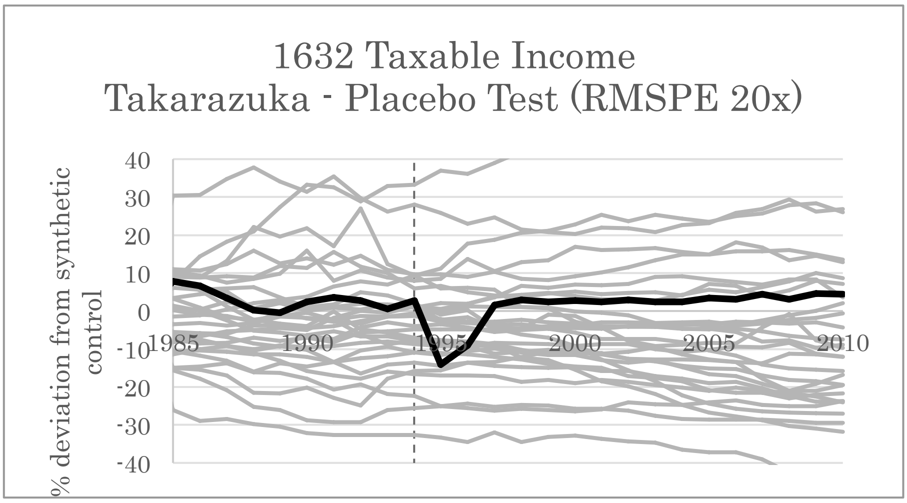
1632 Taxable Income
Takarazuka - Placebo Test (RMSPE 20x)
% deviation from synthetic control
40
30
20
10
0
-10
-20
-30
-40
1985
1990
1995
2000
2005
2010

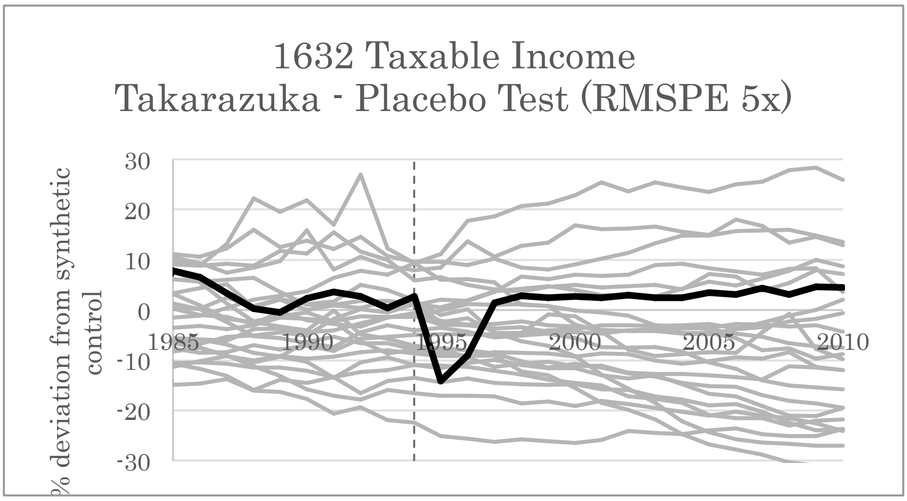
1632 Taxable Income
Takarazuka - Placebo Test (RMSPE 5x)
% deviation from synthetic control
30
20
10
0
-10
-20
-30
1985
1990
1995
2000
2005
2010

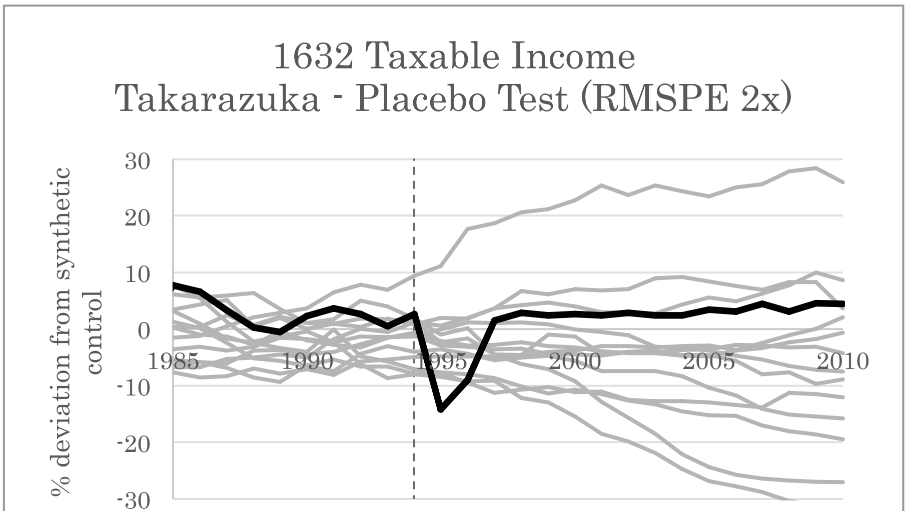
1632 Taxable Income
Takarazuka - Placebo Test (RMSPE 2x)
% deviation from synthetic control
30
20
10
0
-10
-20
-30
1985
1990
1995
2000
2005
2010

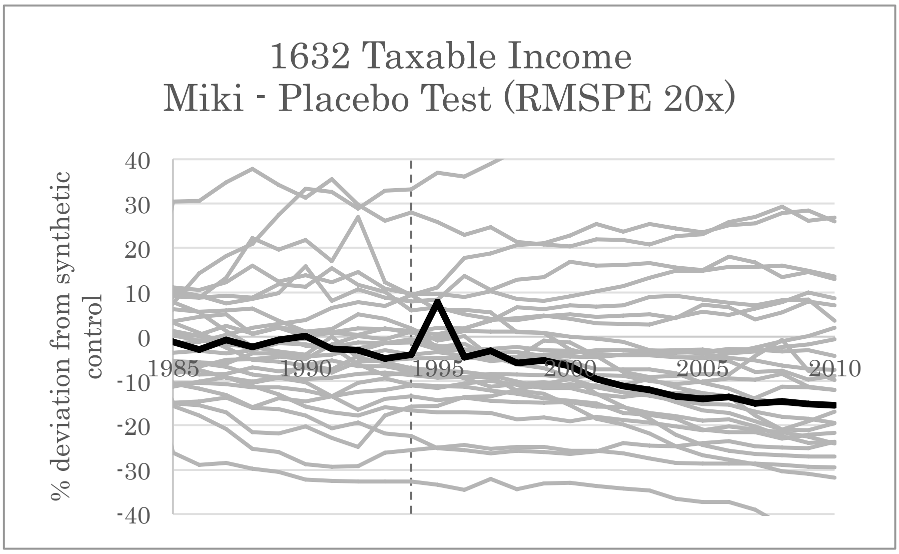
1632 Taxable Income
Miki - Placebo Test (RMSPE 20x)
% deviation from synthetic control
40
30
20
10
0
-10
-20
-30
-40
1985
1990
1995
2000
2005
2010

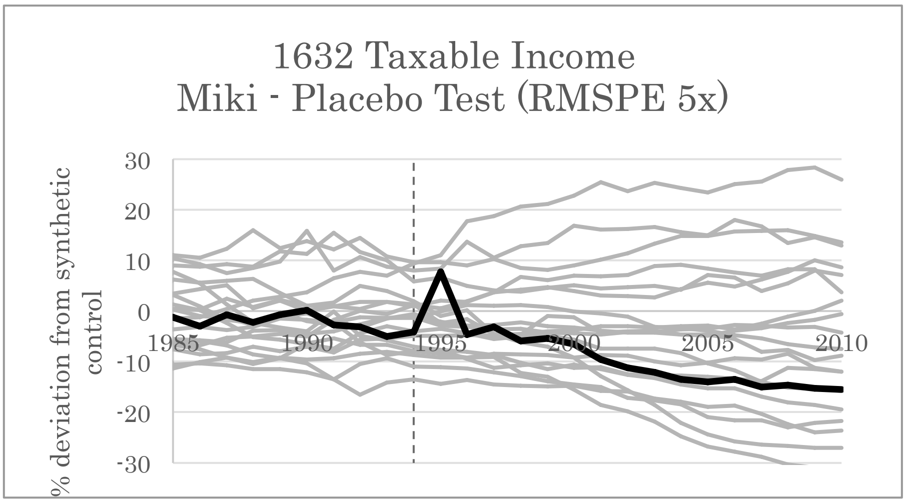
1632 Taxable Income
Miki - Placebo Test (RMSPE 5x)
% deviation from synthetic control
30
20
10
0
-10
-20
-30
1985
1990
1995
2000
2005
2010

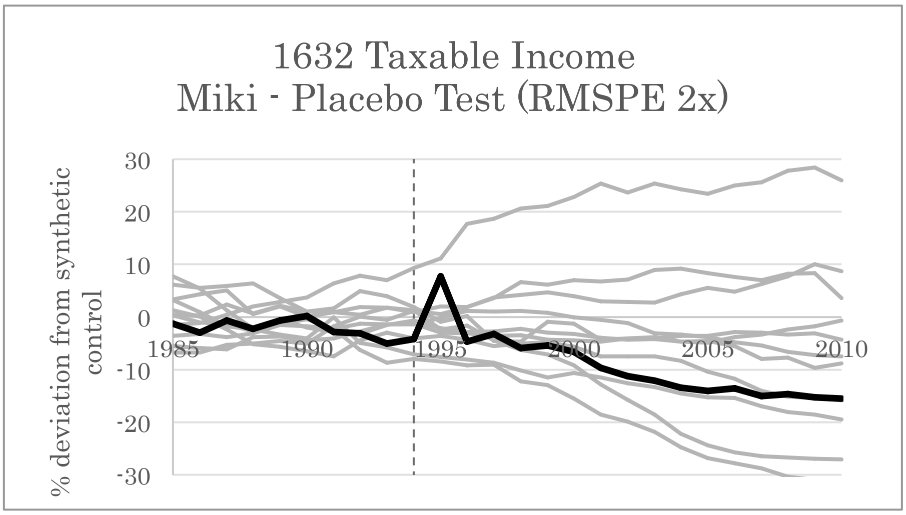
1632 Taxable Income
Miki - Placebo Test (RMSPE 2x)
% deviation from synthetic control
30
20
10
0
-10
-20
-30
1985
1990
1995
2000
2005
2010

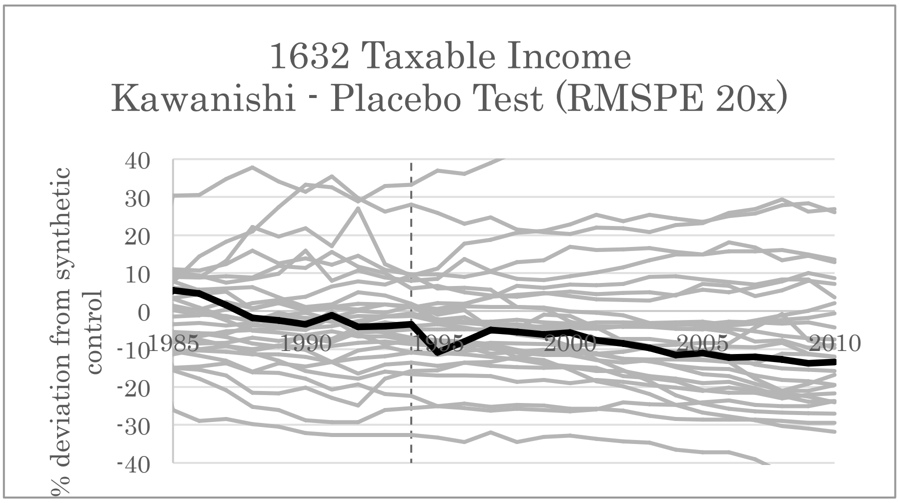
1632 Taxable Income
Kawanishi - Placebo Test (RMSPE 20x)
% deviation from synthetic control
40
30
20
10
0
-10
-20
-30
-40
1985
1990
1995
2000
2005
2010

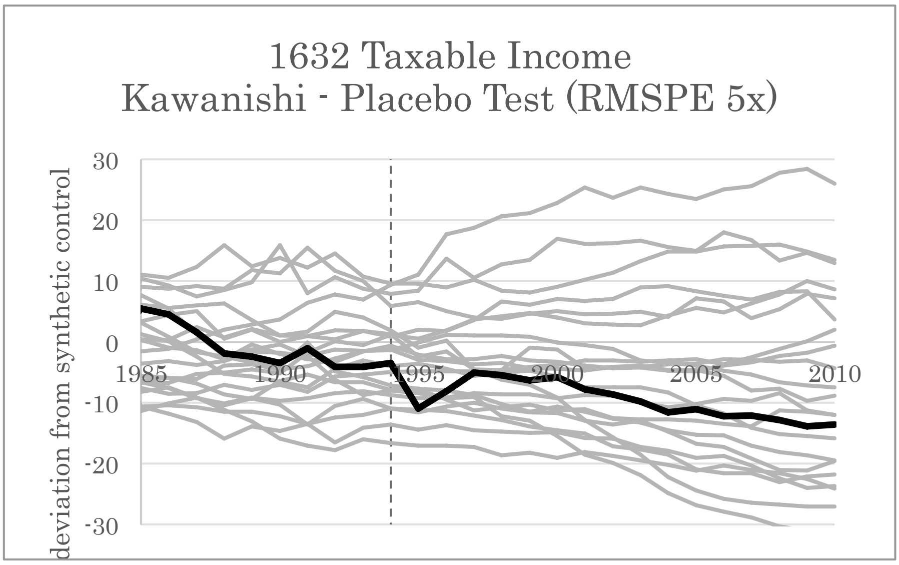
1632 Taxable Income
Kawanishi - Placebo Test (RMSPE 5x)
deviation from synthetic control
30
20
10
0
-10
-20
-30
1985
1990
1995
2000
2005
2010

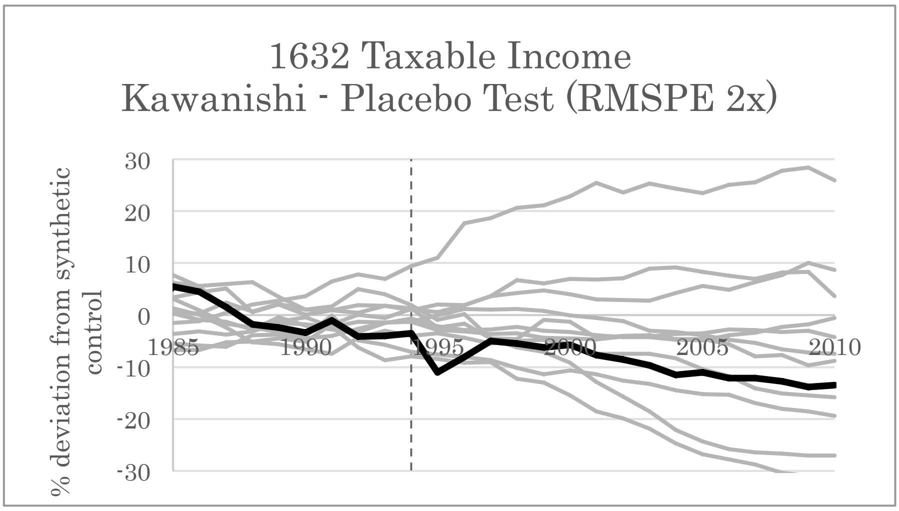
1632 Taxable Income
Kawanishi - Placebo Test (RMSPE 2x)
% deviation from synthetic control
30
20
10
0
-10
-20
-30
1985
1990
1995
2000
2005
2010

Table 1: Kobe Population in Register (A1352) Predictor Means

	Kobe		Average of 1641
**Variables**	**Real**	**Synthetic**	**Control Cities**
Total Population (A1001)	1418545	1414895	65004
Total 15~19 Population (A1016)	107381	109252	4866
Male 15~19 Population (A1017)	53786	56041	2492
Female 15~19 Population (A1018)	53594	53211	2374
Total 20~24 Population (A1019)	102883	109348	4424
Male 20~24 Population (A1020)	49627	54534	2248
Female 20~24 Population (A1021)	53256	54814	2175
Total 25~29 Population (A1022)	95346	107429	4477
Male 25~29 Population (A1023)	46511	52662	2261
Female 25~29 Population (A1024)	48835	54768	2216
Total 15~29 Population	305610	326029	13766
Male 15~29 Population	149924	163237	7001
Female 15~29 Population	155686	162793	6765
Total Under 15 Population (A1070)	284720	291263	13658
Total 15~64 Population (A1073)	983127	995204	44333
Total 65+ Population (A1076)	145022	125808	6942
Population in Register (A1352)	1415635	1415627	65463
Male Population in Register (A1353)	691868	691612	32219
Female Population in Register (A1354)	723768	724015	33245
Daytime Population (A1495)	1468094	1492182	64801
Male Daytime Population (A1496)	714958	746441	31792
Female Daytime Population (A1497)	753136	745742	33004
Households (A1498)	495233	505242	20581
General Households (A1499)	507853	519912	21113
Nuclear Households (A1506)	322856	315171	12125
Single Households (A1509)	107945	123519	4050
Total Land Area (B1562)	54392	71490	22079
Habitable Land Area (B1564)	30288	35458	7081
Taxable Income (C1632)	1910000000	1747204519	79453653
Taxable Income (C1633)	529201	543607	24975
Businesses (C1658)	82824	81580	3560
Secondary Businesses (C1690)	12522	11870	778

Table 1: Kobe Population in Register (A1352) Predictor Means (continued)

	Kobe		Average of 1641
**Variables**	**Real**	**Synthetic**	**Control Cities**
Employees (C1692)	719522	743958	29502
Secondary Employees (C1724)	179187	183880	9946
Tertiary Employees (C1725)	539756	558433	19388
Government Revenue (D2033)	648142857	492664453	18220858
Local Taxes (D2034)	213357143	193452956	6819331
Government Expenditure (D2055)	638357143	488100121	17678245
Labor Force (F2637)	662477	678544	32734
Employees (F2640)	634221	648820	31797
Unemployed (F2655)	28256	29724	936
Primary Employees (F2807)	7873	6525	3147
Adult Primary Employees (F2810)	6217	5204	2499
Elderly Primary Employees (F2813)	1656	1321	648
Secondary Employees (F2816)	182304	182495	10478
Adult Secondary Employees (F2819)	176250	177147	10156
Elderly Secondary Employees (F2822)	6054	5348	322
Tertiary Employees (F2825)	437812	457447	18085
Adult Tertiary Employees (F2828)	415109	439825	17276
Elderly Tertiary Employees (F2831)	22703	17622	809
Employers (F2876)	481924	509199	22270
Self Employed (F2891)	119140	101474	8020
Retail Shops (H3643)	20703	18396	915

All variables are averaged over the pre-treatment period.

Table 2. Data sources

Type	Souce	Code	Frequency	Duration	Location	Predictor
Total Population	Census	A1001	every 5 years	1980-2010	City/Ward	Yes
15 to 19 Population	Census	A1016	every 5 years	1980-2005	City/Ward	Yes
Male 15 to 19 Population	Census	A1017	every 5 years	1980-2005	City/Ward	Yes
Female 15 to 19 Population	Census	A1018	every 5 years	1980-2005	City/Ward	Yes
20 to 24 Population	Census	A1019	every 5 years	1980-2005	City/Ward	Yes
Male 20 to 24 Population	Census	A1020	every 5 years	1980-2005	City/Ward	Yes
Female 20 to 24 Population	Census	A1021	every 5 years	1980-2005	City/Ward	Yes
25 to 29 Population	Census	A1022	every 5 years	1980-2005	City/Ward	Yes
Male 25 to 29 Population	Census	A1023	every 5 years	1980-2005	City/Ward	Yes
Female 25 to 29 Population	Census	A1024	every 5 years	1980-2005	City/Ward	Yes
Total Under 15 Population	Census	A1070	every 5 years	1980-2010	City/Ward	Yes
Total 15 to 64 Population	Census	A1073	every 5 years	1980-2010	City/Ward	Yes
Total 65+ Population	Census	A1076	every 5 years	1980-2010	City/Ward	Yes
Population in Register		A1352	annual	1980-2010	City/Ward	Yes
Male Population in Register	Basic resident register population survey, MIC	A1353	annual	1980-2010	City/Ward	Yes
Female Population in Register		A1354	annual	1980-2010	City/Ward	Yes
Births	Vital statistics, MHLW	A1369	annual	1980-2009	City/Ward	No
Deaths	Vital statistics, MHLW	A1382	annual	1980-2009	City/Ward	No
Move In		A1465	annual	1980-2010	City/Ward	No
Male Move In		A1466	annual	1980-2010	City/Ward	No
Female Move In		A1467	annual	1980-2010	City/Ward	No
Move Out		A1468	annual	1980-2010	City/Ward	No
Male Move Out	Internal Migration in Japan Derived from the Basic Resident Registers, MIC	A1469	annual	1980-2010	City/Ward	No
Female Move Out		A1470	annual	1980-2010	City/Ward	No
Daytime Population	Census	A1495	every 5 years	1980-2005	City/Ward	Yes
Male Daytime Population	Census	A1496	every 5 years	1980-2005	City/Ward	Yes
Female Daytime Population	Census	A1497	every 5 years	1980-2005	City/Ward	Yes

Table 2. Data sources (continued)

Type	Souce	Code	Frequency	Duration	Location	Predictor
Households	Census	A1498	every 5 years	1980-2010	City/Ward	Yes
General Households	Census	A1499	every 5 years	1980-2010	City/Ward	Yes
Nuclear Households	Census	A1506	every 5 years	1980-2010	City/Ward	Yes
One-Person Households	Census	A1509	every 5 years	1980-2010	City/Ward	Yes
Total Land Area	Statistical reports on the land area by prefectures and municipalities in Japan, GSI	B1562	annual	1990	City/Ward	Yes
Habitable Land Area		B1564	annual	1990	City/Ward	Yes
Taxable Income	Statistics of taxation condition, etc in cities, town and villages, MIC	C1632	annual	1985-2010	City	Yes
Taxpayers		C1633	annual	1985-2010	City	Yes
Businesses	Report about statistics investigation of office and business, MIC	C1658	every 5 years	1981-2006	City/Ward	Yes
Secondary Businesses		C1690	every 5 years	1981-2006	City/Ward	Yes
Tertiary Businesses		C1691	every 5 years	1981-2006	City/Ward	Yes
Employees	Report about statistics investigation of office and business, MIC	C1692	every 5 years	1981-2006	City/Ward	Yes
Secondary Employees		C1724	every 5 years	1981-2006	City/Ward	Yes
Tertiary Employees		C1725	every 5 years	1981-2006	City/Ward	Yes
Product Shipments	Industrial statistical table, METI	C1795	annual	1980-2009	City/Ward	No
Manufacture Business	Industrial statistical table, METI	C1809	annual	1980-2009	City/Ward	No
Manufacture Employees	Industrial statistical table, METI	C1816	annual	1980-2009	City/Ward	No
Commerce Sales	Commercial statistical table, METI	C1827	every 3 years	1981-2006	City/Ward	No
Commercial Business	Commercial statistical table, METI	C1830	every 3 years	1982-2007	City/Ward	No
Commercial Employees	Commercial statistical table, METI	C1833	every 3 years	1982-2007	City/Ward	No
Government Revenue	Local finance statistic report, MIC	D2033	Annual	1980-2009	City	Yes
Local Taxes	Local finance statistic report, MIC	D2034	Annual	1980-2009	City	Yes
Government Expenditure	Local finance statistic report, MIC	D2055	Annual	1980-2009	City	Yes

Table 2. Data sources (continued)

Type	Souce	Code	Frequency	Duration	Location	Predictor
Labor Force	Census	F2637	every 5 years	1980-2005	City/Ward	Yes
Employees	Census	F2640	every 5 years	1980-2005	City/Ward	Yes
Unemployed	Census	F2655	every 5 years	1980-2005	City/Ward	Yes
Primary Employees	Census	F2807	every 5 years	1980-2005	City/Ward	Yes
Adult Primary Employees	Census	F2810	every 5 years	1980-2005	City/Ward	Yes
Elderly Primary Employees	Census	F2813	every 5 years	1980-2005	City/Ward	Yes
Secondary Employees	Census	F2816	every 5 years	1980-2005	City/Ward	Yes
Adult Secondary Employees	Census	F2819	every 5 years	1980-2005	City/Ward	Yes
Elderly Secondary Employees	Census	F2822	every 5 years	1980-2005	City/Ward	Yes
Tertiary Employees	Census	F2825	every 5 years	1980-2005	City/Ward	Yes
Adult Tertiary Employees	Census	F2828	every 5 years	1980-2005	City/Ward	Yes
Elderly Tertiary Employees	Census	F2831	every 5 years	1980-2005	City/Ward	Yes
Employers	Census	F2876	every 5 years	1980-2005	City/Ward	Yes
Self-Employed	Census	F2891	every 5 years	1980-2005	City/Ward	Yes
Retail Shops	Establishment and Enterprise Census, MIC	H3643	every 5 years	1981-2006	City/Ward	Yes
Restaurants		H3650	every 5 years	1981-2006	City/Ward	No
Big Retailers		H3651	every 5 years	1981-2006	City/Ward	No

Abbreviations
GSI; Geospatial Information Authority of Japan
METI; Ministry of Economy, Trade and Industry
MIC; Ministry of Internal Affairs and Communication
MHLW; Ministry of Health, Labor and Welfare

Appendix: Impacts of the Earthquake on Each Variable in Each Ward

Kobe

- Permanent negative impact on total population
    - -1.9% in 2000 (A1001)
    - -2.3% in 2000 (A1352)
    - -1.0% in 2010 (A1001)
    - -1.9% in 2010 (A1352)
- Permanent negative impact on both male and female population
- No impact on 15~29 year old population
- Permanent positive impact on elderly population
    - +7.1% in 2000
    - +15.6% in 2010
- Small Permanent negative impact on daytime population
    - -1% in 2000
    - -1.4% in 2005
- Permanent negative impact on taxpayer income
    - -3.7% in 2000
    - -7.8% in 2010
- Number of taxpayers, however, appears to recover
    - -2.7% in 2000
    - -0.8% in 2010
- Permanent positive impact on the number of unemployed
    - +27% in 2000
    - +29.2% in 2005

Kobe: Higashinada-ku

- Permanent positive impact on total population
    - 1.8% in 2000 (A1001)
    - 10.3% in 2010 (A1001)
- Permanent positive impact on 15~29 population
    - 5.1% in 2000
    - 12.7% in 2010
- Permanent negative impact on elderly population
    - -6.1% in 2000
    - -6.6% in 2010
- No impact on Daytime Population
- Permanent positive impact on Secondary Employees
    - +31.8% in 2001
    - +19.1% in 2006
- Small permanent positive impact on Tertiary Employees
    - +3.8% in 2001
    - +7.8% in 2006
- Permanent increase in the number of unemployed
    - likely due to population increase
    - +2.4% in 2000
    - +25.5% in 2005

Kobe: Nada-ku

- Permanent increase in total population
    - +3% in 2000 (A1001)

  - +3.1% in 2000 (A1352)
  - +15.6% in 2010 (A1001)
  - +13% in 2010 (A1352)
- Permanent increase in both male and female population
- Permanent increase in 15~29 population
  - +12.9% in 2000
  - +16.3% in 2005
- Permanent increase in Daytime Population
  - +3% in 2000
  - +7.6% in 2005
- No impact on the number of secondary businesses
- Permanent negative impact on the number of tertiary businesses
  - -8.7% in 2001
  - -3.7% in 2006
- Permanent positive impact on the number of unemployed
  - likely due to population increase
  - +20.3% in 2000
  - +11% in 2005

Kobe: Chuo-ku
- No permanent impact on total population
- Permanent decrease in Daytime Population
  - -12.6% in 2000
  - -10.9% in 2005

Kobe: Hyogo-ku
- Permanent decrease in total population
  - -16% in 2000 (A1001)
  - -15.3% in 2000 (A1352)
  - -20.1% in 2010 (A1001)
  - -18.9% in 2010 (A1352)
- Permanent decrease in both male and female population
- No impact on the number of secondary businesses
- Permanent decrease in the number of tertiary businesses
  - -7.1% in 2001
  - -5.6% in 2006
- Permanent increase in the number of unemployed
  - +17.3% in 2000
  - +40.8% in 2010

Kobe: Nagata-ku
- Permanent decrease in total population
  - -20.9% in 2000 (A1001)
  - -22.1% in 2000 (A1352)
  - -25.2% in 2010 (A1001)
  - -25.7% in 2010 (A1352)
- Permanent negative impact on both male and female population
- Permanent decrease in 15~29 population
  - -23.6% in 2000
  - -28.7% in 2005
- Permanent decrease in Daytime Population

    - -17.6% in 2000
    - -21.5% in 2005
- Permanent decrease in the number of Tertiary Businesses
    - -13.9% in 2001
    - -11.9% in 2006

Kobe: Suma-ku
- Permanent decrease in total population
    - -8.4% in 2000 (A1001)
    - -9.3% in 2000 (A1352)
    - -12.9% in 2010 (A1001)
    - -13.8% in 2010 (A1352)
- Permanent negative impact to both male and female population
- Permanent decrease in 15~29 population
    - -6.4% in 2000
    - -9.6% in 2005
- Permanent decrease in Daytime Population
    - -8.3% in 2000
    - -10.7% in 2005
- Permanent decrease in the number of Secondary Businesses
    - -29.6% in 2001
    - -32.8% in 2006
- Permanent increase in the number of unemployed
    - +16% in 2000
    - +11.6% in 2005

Kobe: Tarumi-ku
- Permanent increase in total population
    - Seems to have started prior to the earthquake
    - +19.2% in 2000 (A1001)
    - +19.7% in 2000 (A1352)
    - +16.2% in 2010 (A1001)
    - +17.2% in 2010 (A1352)
- Permanent positive impact in both male and female population
- Permanent increase in 15~29 population
    - +17.6% in 2000
    - +20.7% in 2005
- Permanent increase in Daytime Population
    - +18.2%
    - +16.5%
- Permanent increase in the number of unemployed
    - likely due to population increase
    - +30.9% in 2000
    - +25.9% in 2005

Kobe: Kita-ku
- Permanent increase in total population
    - May have started prior to the earthquake
    - +11.7% in 2000 (A1001)
    - +12% in 2000 (A1352)

  - o +14.9% in 2010 (A1001)
  - o +15.5% in 2010 (A1352)
- Permanent positive impact on both male and female population
- No impact on 15~29 population
- Permanent increase in the number of unemployed
  - o likely due to increase in population
  - o +29.3% in 2000
  - o +15.7% in 2005

Kobe: Nishi-ku
- Permanent increase in total population
  - o May have started prior to the earthquake
  - o +41.7% in 2000 (A1001)
  - o +39.4% in 2000 (A1352)
  - o +46.6% in 2010 (A1001)
  - o +44.3% in 2010 (A1352)
- Permanent positive impact on male and female population
- Permanent increase in 15~29 population
  - o +45.9% in 2000
  - o +61.1% in 2005
- Permanent increase in elderly population
  - o +16.6% in 2000
  - o +21.1% in 2010
- Permanent increase in Daytime population
  - o +44.8% in 2000
  - o +46.9% in 2005
- Permanent increase in the number of secondary businesses
  - o +27.8% in 2001
  - o +25.2% in 2006
- Permanent increase in the number of secondary employees
  - o +55% in 2001
  - o +55.6% in 2006
- Permanent increase in the number of unemployed
  - o likely due to population increase
  - o +40.3% in 2000
  - o +48.3% in 2005

Nishinomiya
- Short-term decline, but permanent increase in total population
  - o +4% in 2000 (A1001)
  - o +3.75 in 2000 (A1352)
  - o +9.1% in 2010 (A1001)
  - o +8.9% in 2010 (A1352)
- Permanent positive impact on both male and female population
- Permanent increase in 15~29 population
  - o +5.2% in 2000
  - o +5.8% in 2005
- Permanent decrease in elderly population
  - o -4.7% in 2000
  - o -3.3% in 2010
- Permanent decrease in daytime population

  - -5.4% in 2000
  - -3.5% in 2005
- Short-run impact on taxpayer income, but fully recovers
- Permanent decrease in the number of tertiary businesses
  - -19.4% in 2001
  - -16.4% in 2006
- Large temporary spike in government expenditure
- Mixed impact on the number of unemployed

Himeji
- No impact on population, taxpayer income, government expenditure, and the number of unemployed

<u>OTHER SURROUNDING TOWNS</u>

Amagasaki
- Bad fit with total population
- Permanent decrease in 15~29 population
  - -16.1% in 2000
  - -24.3% in 2005
- No impact on elderly population
- No impact on the number of secondary businesses
- Permanent decrease in the number of tertiary businesses
  - -15.7% in 2001
  - -21.8% in 2006
- No impact on the number of unemployed

Akashi
- Permanent increase in total population
  - +8.5% in 2000
  - +6.1% in 2010
- No impact on 15~29 Population
- No impact on elderly population
- Permanent increase in daytime population
  - +9.2% in 2000
  - +7.5% in 2005
- Short-run negative impact on taxpayer income, but fully recovers
- Short-run negative impact on # of taxpayers but fully recovers
- No impact on the number of tertiary businesses
- No impact on the number of tertiary employees
- Permanent increase in government expenditure
- Permanent increase in the number of unemployed
  - +15.4% in 2000
  - +7% in 2005

Sumoto
- No impact on population
- Temporary increase in taxpayer income in the late '90's.
  - Returns to normal by 2005
- No impact on the number of secondary businesses

- Temporary increase in Tertiary Businesses prior to the quake
    - Bridge related?
    - Permanent Increase in the number of secondary employees
        - +27.4% in 2001
        - +21.8% in 2006
    - Small permanent increase in tertiary employees
        - +3.5% in 2001
        - +8.3% in 2006
    - Large increase in government expenditure
    - No impact on the number of unemployed

Ashiya

- Note Ashiya is very difficult to match
    - this is likely due to its Beverly Hills esque nature.
- Temporary decline in total population
    - -7.1% in 2000 (A1001)
    - -2.7% in 2010 (A1001)
- Permanent decline in the elderly population
    - -7.3% in 2000
    - -4.9% in 2010
- Temporary decline in the number of unemployed
    - -14.8% in 2000
    - +3.3% in 2005

Itami

- Small impacts related to the construction of the Kansai airport?
- No impact on population
- Temporary Increase in the number of secondary employees
    - +12.3% in 2001
    - -1% in 2006
- Permanent decline in the number of tertiary employees
    - -14.6% in 2001
    - -10.9% in 2006
- Short-run increase in government expenditure following the quake
- Permanent increase in the number of unemployed?
    - +11.1% in 2000
    - 10.1% in 2005

Takarazuka

- Permanent increase in the total population
    - +2.5% in 2000 (A1001)
    - +4.4% in 2000 (A1352)
    - +6.9% in 2010 (A1001)
    - +9.1% in 2010 (A1352)
- Permanent increase in both the male and female population
- Permanent decrease in 15~29 population
    - -7.4% in 2000
    - -9.8% in 2005
- No impact on elderly population
- Temporary decline in taxpayer income, but fully recovered
- Temporary decline in the number of taxpayers, but fully recovered

- Temporary spike in government expenditure
- Permanent increase in the number of unemployed.
  - +10.2% in 2000
  - +7.6% in 2005

Miki
- Permanent decline in total population
  - -5.7% in 2000 (A1001)
  - -6.3% in 2000 (A1352)
  - -11.4% in 2010 (A1001)
  - -11.6% in 2010 (A1352)
- Permanent decline in both the male and female population
- Permanent decline in 15~29 population
  - -4.5% in 2000
  - -5.5% in 2005
- No impact on the elderly population
- No impact on daytime population
- Permanent decline in taxpayer income
  - -6.6% in 2000
  - -15.5% in 2010
- Permanent decline in the number of taxpayers
  - -7.2% in 2000
  - -11.7% in 2010
- Permanent increase in the number of secondary businesses
  - +31.9% in 2001
  - +27.1% in 2006
- No impact on the number of tertiary businesses
- Permanent increase in the number of secondary employees
  - +15.6% in 2001
  - +18.7% in 2006
- No impact on the number of tertiary employees
- Temporary increase in government expenditure
- Little impact on the number of unemployed

Kawanishi
- No impact on total population
- Permanent decrease in 15~29 population
  - -11.5% in 2000
  - -16.5% in 2005
- Permanent increase in elderly population
  - +9.1% in 2000
  - +9.9% in 2010
- Permanent decline in taxpayer income
  - -5.8% in 2000
  - -13.5% in 2010
- Permanent decline in the number of taxpayers
  - -4% in 2000
  - -8.4% in 2010
- Permanent increase in the number of unemployed
  - +2.6% in 2000
  - +7.3% in 2010
